# Different components of cognitive-behavioral therapy affect specific cognitive mechanisms

**DOI:** 10.1126/sciadv.adk3222

**Published:** 2024-03-27

**Authors:** Agnes Norbury, Tobias U. Hauser, Stephen M. Fleming, Raymond J. Dolan, Quentin J. M. Huys

**Affiliations:** ^1^ Applied Computational Psychiatry Lab, Max Planck Centre for Computational Psychiatry and Ageing Research, Queen Square Institute of Neurology and Mental Health Neuroscience Department, Division of Psychiatry, University College London, London, UK.; ^2^ Max Planck Centre for Computational Psychiatry and Ageing Research, Queen Square Institute of Neurology and Mental Health Neuroscience Department, Division of Psychiatry, University College London, London, UK.; ^3^ Wellcome Centre for Human Neuroimaging, University College London, London, UK.; ^4^ Department for Psychiatry and Psychotherapy, and German Center for Mental Health (DZPG), University of Tübingen, Germany.; ^5^ Department of Experimental Psychology, University College London, London, UK.

## Abstract

Psychological therapies are among the most effective treatments for common mental health problems—however, we still know relatively little about how exactly they improve symptoms. Here, we demonstrate the power of combining theory with computational methods to parse effects of different components of cognitive-behavioral therapies onto underlying mechanisms. Specifically, we present data from a series of randomized-controlled experiments testing the effects of brief components of behavioral and cognitive therapies on different cognitive processes, using well-validated behavioral measures and associated computational models. A goal setting intervention, based on behavioral activation therapy activities, reliably and selectively reduced sensitivity to effort when deciding how to act to gain reward. By contrast, a cognitive restructuring intervention, based on cognitive therapy materials, reliably and selectively reduced the tendency to attribute negative everyday events to self-related causes. The effects of each intervention were specific to these respective measures. Our approach provides a basis for beginning to understand how different elements of common psychotherapy programs may work.

## Teaser

Computational measures of cognition, drawn from psychological theory, may help reveal how psychotherapy treatments work.

## INTRODUCTION

**Fig. 1.**
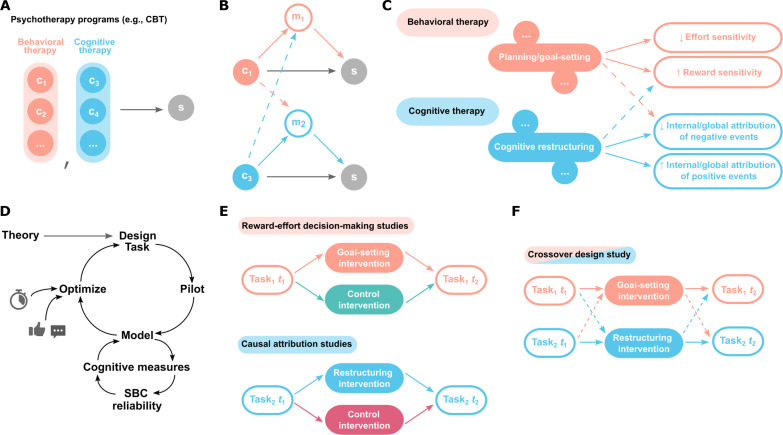
The use of precise and reliable cognitive measures from computational cognitive science may help shed light on mechanisms targeted by components of common psychological therapies. (**A**) At present, the majority of our causal knowledge regarding psychotherapy outcomes (solid arrow) concerns how different treatment programs (e.g., cognitive-behavioral therapy or CBT), made up of multiple components (*c_x_*), affect symptom levels (*s*). (**B**) Ideally, this could be decomposed into how different components affect symptoms via specific underlying mechanisms (*m_x_*). If different treatments work by at least partially distinct mechanisms, then this yields the opportunity for treatment personalization (dotted arrows, effects not predicted under a specific mechanism model). (**C**) Potential mechanisms by which different components of psychological therapies improve symptoms may be drawn from psychological theory. Here, we tested whether interventions based on two different therapy components influence different underlying mechanisms (solid lines) and whether effects are specific to these mechanisms (dotted lines). (**D**) To generate precise and reliable estimates of potential cognitive mechanisms, task design and analysis methods underwent several cycles of design optimization. This included simulation-based calibration (SBC) analysis of model-based inference procedures and assessment of observed test-retest reliability of model-based measures, alongside optimization for task brevity and user-acceptability. Specifically, we developed a gamified reward-effort decision-making paradigm (task_1_) that yields robust measurement of reward and effort sensitivity when deciding whether specific actions are worth taking and a causal attribution task (task_2_) that measures latent tendency to attribute positive and negative events to internal (versus external) and global (versus specific) causes. (**E**) Next, we tested whether these sets of measures were sensitive to interventions based on relevant therapy components in a series of randomized, controlled experiments. (**F**) Last, we used a crossover design to test whether effects of interventions were specific to their proposed cognitive substrates.

There is compelling evidence that psychotherapy programs as a whole are effective treatments for common mental health problems (Fig. 1A) (*1*–*3*). However, psychotherapy programs are complex, multicomponent interventions, and we still lack an understanding of how different components of these programs work (Fig. 1B) (*4*–*6*). Such insight is vital, as understanding the mechanisms underlying treatment response is one of the most promising routes to achieving many of the goals of mental health research—including increasing efficacy, engagement, and, ideally, theoretically-informed treatment personalization (*7*). Here, we argue that developments in the cognitive sciences concerning how to use robustly designed behavioral tasks, in combination with rigorous modeling procedures that generate precise and reliable measures of cognitive processes, can accelerate progress toward these goals (*8*–*11*).

In line with recent calls to the research community (*12*, *13*), we take the pragmatic approach of starting from psychological therapies supported by a strong evidence base and working back to theories regarding the mechanisms by which they work. While cognitive and behavioral therapies are often administered together as part of the same treatment program [e.g., (*14*)], they differ in underlying theory–for example, the primacy of behavioral versus cognitive changes in fostering improved mood (*15*, *16*). This distinction offers an opportunity to test whether they appear to work via different mechanisms and whether they are specific in their action via these proposed mechanisms.

Here, we present data from a series of studies investigating the mechanisms by which specific components of behavioral and cognitive therapies are proposed to work. We focus on a remote (online) setting, given the relative ease of delivering content in a standardized way and the likely utility of a modular approach to treatment personalization for digitally delivered therapies (see Discussion). The first set of studies consisted of developing robust assessments of cognitive processes thought to be targeted by different components of cognitive and behavioral therapies (Fig. 1C). Each assessment combined an optimized behavioral task with a theoretically informed computational model, affording precise and reliable measurement of multiple different cognitive mechanisms (Fig. 1D). Specifically, one set of measures was designed to probe constructs relevant to the goal setting component of behavioral activation (“how to decide when rewards are worth exerting effort for”) and the other constructs relevant to the cognitive restructuring component of cognitive therapy (“how to reason about likely causes of things that happen to us”). In a second group of studies, we examined the extent to which these measures were sensitive to interventions derived from each therapy component (Fig. 1E). In a third study, we examined whether changes in cognitive mechanisms identified in the previous studies were specific to that particular intervention type (Fig. 1F). Last, we used data from studies two and three to explore to what extent individual differences in symptom profiles may relate to the magnitude of effects of each intervention on underlying cognitive mechanisms.

The overarching aim of this work is to demonstrate how creating reliable and acceptable measures of cognitive processes, drawn from relevant psychological theory, might help identify mechanisms of psychological interventions. Although it will be necessary to translate any findings into real-world clinical settings in the future, we believe that these studies represent an important step toward establishing how psychotherapy treatments work, and who they may be most likely to work for.

## RESULTS

### Developing useful measures for psychotherapy process research

**Fig. 2.**
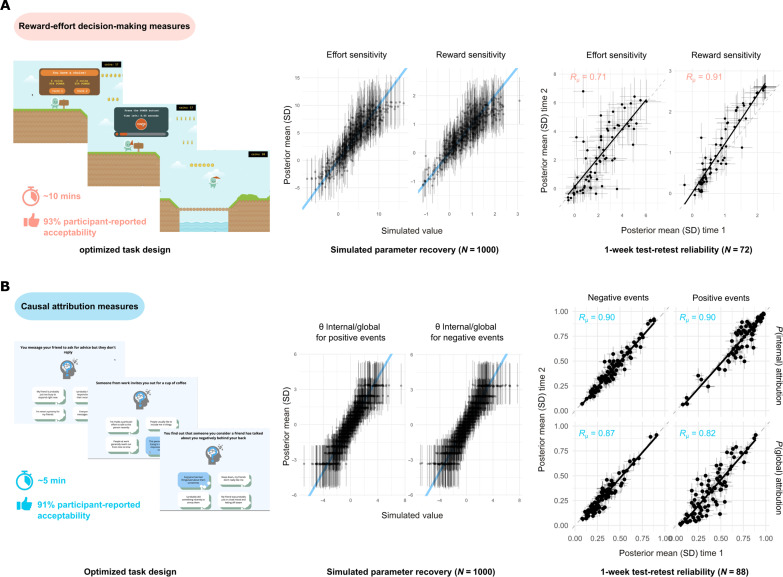
Optimized task designs for measuring cognitive mechanisms relevant to goal setting and cognitive restructuring components of behavioral activation and cognitive therapies. (**A**) The gamified reward-effort decision-making task, which measures sensitivity to required effort and potential reward when deciding between different effortful actions (for further details, see screenshots in Supplement 2 or play a demo version at https://modcomp-i1.web.app/). (**B**) The causal attribution task, which measures latent tendency to attribute positive and negative everyday events to internal (versus external) and global (versus specific) causes (for further details, see screenshots in Supplement 3 or play a demo version at https://modcomp-ca2.web.app/). Left: Representative screenshots of the final task versions, alongside average task completion times and % user-acceptability ratings. Middle and right: Psychometric properties of derived cognitive measures (independent parameter recovery during SBC analysis and observed test-retest reliability). θ, parameters describing latent tendency to endorse internal/global attributions for positive and negative events; *R*_μ_, posterior mean estimates for observed test-retest reliability of each model parameter.

Several considerations that motivated and guided our approach are worth outlining up-front. First, to ensure reliable measurement of relevant cognitive mechanisms, each set of tasks and measures went through extensive rounds of design and analytic optimization before proceeding with the main studies (Fig. 1D). To derive our computational measures, we used an analytical approach [Hierarchical Bayesian analysis; (*17*, *18*)] previously shown to substantially increase the reliability of individual-level parameter estimates, by allowing information to be shared between relevant levels of analysis, and better accounting for measurement error (*19*–*22*). Second, for each prospective task design, rigorous testing of inference (model-fitting) procedures was carried out via simulation-based calibration (SBC) (*23*), a general method for validating generative Bayesian algorithms using simulated datasets and posterior inference (Materials and Methods and fig. S1) (*24*). Following this analysis, the observed test-retest reliability of parameter estimates from the chosen design was explicitly assessed in our target population (Fig. 2, A and B).

Equally important as the above analytic considerations, useful individual difference measures should evoke robust differences between participants (*25*, *26*) and be acceptable (ideally engaging!) to their end users (*27*). Initial task development therefore proceeded via multiple informal cycles of “user-in-the-loop” design optimization. Specifically, to maximize the magnitude of observable individual differences, care was taken to minimize range restriction (floor/ceiling) effects (*25*, *26*). In light of qualitative feedback from our participants and previous mental health studies using online tasks (*28*), major design optimization targets were task brevity (minimum trials to reliably detect target parameters) and basic levels of acceptability (“I would be willing to play this game again in the future”; Fig. 2, A and B). We attempted to increase the engagingness of our tasks via two different approaches: gamification (*29*, *30*) and providing opportunity for self-reflection or insight (*28*, *31*). Last, where possible, we also tested measures for robustness to important sociodemographic differences between participants (Materials and Methods and fig. S2).

Although the above strategies necessarily involve some trade-offs against ideal psychometric properties and other important features (e.g., construct validity; see Discussion), we believe that consideration of these issues at early stages is vital to the development of potentially clinically useful measures (*32*).

### Testing effects of interventions based on different components of cognitive-behavioral therapy on their proposed cognitive mechanisms

To test whether interventions derived from different components of behavioral activation and cognitive restructuring therapies affect their proposed cognitive mechanisms, we next conducted a set of studies in which participants completed the relevant task-based assessment twice, with 1:1 random assignment to either the active intervention or a well-matched control intervention in between (i.e., a mixed within/between-subjects design; Fig. 1E). In all cases, initial discovery experiments were followed up with replication tests, to assess the reliability of findings.

At the end of each study, participants provided self-reported demographic and clinical information. Participants for all studies are described in Table 1. Samples showed some evidence of self-selection for interest in mental health research, given, on average, 45% of participants reported previous treatment for a mental health problem and moderate endorsement of current depression and social anxiety symptoms. Across studies, 31 and 56% of participants met criteria for clinical levels of low mood and social anxiety, respectively (see fig. S3). Samples were relatively well-balanced in terms of age, gender, and neurodiversity but were predominantly white, employed, and of relatively secure financial and housing status.

**Table 1.** Self-reported demographic and clinical data for all study participants. For reward-effort decision-making and causal attribution studies, samples 1 and 2 represent the initial discovery and replication samples, respectively. For the crossover study, sample 1 represents individuals who were randomized to the reward-effort decision-making task, and sample 2 represents individuals who were randomized to the causal attribution task. Response categories for employment, financial, and housing status were based on those described in (*80*). Employment status categories were employed (including full-time and part-time employment), unemployed (job seekers and those unemployed owing to ill health), and not seeking employment (stay-at-home parents, students, and retirees). Housing status categories were homeowner (including those with a mortgage), tenant, and other (living with family or friends, homeless, or living in a hostel). Neurodivergence was defined as a term for when someone processes or learns information in a different way to that which is considered typical: common examples include autism and attention-deficit/hyperactivity disorder. Categories for previous mental health treatment were talking therapy (including cognitive-behavioral therapies), medication, self-guided (e.g., workbooks or apps), or other. PHQ9 total, Physician’s Health Questionnaire nine-item measure of depressed mood total score (possible range, 0 to 27). AMI: behavioral, Apathy Motivational Index behavioral amotivation subscale score (possible range, 0 to 4, mean score across six items). miniSPIN total, mini Social Phobia Inventory total score (possible range, 0 to 12). DAS-SF total, Dysfunctional Attitude Scale short-form total score (possible range, 9 to 36). -, questionnaire not administered in this sample.

		Reward-effort sample 1	Reward-effort sample 2	Causal attribution sample 1	Causal attribution sample 2	Crossover study sample 1	Crossover study sample 2
Age (years)	*N*	100	102	100	100	197	208
	Mean (SD)	35.3 (11)	40.1 (11.6)	36 (9.5)	38.5 (11.4)	37.4 (12.8)	38.7 (12.3)
	Range	19–60	18–64	19–60	19–63	18–65	18–65
Gender	Woman	77 (79%)	61 (60%)	66 (66%)	44 (44%)	99 (52%)	112 (54%)
	Man	20 (20%)	40 (39%)	30 (30%)	56 (56%)	92 (48%)	94 (45%)
	Nonbinary of other	1 (1%)	1 (1%)	4 (4%)	0 (0%)	1 (1%)	2 (1%)
Race/ ethnicity	White	80 (82%)	87 (86%)	80 (80%)	85 (85%)	164 (85%)	165 (80%)
	Asian	5 (5%)	6 (6%)	7 (7%)	7 (7%)	15 (8%)	16 (8%)
	Black	1 (1%)	4 (4%)	3 (3%)	2 (2%)	2 (1%)	11 (5%)
	Mixed	7 (7%)	3 (3%)	4 (4%)	4 (4%)	7 (4%)	9 (4%)
	Other	5 (5%)	1 (1%)	6 (6%)	2 (2%)	4 (2%)	7 (3%)
Employment status	Employed	64 (65%)	69 (68%)	79 (80%)	68 (68%)	142 (74%)	156 (75%)
	Unemployed	13 (13%)	11 (11%)	9 (9%)	10 (10%)	19 (10%)	18 (9%)
	Not seeking	21 (21%)	22 (22%)	11 (11%)	22 (22%)	31 (16%)	34 (16%)
Financial status	Doing okay	52 (53%)	39 (38%)	46 (46%)	49 (49%)	91 (47%)	111 (53%)
	Just about getting by	31 (32%)	43 (42%)	38 (38%)	36 (36%)	70 (37%)	74 (36%)
	Struggling	15 (15%)	20 (20%)	15 (15%)	15 (15%)	31 (16%)	23 (11%)
Housing status	Homeowner	44 (45%)	47 (46%)	42 (42%)	48 (48%)	85 (44%)	94 (45%)
	Tenant	29 (30%)	39 (38%)	48 (48%)	38 (38%)	71 (37%)	77 (37%)
	Other	25 (26%)	16 (16%)	9 (9%)	14 (14%)	36 (19%)	37 (18%)
Neurodivergence	Yes	19 (19%)	18 (18%)	15 (15%)	10 (10%)	37 (19%)	31 (15%)
	No	72 (73%)	80 (78%)	80 (81%)	87 (87%)	146 (76%)	172 (83%)
	Prefer not to say	7 (7%)	4 (4%)	5 (5%)	3 (3%)	9 (5%)	5 (2%)
Previous treatment for a mental health problem	Yes	49 (50%)	42 (41%)	52 (53%)	37 (37%)	97 (51%)	76 (37%)
	No	47 (48%)	58 (57%)	48 (48%)	55 (55%)	93 (48%)	128 (62%)
	Prefer not to say	2 (2%)	2 (2%)	0 (0%)	8 (8%)	1 (1%)	4 (2%)
If yes, type of treatment (all that apply)	Talking therapy	38 (39%)	31 (30%)	36 (36%)	26 (26%)	72 (38%)	57 (27%)
	Medication	35 (36%)	29 (28%)	35 (35%)	27 (27%)	70 (37%)	51 (25%)
	Self-guided	23 (23%)	20 (20%)	21 (21%)	18 (18%)	38 (20%)	32 (15%)
	Other	7 (7%)	2 (2%)	4 (4%)	1 (1%)	9 (5%)	6 (3%)
PHQ9 total	Mean (SD)	9.1 (6.7)	7.6 (6)	7.7 (6.1)	6.9 (6.3)	7.4 (6.1)	6.7 (5.6)
AMI behavior	Mean (SD)	1.8 (0.8)	1.8 (0.8)	-	-	1.6 (0.7)	1.6 (0.8)
DAS-SF total	mean (SD)	-	-	19.1 (4.5)	19.3 (4.8)	19.4 (5)	19.1 (4.8)
miniSPIN total	mean (SD)	7.2 (3.3)	7 (3.2)	6.1 (3.3)	5.4 (3.8)	5.7 (3.3)	5.6 (3.5)

### Effects of a goal setting intervention derived from behavioral activation therapy on reward-effort decision-making

#### Role of goal setting in behavioral activation therapy

The use of activity scheduling and goal setting exercises is a core element of behavioral activation therapy for low mood (*16*). Acting according to a plan, rather than relying on internal state or mood, is thought to increase the likelihood of both acting and subsequently experiencing natural rewards, resulting in a positive reinforcement loop that serves to promote further activity and reward experience (*33*). In theory, acting according to a predetermined plan could boost activity levels either by making potential rewards more salient (increasing reward sensitivity) or by lowering the perceived level of effort required (decreasing effort sensitivity), when deciding if a particular action is worth taking (Fig. 1C) (*9*, *10*).

#### Investigating the effects of a goal setting intervention on reward-effort decision-making

Here, we made use of the fact that reward-effort decision-making has been well-studied in cognitive neuroscience [e.g., (*34*–*36*)]. Starting from a previously validated task design (*36*), in conjunction with the recent introduction of online game engines into behavioral neuroscience research (*37*), we developed a gamified task that was short, acceptable to users, and could reliably identify reward and effort sensitivity parameters from choice data (Fig. 2A and Materials and Methods). Briefly, on each trial, participants were asked to choose between two options, which varied both in terms of required physical effort (fast presses on a mouse or touchscreen) and offered reward amount (number of game coins, which were converted into a cash bonus at the end of the study). Choices were always nondominated (the higher reward option required greater effort), except for two “catch” trials used as internal attention checks (see Materials and Methods). After choosing an option, participants had to exert the required effort within a time limit (10 s) to gain the reward. After each block of trials (four per task), participants were asked to rate their sense of achievement on successful effort exertion, sense of pleasure in gaining rewards, and boredom levels, using an interactive slider.

The goal setting intervention was based on exercises delivered as part of behavioral activation therapy for low mood (*16*) and consisted of text describing the importance of setting realistic (achievable) goals, followed by a short comprehension quiz and a modified version of the task where participants were asked to apply this information (see Materials and Methods, Supplement 2). Akin to the use of activity scheduling worksheets during behavioral activation therapy, when completing the game for the second time, participants in the goal setting condition were asked to set a goal (number of coins they would like to earn, out of the maximum possible available) before completing each block. Within each block, progress toward their goal was then tracked visually across trials. The control intervention consisted of matched-length information about different kinds of computer games, also followed by a quiz [for intervention reading times and quiz results; see fig. S5 (C and D)]. Participants in the control condition were asked to rate how much they enjoyed different kinds of games at the start of each block, but the task was otherwise unchanged.

#### Goal setting decreases effort sensitivity during reward-effort decision-making

**Fig. 3.**
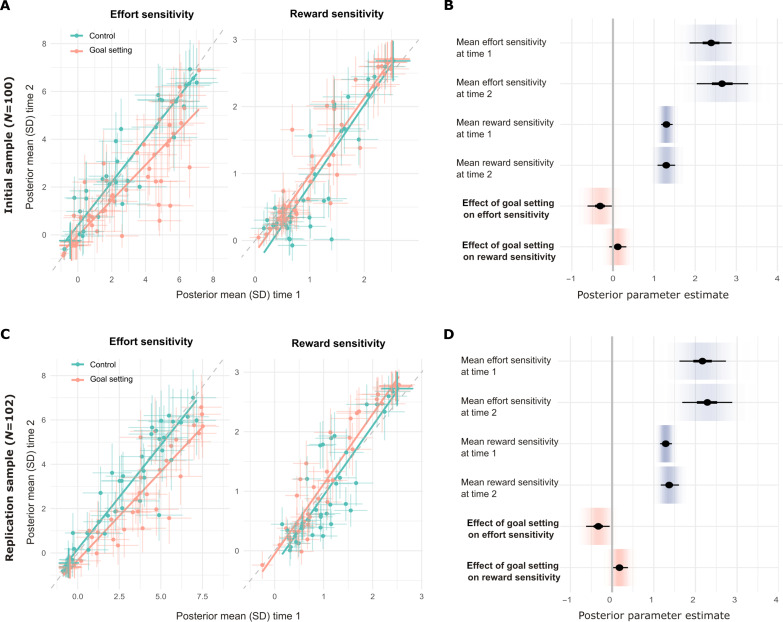
A goal setting intervention based on principles of behavioral activation therapy resulted in a decrease in effort sensitivity during reward-effort decision-making, compared to a well-matched control condition. (**A**) Posterior mean (and SD) parameter estimates for each participant at time 1 (preintervention) versus time 1 (postintervention), by intervention condition, in the initial discovery sample (goal setting intervention, *N* = 49; control intervention, *N* = 51). Effort sensitivity parameter estimates are in inverse units of task reward (range, three to seven coins), and reward sensitivity parameter estimates are in inverse units of required effort (range, 0.25 to 0.95% maximal effort). Lines of best fit for posterior mean parameter estimates at time 1 versus time 2 for individuals in each intervention group are plotted for illustration purposes. (**B**) Posterior parameter estimates for group means (over all participants/intervention conditions) for each parameter at each time point and the additional effects of goal setting intervention in active group participants at time 2, in the initial discovery sample. Thick inner lines represent 50%, thin outer lines represent 90% CIs, the point estimate is the mean, and shading represents posterior probability density. For visualization purposes, intervention effects (bold text) have been scaled by the square root of the mean posterior variance estimates for parameter values at time 2, making them roughly equivalent to standardized mean differences (SMDs). (**C**) The same plot as (A), for the independent replication sample (goal setting, *N* = 50; control, *N* = 52). (**D**) The same plot as (B), for the independent replication sample.

In both initial discovery (*N* = 100) and replication samples (*N* = 102), linear mixed-effects modeling of individual trial data revealed a significant interaction between intervention condition and time point (pre- versus postintervention) on proportionate choice of higher-effort higher-reward options (*F*_1,8697_ = 14.5, *F*_1,8871_ = 34.8; *P* < 0.001)—with greater choice of higher-effort options at time 2 in the goal setting group (Supplementary Results and fig. S4). Analysis via our prespecified Hierarchical Bayesian analysis model (as developed during the task design optimization process) revealed that, in both samples, this was due to a decrease in effort sensitivity at time 2 for individuals who completed the goal setting intervention (posterior means for group-level effect of the goal setting intervention on effort sensitivity at time 2 = −0.31 [90% posterior credible interval (CI) −0.55,−0.09], −0.37 [90% posterior CI −0.61,−0.14]; Fig. 3 and table S1). In the replication sample, there was also evidence for a small increase in reward sensitivity in the goal setting group at time 2 (mean = 0.18 [90% posterior CI 0.03,0.34]). Mean posterior predictive accuracy of the model for each sample was 0.82 (SD 0.14) and 0.83 (SD 0.15), and pseudo-*r*^2^ values (reflecting relative proportion of variance in choice behavior explained, compared to a chance model) were 0.49 and 0.50, respectively.

#### Goal setting changes subjective evaluation of effort expenditure and reward receipt

In line with the theory that goal setting leads to a decrease in effort sensitivity when deciding to act, in turn leading to greater experience of reward, in both samples participants in the goal setting condition reported greater sense of achievement on successful effort exertion (*F*_1,97_ = 21.0, *F*_1,100_ = 23.7), greater pleasure on gaining rewards (*F*_1,97_ = 20.4, *F*_1,100_ = 12.7), and lower boredom levels, during the second game (*F*_1,97_ = 33.8, *F*_1,100_ = 4.2; all *P* < 0.05; Supplementary Results and fig. S4).

#### Emphasizing the importance of setting achievable goals leads to increased effort expenditure over time

Consistent with the importance behavioral activation therapy exercises place on both setting achievable goals and gradual increasing effort expenditure over time (*16*), we found that participants in the goal setting condition tended to both exceed their goals within each block and increase the ambitiousness of their goals across task blocks (*F*_2.4,236_ = 19, *F*_2.5,245_ = 8.9; *P* < 0.001; Supplementary Results and fig. S5).

### Effects of a cognitive restructuring intervention on causal attribution of positive and negative events

#### Role of cognitive restructuring in cognitive therapy

A core idea underlying cognitive therapy is that it is often how we interpret things that happen to us, rather than the events themselves, that shapes how we end up feeling (*15*). In particular, one formulation (learned helplessness theory) suggests that, in some individuals, persistent low mood results from a heightened tendency to attribute negative events to causes which are internal (related to the self, compared the outside world), global (likely to be active in all situations rather than this specific one alone), and stable (likely to persist in time rather than change in the future) (*38*). Therefore, a key focus of cognitive restructuring is training individuals to identify unhelpful attributions and practising consideration of alternative and helpful explanations (“reappraisal”) (*39*).

While there is robust evidence of heightened attribution of positive events to internal and global causes in healthy individuals (an effect which has been interpreted as a self-serving or self-protective bias), overly internal and global attributions of negative events have been identified in currently depressed individuals and predicts future depressed mood (*40*, *41*). However, it is not clear (i) the extent to which addressing these different dimensions (internality and globality) is important in cognitive restructuring and (ii) the extent to which improvements in mood relate to a decreased tendency to make “depressogenic” attributions (internal/global attributions of negative events) versus increased use of self-protective or compensatory strategies (internal/global attributions of positive events) (Fig. 1C) (*7*, *42*).

#### Investigating the effects of a cognitive restructuring intervention on causal attribution

Here, we present data from a novel hybrid self-report/task measure (“causal attribution task”), developed from an analysis of previous scenario-based attribution tasks, item-response theory-based optimization, and consideration of sensitivity to potential sociodemographic moderators (age, gender, functional disability/neurodivergence, and minoritized group status; Materials and Methods and fig. S2). Briefly, participants were presented a series of brief descriptions of events and asked to choose which of four listed causal explanations they thought the most likely, if such an event had happened to them. Half the events were positive and half negative, and the four potential explanations varied orthogonally in terms of describing internal (versus external) and global (versus specific) causes. Extensive pilot testing revealed that data from two alternative task versions could be used to reliably identify parameters governing probability of endorsement of an internal (versus external) and global (versus specific) causes, separately for positively and negatively valenced events (Materials and Methods and Fig. 2B). Of note, attribution tendencies on this task are significantly correlated with self-reported negative beliefs about the self on a Beckian measure [the dysfunctional attitudes scale (DAS); see fig. S8 and Fig. 6], suggesting that it is able to tap aspects of cognition relevant to both dysfunctional self-beliefs and depressogenic attributional style.

The cognitive restructuring intervention was based on materials describing cognitive therapy for low mood (*15*) and consisted of information about a cognitive model of mood (link between interpretations of events and feelings), interactive exercises identifying helpful and unhelpful attributions of the same events, inviting people to practise generating alternative explanations for recent events in their own lives, and a summary comprehension quiz (Materials and Methods and Supplement 3). The control intervention was based on materials from emotion-focused therapy (*43*) and was closely matched in terms of length, interactivity, and self-relevant exercise content, although it did not contain reference to cognitive interpretations influencing feelings or include reappraisal activities (e.g., reflection on whether a particular emotional reaction is helpful or not). Completion times for interventions were well-matched across studies (fig. S6, C and D).

#### A cognitive restructuring intervention decreases tendency to attribute negative events to internal (self-related) causes

**Fig. 4.**
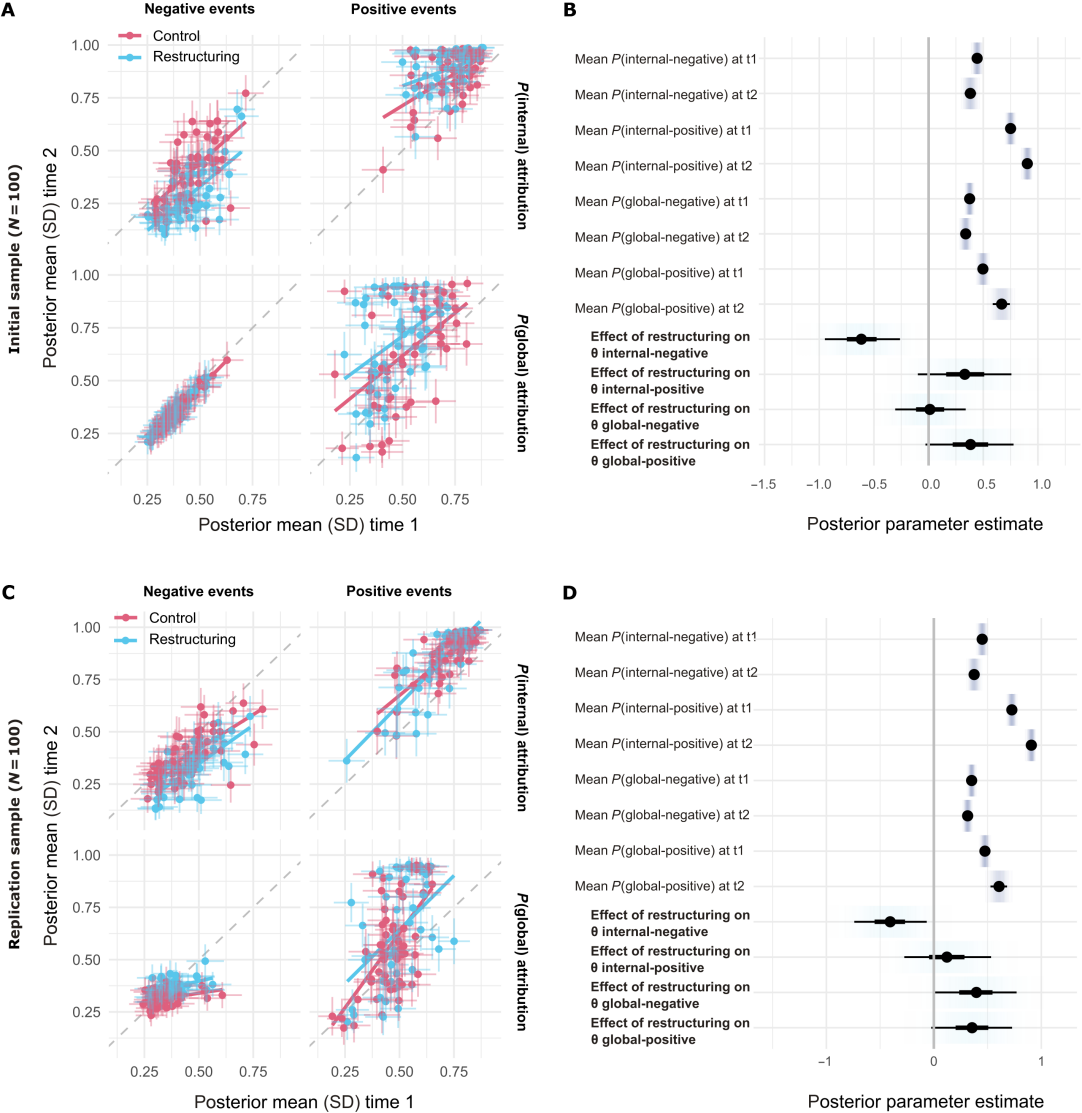
A cognitive restructuring intervention based on cognitive therapy resulted in decreased internal attribution of negative events, compared to a well-matched control condition. (**A**) Posterior mean (and SD) parameter estimates for each participant at time 1 (preintervention) and time 2 (postintervention), by intervention group, in the initial discovery sample (cognitive restructuring intervention, *N* = 49; control intervention, *N* = 51). Parameter estimates plotted here represent the probability of endorsing a given kind of attribution for positive and negative events, which are governed by the latent trait parameters θ). Lines of best fit for mean time 1 versus time 2 estimates for individuals in each group are plotted for illustration purposes. (**B**) Posterior parameter estimates for group means (over all participants/intervention conditions) for each parameter at each time point and the additional effect of intervention in cognitive restructuring group participants at time 2, in the initial discovery sample. Thick inner lines represent 50%, thin outer lines represent 90% CIs, the point estimate is the mean, and shading represents posterior probability density. For visualization purposes, intervention effects (bold text) have been scaled by the square root of the mean posterior variance estimates for parameter values at time 2, making them roughly equivalent to SMDs. (**C**) The same plot as (A), for the independent replication sample (cognitive restructuring, *N* = 44; control, *N* = 56). (**D**) The same plot as (B), for the independent replication sample.

In both initial discovery (*N* = 100) and replication samples (*N =* 100), linear mixed-effects modeling of individual trial data revealed a significant interaction between intervention condition and time point (pre- versus postintervention) on proportionate choice of internal attributions for negative events (*F*_1,6294_ = 10.9, *F*_1,6294_ = 5.0; both *P* < 0.03)—with lower choice of internal attributions for negative events at time 2 in the cognitive restructuring group (Supplementary Results and fig. S6). Analysis via our prespecified Hierarchical Bayesian model revealed that, in both samples, this was due to a decrease in the model parameter describing the latent tendency of individuals to internally attribute negative events following the restructuring intervention (posterior means for group-level effect of the cognitive restructuring intervention at time 2 = −0.56 [90% posterior CI −0.81,−0.32], −0.34 [90% posterior CI −0.56,−0.12]; Fig. 4 and table S2). Mean posterior predictive accuracy of the model for each sample was 0.74 (SD 0.11) and 0.73 (SD 0.11) for internal attributions, and 0.69 (SD 0.11) and 0.68 (SD 0.11) for global attributions. Pseudo-*r*^2^ values were 0.64 and 0.64 for internal attributions and 0.59 and 0.58 for global attributions, respectively.

### Effects of interventions based on different components of cognitive-behavioral therapy on their proposed cognitive mechanisms: Interim summary

In two parallel sets of studies, we found (i) evidence that a goal setting intervention based on principles of behavioral activation therapy reliably reduced sensitivity to required effort levels when choosing between different actions and (ii) that a restructuring intervention based on cognitive therapy reliably reduced a tendency to attribute negative events to self-related or internal causes (an aspect of attributional style thought to contribute to symptoms of low mood).

However, it is not possible to tell on the basis of results so far whether the effects of each intervention were specific to the task administered in each study, or whether each intervention’s effect might “spill over” to other cognitive domains (Fig. 1B).

### Specificity of interventions to proposed cognitive mechanisms

To test whether effects of our interventions were specific to their proposed cognitive mechanisms, we next carried out a study using a 2 × 2 intervention × task crossover design (Fig. 1F). Specifically, participants were separately randomized to task and intervention conditions, to investigate the effects of goal setting versus cognitive restructuring on reward-effort decision-making and cognitive restructuring versus goal setting on causal attribution. Participants were recruited as previously and are described in Table 1.

#### Goal setting but not cognitive restructuring affects effort sensitivity during reward-effort decision-making

**Fig. 5.**
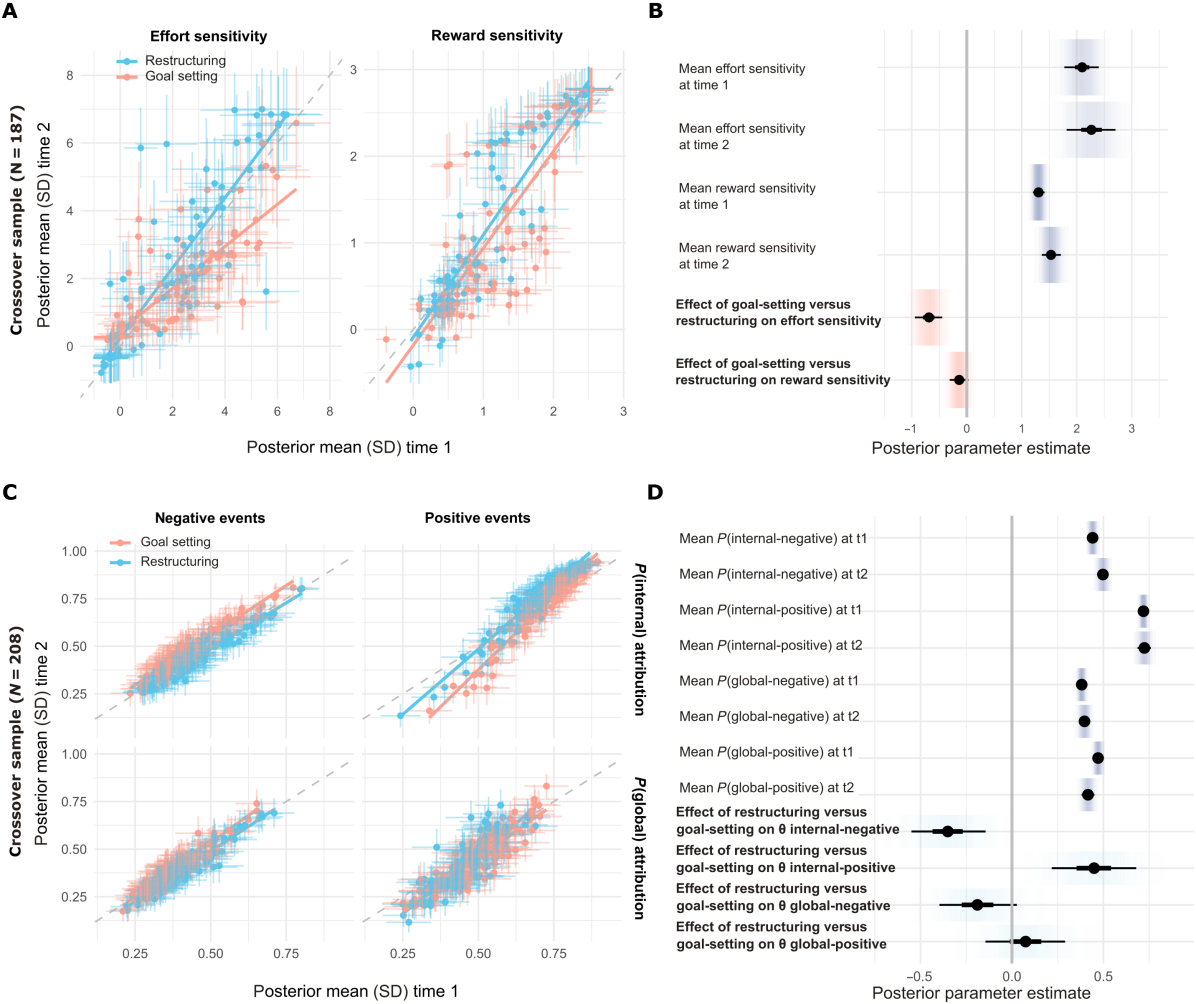
In a crossover design, effects of goal setting and cognitive restructuring interventions were found to be specific to their relevant cognitive mechanisms. (**A**) Posterior mean (and SD) parameter estimates for each participant at time 1 (preintervention) and time 2 (postintervention), by intervention group, for the crossover study participants randomized to the reward-effort decision-making task (goal setting, *N* = 99; cognitive restructuring, *N* = 88). (**B**) Posterior parameter estimates for group means (over all participants) for each parameter at each time point and the additional effect of the goal setting intervention at time 2, in the crossover study participants who completed the reward-effort decision-making task. Compared to restructuring, goal setting reduced effort sensitivity. Thick inner lines represent 50%, thin outer lines represent 90% CIs, the point estimate is the mean, and shading represents posterior probability density. (**C**) The same plot as (A), for the crossover study participants who were randomized to the causal attribution task (cognitive restructuring, *N* = 106; goal setting, *N* = 102). (**D**) The same plot as (B), for the additional effect of the cognitive restructuring intervention at time 2, in the crossover study participants who completed the causal attribution task. Compared to goal setting, restructuring reduced internal attribution of negative events and increased internal attribution of positive events.

For crossover study, participants who were randomized to complete the reward-effort decision-making task (*N* = 197), Hierarchical Bayesian analysis revealed that goal setting but not cognitive restructuring resulted in decreased effort sensitivity during reward-effort decision-making (posterior mean for group-level effect of goal setting versus restructuring = −0.69 [90% CI −0.89,−0.50]; Fig. 5, A and B, and table S3).

#### Cognitive restructuring but not goal setting affects internal attribution of negative events

For crossover study, participants who were randomized to complete the causal attribution task (*N* = 208), Hierarchical Bayesian analysis revealed that the cognitive restructuring but not goal setting intervention resulted in reduced internal attribution of negative events (posterior mean for group-level effect of restructuring versus goal setting on negative events = −0.28 [90% CI −0.40,−0.15]; Fig. 5, C and D, and table S3) Further, in this sample, cognitive restructuring was associated with increased internal attribution of positive events (posterior mean for group-level effect of restructuring versus goal setting on positive events = 0.46 [90% CI 0.27,0.65]).

Of note, under this analysis framework, effects common to both intervention conditions would be expressed as changes in group-level parameter means between time 1 and time 2; however, posterior distributions (90% CIs) for group means were overlapping for all parameters across time points (Fig. 5, B and D). Therefore, data from this study provided not only a further replication of the effects found in the first set of studies but also showed that the effects of each intervention appeared specific to their relevant theoretically informed task and parameter measures.

### Relating magnitude of intervention effects to individual symptom profiles

Last, we conducted an exploratory analysis to determine whether individual differences in psychological symptom profiles might moderate the effects of interventions on our cognitive measures. To increase power, initial discovery and replication samples from the sets of studies described above were first combined for each task. We then sought to determine whether any effects in these combined samples were replicated in the crossover study data (where comparison interventions were less well-matched in terms of, e.g., length and interactivity).

#### Heterogeneity of treatment effects analysis

Across tasks and measures, we found evidence of moderate response variation in terms of change in mean effort sensitivity following the goal setting intervention (point estimate for SD of individual responses = 0.42 [95% CI 0.32,0.51]) and mean tendency to attribute positive events to internal causes following the cognitive restructuring intervention (point estimate for SD of individual responses = 0.40 [95% CI 0.04,0.76]).

#### Joint modeling of task and self-report data

**Fig. 6.**
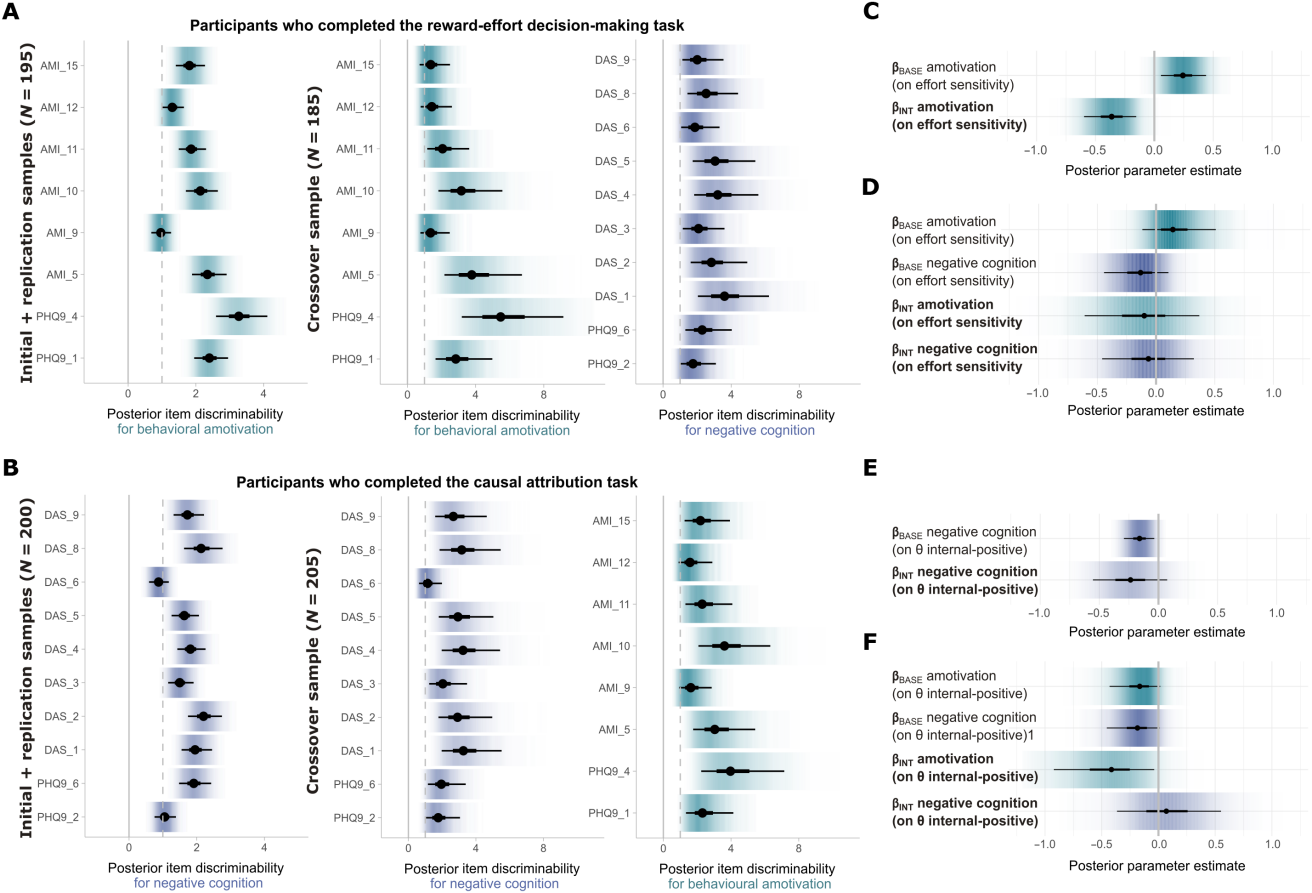
Relationships between psychological symptoms and magnitude of intervention effects, in joint models of behavioral and self-report data. (**A**) Posterior item discriminability estimates for behavioral amotivation in the combined reward-effort decision-making sample (left) and for amotivation and negative cognition in crossover study participants who completed the reward-effort decision-making task (middle and right). AMI*_x*, Apathy Motivation Index behavioral subscale items; PHQ*_x*, Patient Health Questionnaire depression symptom items; DAS*_x*, Dysfunctional Attitude Scale items. (**B**) The same plot as (A), for negative cognition in the combined causal-attribution task samples (left) and negative cognition and behavioral amotivation in crossover study participants who completed the causal-attribution task (middle and right). (**C**) Posterior estimates for the influence of behavioral amotivation on effort sensitivity at baseline (β_BASE_ amotivation) and on the magnitude of the effect of the goal setting intervention (β_INT_ amotivation), in the reward-effort decision-making sample. For visualization purposes, β estimates have been scaled by the ratio of SDs of the predictor (trait estimates) to outcome (mean posterior parameter variance estimates), making them roughly equivalent to standardized regression coefficients. (**D**) The same plot as (C), for crossover study participants who completed the reward-effort decision-making task, including the influence of negative cognition on baseline and intervention-induced changes in effort sensitivity (β_BASE_ negative cognition, β_INT_ negative cognition). (**E**) The same plot as (C), for influence of negative cognition on internal attribution of positive events in the causal-attribution task sample. (**F**) The same plot as (D), for the influence of behavioral amotivation and negative cognition on internal-positive attributions in crossover study participants who completed the causal-attribution task. In all panels, thick inner lines represent 50%, thin outer lines represent 90% CIs, the point estimate is the mean, and shading represents posterior probability density.

To test whether symptom profiles were related to magnitude of either of these responses, individual symptom data were combined into the previously described behavioral task analysis models. Following (*44*), within the joint model, individual item self-report data were analyzed using item response theory (IRT). Specifically, we hypothesized the existence of two latent traits in the symptom data, labeled “behavioral amotivation” (symptoms of anhedonia and behavioral apathy: constructed from Apathy Motivation Index (AMI) behavioral amotivation items and nine-item Patient Health Questionnaire (PHQ9) items indexing anhedonia and lethargic symptoms) and “negative cognition” (negative self-beliefs associated with depressed mood: constructed from Dysfunctional Attitude Scale items and PHQ9 items indexing feelings of hopelessness and failure; see Materials and Methods). These traits were chosen on the basis of proposals that behavioral treatments might be more effective for clinical presentations dominated by the former and cognitive treatments for the latter [e.g., (*15*, *45*)]. Figure 6 (A and B) shows that the pattern of item contributions to each latent trait estimate was relatively stable across samples. Top discriminating items for behavioral amotivation included “little interest or pleasure in doing things” and “feeling tired or having little energy,” and top discriminating items for negative cognition included “if other people know what you are really like, they will think less of you” and “if I don’t set the highest standards for myself, I am likely to end up a second-rate person” (for further details on relationships between traits and relationships to depression symptom severity, see Supplementary Results and fig. S7).

#### Individual differences in the effect of the goal setting intervention on reward-effort decision-making

In the combined goal setting versus control intervention sample (*N* = 195), higher amotivation estimates were associated with both greater effort sensitivity at baseline (posterior parameter estimate for group-level β weight of trait amotivation estimates on time 1 effort sensitivity estimates, β_BASE _= 0.23 [90% CI 0.07,0.40]) and greater magnitude of response to the goal setting intervention (posterior estimate for β weight of amotivation on group-level active intervention effect, β_INT_ = −0.37 [90% CI −0.55,−0.19]; Fig. 6C and table S4). The direction (but not magnitude) of these effects was replicated in the less well-controlled crossover sample, where amotivation and negative cognition estimates could be included in the same model (*N* = 185, β_BASE _= 0.05 [90% CI −0.05,0.16], β_INT_ = −0.02 [90% CI −0.18,0.15]; Fig. 6D and table S4). Evidence that individuals higher in amotivation differed in baseline effort sensitivity and showed greater response to the goal setting intervention was therefore somewhat inconclusive.

#### Individual differences in the effect of the cognitive restructuring intervention on causal attribution

In the combined restructuring versus control intervention sample (*N* = 200), higher negative cognition estimates were associated with a lower tendency to attribute positive events to internal causes at baseline (β_BASE_ = −0.16 [90% CI −0.26,−0.06]) but not magnitude of change in this measure following restructuring (β_INT_ = −0.24 [90% CI −0.48,0.004], Fig. 6E, table S4). In the crossover sample (*N* = 205), there was evidence that both higher amotivation symptoms and higher negative cognition were associated with lower tendency to internally attribute positive events at baseline (β_BASE_ = −0.11 [90% CI −0.21,−0.02], −0.11 [90% CI −0.20,−0.01]). There was again no relationship between negative cognition and change on this measure following the restructuring intervention, but there was evidence of a negative relationship with amotivation (β_INT_ = −0.30 [90% CI −0.50,−0.10]; Fig. 6F and table S4). This suggests that while symptoms of both amotivation and negative cognition are associated with lower baseline self-protective attributional tendencies, only greater amotivation symptoms were associated with response on this measure to an intervention based on cognitive restructuring, with greater amotivation relating to smaller increases in internal-positive attribution tendency.

In summary, we found some evidence for higher amotivation symptoms relating to greater response to a goal setting intervention based on behavioral activation therapy but a smaller response to a restructuring intervention based on cognitive therapy, in terms of change in underlying cognitive mechanisms. However, we caution that these results are very preliminary and will require replication in the future work.

## DISCUSSION

Over the last half century, there have been many calls for research into mechanisms by which existing effective psychotherapy treatments work (*4*, *7*). Large individual patient data meta-analyses have provided some hints of differences in effects between treatments and in different groups of individuals given the similar treatments (*46*–*48*). Recent analyses of large-scale intensively sampled mood data have also shown that symptom clusters representing anhedonia/lethargic symptoms and depressed mood/feelings of worthlessness exhibit different dynamic properties within and between individuals, which may represent different opportunities for intervention (*49*). However, it remains largely unclear which individuals are more likely to benefit from different kinds of treatment, particularly cognitive versus behavioral therapies, and this is an active area of ongoing research (*50*, *51*). Further, a key issue in psychotherapy process research is distinguishing causal relationships from correlates of treatment response (*52*, *53*). This is critical, as only the former are likely to support the longer-term goal of truly effective treatment personalization. Here, we show that using well-validated cognitive measures, in conjunction with experimental designs capable of supporting causal inference, we can test directly whether different proposed mechanisms are affected by interventions based on distinct components of psychological therapies.

We found that a brief goal setting intervention, which included education about the importance of setting achievable goals and salient visual tracking of progress toward goals, reliably led to increased selection of higher-effort/higher-reward actions. Model-based analysis revealed that this was due to a selective reduction in sensitivity to required effort levels (but not sensitivity to potential rewards), when deciding how to act (Fig. 3). Significantly, this change in decision-making was accompanied by an increased sense of achievement for actions and experienced pleasure for rewards, suggesting not only that goal setting decreased subjective weighting of effort but also that the resulting energizing of overall action levels may be sufficient to kick-start a positive reinforcement cycle through which behavioral activation therapy is thought to improve mood (*10*, *33*). This implies that a focus on setting achievable goals (which are gradually increased over time) and active monitoring of completion of activities (e.g., via monitoring forms) may be key active ingredients of behavioral activation therapy. It is not clear from our current results which particular features of our intervention (brief education about achievable goals, precommitment to a specific target, and monitoring of progress toward this target) were most potent in effecting this change, which can be further dissected in future work. It will also be important to test whether the effects identified here generalize from in-game actions and rewards to the kinds of everyday effortful activities and rewards used in a therapeutic context (see below).

We also found that a brief restructuring intervention, which included education about a cognitive model of low mood (“thoughts affect feelings”) and reappraisal practice, reliably reduced a tendency to attribute negative events to internal (self-related) causes while not robustly affecting a tendency to assign events to overly general or global causes (Fig. 4). Both heightened internal and global attributional styles are implicated in depressed mood (*40*, *41*), and we observed associations between both these tendencies and depression symptoms and negative self-beliefs in our samples (Fig. 6 and fig. S8). We note that, in general, participants found the internal-external dimension of choice options easier to parse than the global-specific dimension, which may explain the lack of robust effects on this measure. It is not now clear whether this is a limitation of our task materials or reflects a more general difficulty in understanding this aspect of attributional style, something that can be usefully explored in future work [e.g., (*54*)]. Further, it is an open question whether expression of these kinds of belief is a cause or consequence of low mood (*55*, *56*). Here, we provide initial evidence that an intervention based on cognitive restructuring directly affects attributional choice in a realistic scenario-based task that is robust to sociodemographic differences. In the future, this kind of measure may enable more precise and reliable tracking of changes in causal attribution over the course of treatment and determination of whether or not this predates symptom change.

A critical aspect of our results is our demonstration that changes in theoretically derived cognitive measures were specific to relevant interventions (Fig. 5). This is a vital step toward an eventual goal of providing more targeted or personalized psychotherapy treatment, as if different cognitive processes are affected by multiple treatment components to the same extent, and then it would render it hard to leverage differential administration or dosage of components to address relative deficits (or capitalize on relative strengths) on the basis of measurements of these processes (*53*). Last, we presented preliminary evidence that symptoms of behavioral amotivation (anhedonia and lethargy) may relate to greater responses to goal setting and lesser responses to cognitive restructuring (Fig. 6). This accords with theoretical notions that behavioral treatments may be preferable for clinical presentations dominated by this kind of symptom profile (*15*, *45*), although these findings should be interpreted with caution as they did not replicate fully across samples.

The major limitation of these initial proof-of-concept results is that they concern the effects of custom brief interventions based on components of psychological therapies, as opposed to modules of real, proven to be effective, cognitive, and behavioral treatments. Extension of our findings to this context is therefore a critical next step in constructing a chain of evidence that unpacks the mechanisms by which real-world therapies work. Such a translation would also enable us to complete a vital link that relates change in cognitive mechanisms to parallel change in psychological symptoms following treatment completion—as, of note, because of the single session experimental designs used here, we were not able to measure changes in self-reported symptoms. To facilitate fast and high-throughput initial testing of these questions and maximize our chances of detecting effects, all studies took place in a single experimental session, and the interventions were relatively tailored toward our task-based mechanism measures. This means that the relatively robust effect sizes identified here are likely to be somewhat inflated by temporal proximity of interventions to measures and potentially also by demand effects. Specifically, we note that, for the reward-effort decision-making studies, to create an analog of goal-progress monitoring during behavioral activation therapy, the goal setting intervention included changes to the second task (goal setting and visual tracking of goal progress) that were not present in the control condition (Supplement 2). For the causal attribution studies, there was also greater congruence between the intervention content and task response format for the cognitive restructuring compared to the control intervention (Supplement 3). In both cases, the contextually specific nature of these interventions limits the ecological validity of our findings with respect to authentic psychological treatments, where participants must apply these techniques in their real lives. It remains possible that there are too many differences between our toy interventions and actual psychotherapies (even highly controlled digitally delivered content) for our results to hold. However, we believe that initial evidence of replicable effects of therapy-derived interventions on theory-based mechanisms and, in particular, evidence of specific effects of these interventions represent a foundational step before embedding such tests in resource-intensive contexts, such as clinical trials or treatment programs (*32*).

An important lesson learned over the course of these studies is that the development of “good” measures of cognitive processes fundamentally involves the management of various competing trade-off factors (*26*). Specifically, increasing user engagement via gamification strategies (e.g., our reward-effort task) may involve a trade-off between noisiness of data and face or construct validity. Conversely, measures with increased face validity (e.g., our scenario-based causal attribution measure) may involve a different degree of insight than more behavioral tasks, where individual differences in interpretation or understanding of the state space may be a source of noise. Optimal points for these trade-offs may be hard to judge on the basis of isolated quantitative measurements (such as test-retest reliability) and better understood in the context of qualitative input from future end-users (*57*).

In conclusion, digital therapies can help reduce the treatment gap in mental health service provision (*58*, *59*), in particular for underserved populations (*60*). However, increasing user engagement is likely to be key for greater uptake of digital therapeutics (*27*, *61*). Promising targets for increasing engagement with such services include increasing value to end users (e.g., providing knowledge back) and evidence of personalization of content (*62*). We argue that greater knowledge about the mechanisms via which established psychological treatments work is an important step toward achieving these goals (*63*).

## MATERIALS AND METHODS

### Ethical approval

All participants gave written informed consent, and all studies were approved by the University College London Research Ethics Committee (project ID 21029/001).

### Participants

For all studies, participants were recruited from an online research participation platform (Prolific) and were required to be based in the UK, 18 to 65 years old, and fluent in English. For each study, recruitment continued until the target number of participants had completed the full experiment. Because of the single-session nature of our design, drop-out rates across studies were low (3% following initial consent and instructions; see Supplementary Methods).

### General methods

All analyses were carried out in R version 4.1.2 (The R Foundation for Statistical Computing, 2021), using RStudio version 2022.02.0 (RStudio, PBC, 2022).

#### Hierarchical Bayesian modeling

Model evaluation and fit procedures were carried out according to Bayesian workflow recommendations (*24*, *64*), with results of Bayesian analyses reported in accordance with recent guidelines (*65*). Model parameters were estimated using Markov-Chain Monte Carlo (MCMC) sampling as implemented in Stan 2.21.0 (*66*), using RStan 2.21.3 (Stan Development Team, 2021). MCMC chains were initiated with random starting values, and posterior distributions were formed using four chains of 2000 iterations, with 1000 discarded warm-up samples (i.e., 4000 kept iterations per model). Convergence of sampling chains was assessed via inspection of trace plots and Gelman-Rubin ( $\hat{R}$ ) statistics for each parameter (*67*). Assessment for sampling difficulties and parameter collinearity was via inspection of bivariate marginal posterior distributions between pairs of parameters. All models used generic weakly informative priors (see Supplementary Methods).

For the main analyses reported here, two model-agnostic “goodness-of-fit” measures are reported. Posterior predictive accuracy was calculated as the match between replicated choice data generated stochastically from posterior parameter estimates and task trial arrays and the observed data from each participant (means and SDs across participants are reported). Pseudo-*r*^2^ statistics, which reflect the amount of variance explained by the model relative to a model of pure chance, were calculated as 1 − *L*/*C*, where *L* is the summed log likelihood over participants, and *C* is the chance likelihood of observing responses [for two choice options, *log*(0.5)*t*] (*68*).

For experimental effects of interest (e.g., the group-level effect of receiving the active versus control intervention on parameter estimates at time 2), parameters were assessed using 90% CIs, with a 90% CI excluding zero interpreted as representing evidence for a meaningful contribution to posterior parameter estimates. Although this choice of threshold is somewhat arbitrary, it follows conventions in the literature and recommendations of use of a <95% CI for sample sizes less than 10,000 (*69*). Distributions of posterior parameter estimates and CIs were visualized using the R packages bayesplot (*70*) and tidybayes (*71*).

#### SBC analysis

SBC analysis was used to validate our modeling and inference procedures for both tasks and sets of measures (*23*). Briefly, this involves generating draws from the prior predictive distribution of a generative model (creating *N* simulated datasets) and then fitting the model to each simulated dataset and obtaining *D* independent draws from the model posterior. For each parameter of interest, the rank of the simulated value within the posterior draws is then calculated. If the data generation and inference procedure works as expected, then the resulting ranks should be uniformly distributed across [0, *D*] (*72*). Here, we generated *N* = 1000 datasets based on independent draws from the prior distributions of each parameter, which were specified based on the empirical posterior estimates of parameter distributions observed in pilot data. We then took *D* = 2000 posterior draws (after discarding 1000 warm-up samples), across two sampling chains. Graphical summaries of SBC results were generated using the R package SBC (*73*).

#### Test-retest reliability analysis

Recent discussions highlight adequate test-retest reliability as a prerequisite for detection of true individual differences in a measure (*21*, *22*, *26*, *74*). Here, we estimated test-retest reliability using the approach described in (*20*, *21*). Specifically, data from two time points (repeat test administration in the same sample of participants) were fit using a single hierarchical model, with separate group means for each parameter at each time point and individual parameter estimates at each time point assumed to be drawn from a multivariate normal distribution and a uniform prior over [−1,1] on correlation of individual values across time points (see Eqs. 3 and 10). Posterior *R* values for correlation of individual parameter estimates across time points are then reported as an estimate of test-retest reliability, which sufficiently takes into account both relatedness of different measurements and measurement error (precision) of individual estimates.

#### Self-reported demographic and clinical information

At the end of each study, participants completed a set of brief self-report measures to provide information about their recent experience of mental health symptoms and other relevant sociodemographic information. Symptoms of low mood were measured using The PHQ9 (*75*). We also included the three-item Social Phobia Inventory (miniSPIN), a brief measure of social anxiety symptoms (*76*), given our previous observations that social anxiety is relatively elevated in Prolific samples. The AMI, which measures apathy and amotivation across behavioral, social, and emotional domains (*77*), was included for reward-effort decision-making samples, given the hypothesis that behavioral activation therapy may be particularly effective for individuals with disrupted reward or effort processing (*9*, *45*). The DAS, a measure of negative self-beliefs observed in some depressed people (*78*), was included for causal attribution task samples, as it has previously been shown to be sensitive to cognitive treatment of low mood (*55*).

The demographic measure included questions about participant gender identity, age, neurodivergence (defined as a term for when someone processes or learns information in a different way to that which is considered ‘typical’: common examples include autism and attention-deficit/hyperactivity disorder), previous treatment for a mental health problem, disability across World Health Organization Disability Assessment 2.0 domains of functioning (*79*), and financial, housing, and employment status [given these factors have previously been shown to relate to treatment outcomes for depression; (*80*)]. All self-report batteries included two infrequency items (in which some responses are logically invalid or highly improbable), to detect potential inattentive responding (*81*). Participants were required to provide correct responses to both items to be included in analyses including self-report data.

### Reward-effort decision-making studies

#### Reward-effort decision-making task

Code for implementing the version of the task described here and a link to a demo version of the game is available at https://github.com/agnesnorbury/cognitive-mechanisms-psychotherapy. The task was coded in javascript using phaser 3.23.0, a framework for creating HTML5 games for desktop and mobile devices (Photon Storm, 2020).

Participants were informed that they were traveling through a strange land, covered in rivers and streams. At regular points along their journey, they would be required to power up their magic umbrella, to fly across the water. At each crossing point, they could choose between different routes. Different routes would allow them to collect different numbers of coins (with total coins converted into a cash bonus at the end of the study) but required different amounts of effort to cross. For each route, they would have to press or click quickly an on-screen “power” button, until they reached the required effort level to cross. Effort levels were presented as percentages of maximal power, which (unknown to participants) was individually calibrated at the start of the study during a series of practice trials, designed to elicit maximal possible effort levels (press rates) during the time limit (10 s). To avoid “gaming” of practice trials, a minimal effort level was also applied.

The main task consisted of 44 choice trials divided into four blocks. This included two catch (nondominated choice) trials, where the highest reward was offered for the lowest effort level. To be included in the analysis, participants were required to select the “correct” answer on at least one of the catch trials. At the end of each block, participants rated their sense of achievement upon successful effort exertion, sense of pleasure upon gaining rewards, and overall boredom levels, using an interactive slider.

#### Interventions

The full content of the goal setting and control interventions for the reward-effort decision-making studies is available in Supplement 2. For the goal setting condition, the intervention consisted of a brief information about the importance of setting achievable goals, a multiple choice comprehension check and a modified version of the task (at time 2 only) where participants were invited to set a goal for each block, progress toward which was tracked visually over trials. The control intervention consisted of brief information about different kinds of online games, a multiple choice comprehension check, and a modified version of the task (at time 2) where participants were asked to rate how much they liked different kinds of games at the start of each block, but there was no goal setting or visual tracking.

#### Power analysis and participant exclusion

In a pilot dataset (total *N* = 20; *N* = 10 goal setting and *N* = 10 control intervention randomized), we observed a large effect of intervention condition on choice of higher effort/higher reward choice options (intervention group × time interaction in the mixed-effects model described below, *F*_1,1737_ = 4.12, *P* = 0.04; Cohen’s *d* for effect of intervention group on change between time 1 and time 2 = 0.97). Since we observed a large effect and high correlation across repeated-measures in this dataset (*R* = 0.90 for mean proportion of higher effort/higher reward choices across time points), we conducted a conservative power analysis, assuming that these quantities might be reduced in future samples. Analysis using G*Power 3.1 (*82*) determined that we could replicate an effect half this size (*d* = 0.48) in *N* = 48 participants with 95% power [repeated-measures analysis of variance (ANOVA) between-within interaction with two groups, two measures per group, assuming 0.6 correlation across repeated-measures and alpha = 0.05]. Given the relative ease of online data collection, subsequent studies were super-powered to *N* = 100 per sample.

The initial (discovery) sample therefore consisted of *N* = 100 participants (*N* = 0 excluded from behavioral data analysis according to the rule described above) and the replication sample consisted of *N* = 102 participants (*N* = 0 excluded). A total of *N* = 5 participants were excluded from analyses that included self-report data, for providing improbable answers to infrequency (catch) items.

#### Initial statistical analysis

Preliminary statistical analysis of choice behavior was a via mixed-effects logistic regression model, as implemented in lme4 (*83*). Individual choices were categorized as to whether or not the higher effort/higher reward option was chosen on each trial and modeled as

choice∼interventionCondition*taskNo+trialNo+(1∣subID) (1)

Where appropriate, pairwise differences were assessed using follow-up *t* tests using the Tukey adjustment for multiple comparisons, as implemented in the R package emmeans.

#### Hierarchical Bayesian analysis

The most parsimonious model of choice behavior, taking into account parameter recovery from the optimized task design and model comparison results in pilot datasets (see above), was a simple linear model with two free participant-level parameters representing reward and effort sensitivity

$$V_{i,s,t}={\text{rewSens}}_{s}*{\text{reward}}_{i,s,t}-{\text{effSens}}_{s}*\text{effor}t_{i,s,t}$$ (2)

where *V* is the value of each choice option (*i*) for each trial (*t*) and session (*s*; time 1 or time 2), based on the reward offered (reward), required effort (effort) and participant reward (rewSens) and effort sensitivity (effSens) parameters for that time point. As described above, we assumed that task parameters across time points (pre- versus postintervention) were drawn from multivariate normal distributions.

$$\begin{array}{c} \left[\begin{array}{c} {\text{rewSens}}_{1}\\ {\text{rewSens}}_{2} \end{array}]∼\text{MVNormal}([\begin{array}{c} {\text{rewSens}}_{\mu ,1}\\ {\text{rewSens}}_{\mu ,2} \end{array}],\sigma_{\text{rewSens}}\right)\\ \left[\begin{array}{c} {\text{effSens}}_{1}\\ {\text{effSens}}_{2} \end{array}]∼\text{MVNormal}([\begin{array}{c} {\text{effSens}}_{\mu ,1}\\ {\text{effSens}}_{\mu ,2} \end{array}],\sigma_{\text{effSens}}\right) \end{array}$$ (3)

where effSens_μ,*s*_ and rewSens_μ,*s*_ are the group-level means for each parameter and time point, and σ is the covariance between individual-level parameters across time points {prior correlation between time points was set to be uniform over [−1,1], using an LKJ(1) prior}. Choice values (*V*_*i*,*s*,*t*_) were assumed to map onto observed choice data (*y*) using a simple Bernoulli likelihood function

$$y_{p,s,t}∼\text{Bern}\left[\text{logit}\left(V_{2,s,t}-V_{1,s,t}\right)\right]$$ (4)

Participant-level parameter estimates were constructed using noncentered reparameterization to separate the hierarchical parameters and lower-level parameters in the prior (*84*). For each parameter (e.g., ϕ) and time point *s*, participant-level estimates (ϕ_*p*,*s*_) were constructed from a group mean (ϕ_μ,*s*_) and an individual offset ( ${\tilde{\phi }}_{p,s}$ ). The between-subjects effects of intervention group were then modeled as

$$\begin{array}{c} \begin{array}{c} \phi_{p,1}=\phi_{\mu ,1}+{\tilde{\phi }}_{p,1}\\ \phi_{p,2}=\left\{\begin{array}{cc} \phi_{\mu ,2}+{\tilde{\phi }}_{p,2}+\phi_{\text{INT}}, & \text{if active intervention}\\ \phi_{\mu ,2}+{\tilde{\phi }}_{p,2}, & \text{otherwise} \end{array}\right. \end{array} \end{array}$$ (5)

where ϕ_INT_ is a group-level parameter describing potential effects of allocation to the active intervention on parameter estimates at time 2. For all models, the priors for effects of active intervention on parameter estimates were centered on 0 [ϕ_INT_ ∼ *N*(0,1)]. For full details of parameter constraints and model priors see Supplementary Methods.

### Causal attribution studies

#### Causal attribution task

Code for implementing the task and a link to a demo version is available at https://github.com/agnesnorbury/cognitive-mechanisms-psychotherapy. The task was coded in javascript using the jsPsych library, version 7.2.1 (*85*).

Participants were instructed that during the task they would be asked to imagine themselves in various everyday situations. For each situation, they were asked to picture the situation described as clearly as they could (“as if the events were happening to them right now”) and then choose which of several possible explanations listed below they thought most likely. Specifically, participants were informed that, although events can have multiple different causes, they should choose the explanation they thought closest to the main reason the event happened, if it had actually happened to them.

Participants were presented with 32 event scenarios (16 positive and 16 negative events, randomly interleaved), divided into two blocks. Event scenarios were based on analysis of the previous literature (*86*–*88*) and drawn from interpersonal (e.g., someone you are close to tells you that they admire you), professional/academic (“You and your friends do a general knowledge quiz and you get the lowest score”), and general life-functioning domains (“You fix something around the house that you have been meaning to get done for a while”). For each event, participants were asked to choose between four response options that varied orthogonally in terms of internal-external and global-specific explanation types, derived from examples provided in (*38*). For example, for the event “You find out that someone you consider a friend has talked about you negatively behind your back,” possible explanations were “Deep down, my friends don’t really like me” (internal-global), “I probably did something recently to annoy them” (internal-specific), “Everyone has bad things said about them sometimes” (external-global), and “My friend was probably just in a bad mood and letting off steam” (external-specific). We chose to focus on these two attribution dimensions as these have been most reliably linked in the past to low mood symptoms (*40*, *41*). Full details of scenarios by event type (valence; interpersonal/not), category (close relationship/friends/contemporaries/colleagues; general performance-professional/general performance-academic; general life functioning), and possible attributions are available at https://github.com/agnesnorbury/cognitive-mechanisms-psychotherapy.

Event scenarios for the final task version were chosen on the basis of analysis of a fuller (128) item set during pilot testing. Specifically, responses to the full item set were collected in *N* = 100 participants, and the data modeled using Item-Response Theory. Subsets of items with the highest discriminability parameters for latent tendencies to make internal and global attributions for positive and negative events were then derived, ensuring final item sets with balanced positive/negative event frequencies and that all items contribute meaningfully to trait parameter estimates (posterior mean discriminability > 1). We further conducted internal consistency, split-half analysis of attribution type counts, and test-retest reliability analysis of our trait parameter estimates, to ensure consistent responding across event types and over time (see Results and Supplementary Results). Given the novelty of this task, we also sought to validate the derived measures by relating them to negative self-beliefs as measured by the DAS and current levels of depression and social anxiety symptoms (Supplementary Results). Last, given the likelihood that responses to realistic social/professional scenarios might be influenced by individual and social factors, we examined whether trait parameter estimates varied substantially according to various relevant measures (e.g., age, functional disability, and minoritized status; see Supplementary Results).

The final item set did not include catch trials, but we applied the following exclusion rules to participants’ choice data: median response time was required to be >2 s, and proportionate choice of each response option position (e.g., top-left) was required to be <75% (participants were aware of these rules before completing the task and informed that their compensation for taking part in the study may depend on these rules; different response options were displayed randomly in each position on each trial).

#### Interventions

Taking inspiration from materials described in (*89*), both restructuring and control interventions were in the form of a series of interactive worksheets, requiring participants to select answers from multiple potential options during worked examples, and provide input based on recent positive and negative experiences from their own lives. The full content of the cognitive restructuring and control interventions (described in the main text) is available in Supplement 3. For these studies, all participants completed the same tasks at time 1 and time 2 (i.e., the two equivalent versions of the causal attribution task; see Supplementary Results).

#### Power analysis and participant exclusion

In a pilot dataset (total *N* = 20; *N* = 12 restructuring and *N* = 8 control intervention randomized), we observed a moderate effect of intervention condition on choice of internal attributions for negative events (intervention group × valence × time interaction in the mixed-effects model described below, *F*_1,1254_ = 3.38, *P* = 0.07; Cohen’s *d* for effect of intervention group on change between time 1 and time 2 = 0.47). Power analysis using G*Power 3.1 determined that we could replicate an effect of this size in *N* = 72 participants with 95% power (repeated-measures ANOVA between-within interaction with two groups, two measures per group, assuming observed correlation of 0.43 cross repeated-measures and alpha = 0.05). Given the relative ease of online data collection, subsequent studies were super-powered to *N* = 100 per sample.

The initial (discovery) sample therefore consisted of *N* = 100 participants and the replication sample of *N* = 100 participants (0 were excluded from either sample based on task data according to the above criteria). Across these samples, no participants additionally were excluded from analyses including self-report data.

#### Initial statistical analysis

Preliminary statistical analysis of choice behavior was via mixed-effects logistic regression models. Individual choices on each trial were categorized according to whether an internal (versus external) and global (versus specific) attribution was selected, and the two orthogonal choice dimensions were separately modeled as

$$\begin{array}{c} {\text{choice}}_{\text{internal}}∼\text{interventionCondition}*\\ \text{itemValence}*\text{taskNo}+\left(1|\text{subID}\right) \end{array}$$ (6)

$$\begin{array}{c} {\text{choice}}_{\text{global}}∼\text{interventionCondition}*\\ \text{itemValence}*\text{taskNo}+\left(1|\text{subID}\right) \end{array}$$ (7)

#### Hierarchical Bayesian analysis

For analysis of causal attribution task data, we used a simple model based on single-parameter IRT model to infer parameters governing a tendency to make internal and global attributions, based on a nonlinear analysis of the pattern of responses across trials. Given evidence of valence-related asymmetry in attribution tendencies in both our data and the wider literature (see Supplementary Results and the main text), separate parameters were used to describe internal and global attribution tendencies for positive and negative events. Specifically, participants’ choices on each trial were coded along two dimensions, according to whether an internal (versus external) or global (versus specific) response option was chosen (y_internal and y_global, respectively), with the resulting data analyzed within a single hierarchical model with four free participant-level parameters

$$\begin{array}{c} y\_\text{interna}l_{p,s,v}∼\text{Bern}\left(\theta_{\text{internal},p,s,v}\right)\\ y\_\text{globa}l_{p,s,v}∼\text{Bern}\left(\theta_{\text{global},p,s,v}\right) \end{array}$$ (8)

where θ_internal,*p*,*s*,*v*_ and θ_global,*p*,*s*,*v*_ represent the latent traits governing a participant (*p*)’s tendency to make an internal or global attribution at that time point or session (*s*), separately for positively and negatively valenced (*v*) event scenarios. We chose this simple model as it maps intuitively onto concepts from attributional style theory (*38*), on evidence that it accounted well for participants’ choices in pilot data, and on the basis that final task items were chosen based on a more complex 2PL IRT analysis of a larger item set, to ensure good discriminability for our traits of interest (see Supplementary Results).

Given pilot data showing correlations between individuals’ tendencies to make global and internal attributions for positive and negative events (Supplementary Results and fig. S8) and to allow maximum information to contribute to individual parameter estimates, we also assumed that individual tendencies to make internal and global attributions for each type of event were drawn from a multivariate normal distribution (allowing for direct estimation of covariance between attribution types within each session)

$$\begin{array}{c} \left[\begin{array}{c} \theta_{\text{internal},1,\text{neg}}\\ \theta_{\text{global},1,\text{neg}}\\ \theta_{\text{internal},2,\text{neg}}\\ \theta_{\text{global},2,\text{neg}} \end{array}]∼\text{MVNormal}([\begin{array}{c} \theta_{\text{internal},\mu ,1,\text{neg}}\\ \theta_{\text{global},\mu ,1,\text{neg}}\\ \theta_{\text{internal},\mu ,2,\text{neg}}\\ \theta_{\text{global},\mu ,2,\text{neg}} \end{array}],\sigma_{\theta ,\text{neg}}\right)\\ \left[\begin{array}{c} \theta_{\text{internal},1,\text{pos}}\\ \theta_{\text{global},1,\text{pos}}\\ \theta_{\text{internal},2,\text{pos}}\\ \theta_{\text{global},2,\text{pos}} \end{array}]∼\text{MVNormal}([\begin{array}{c} \theta_{\text{internal},\mu ,1,\text{pos}}\\ \theta_{\text{global},\mu ,1,\text{pos}}\\ \theta_{\text{internal},\mu ,2,\text{pos}}\\ \theta_{\text{global},\mu ,2,\text{pos}} \end{array}],\sigma_{\theta ,\text{pos}}\right) \end{array}$$ (9)

where θ_internal,μ,*s*,*v*_ and θ_internal,μ,*s*,*v*_ are the group-level means for each parameter and time point [modeled separately for positive (pos) and negative (neg) events], and σ is the covariance between individual-level parameters across attribution types and time points. For full descriptions of parameter constrains and model priors, see Supplementary Methods.

Effects of belonging to the active intervention condition on parameter estimates at time 2 were modeled as described in Eq. 5.

### Crossover study

For the crossover study, participants were randomly assigned to experimental conditions in a 2*2 factorial design of task (reward-effort decision-making or causal attribution) and intervention (goal setting or cognitive restructuring) condition. Tasks and intervention materials were as described previously. For participants randomized to complete the reward-effort decision-making task, the time 2 (postintervention task) was the modified goal setting version for participants allocated to the goal setting intervention and the unmodified (baseline) version for participants allocated to the cognitive restructuring condition.

Analysis was via the same hierarchical models of each task as described above, with the effect of active intervention at time 2 now representing the effect of allocation to the goal setting versus cognitive restructuring intervention on task measures rather than either intervention alone versus a well-matched control.

#### Participant exclusion

*N* = 400 total participants were recruited for the crossover study. *N* = 192 were randomized to complete the reward-effort decision-making task, with *N* = 5 excluded from behavior-only analyses on the basis of catch trial performance. *N* = 208 were randomized to the causal attribution task, with no participants excluded from behavioral analyses. A further *N* = 5 participants were excluded from analyses that included self-report data, on the basis of response to infrequency items.

### Heterogeneity of treatment effects analysis

Before examining individual differences related to magnitude of intervention effects, we first sought to determine whether we had evidence across samples of significant individual differences in responses to active compared to control interventions (*90*, *91*). This analysis involves comparing SDs of change scores in the active and control groups, to assess evidence for greater variance in outcomes in the active intervention group (since we assume control arm change score variance represents effects of individual variability over time and measurement error).

Change scores were defined as differences in mean posterior parameter estimate between time points, and change scores in each arm were standardized by the SD of baseline (pretreatment) posterior means. The SDs of individual responses to the active treatment were then calculated as $SD_{\text{IR}}=\sqrt{S{D_{\text{Act}}}^{2}-S{D_{\text{Con}}}^{2}}$ ; where SD_Act_ and SD_Con_ are the standardized SDs of the change scores in the active and control groups, respectively. Confidence limits for SD_IR_ were obtained by assuming its sampling variance is normally distributed, $S{D_{\text{IR}}}_{\text{se}}=\sqrt{2*(S{D_{\text{Act}}}^{4}/DF_{\text{Act}}+S{D_{\text{Con}}}^{4}/DF_{\text{Con}})}$ , such that the 95% CI was calculated as SD_IR_ ± 1.96*SD_IRse_. DF_Act_ and DF_Con_ are the degrees of freedom of the SDs in the two groups (*N–1*). Where standardized SDs are used, 0.1, 0.3, and 0.6, represent thresholds for small, moderate, and large individual response effects.

### Joint modeling of self-report and task data

#### Self-reported symptom data model

Behavioral amotivation trait estimates were constructed from the six AMI behavioral amotivation subscale items plus the PHQ9 items little interest or pleasure in doing things and feeling tired or having little energy. Negative cognition estimates were constructed from the eight DAS short-form items plus the PHQ9 items “feeling down, depressed, or hopeless” and “feeling bad about yourself or that you are a failure” (Fig. 5, A and B).

To construct individual trait estimates, self-report data were analyzed via a graded response model (GRM) (*92*)–a form of IRT model that was developed to make use of ordinal responses such as ordered Likert scales (essentially, an ordered logistic extension of the model described in eq. S1). Given our relatively limited *N* (∼200 per sample), we allowed items to contribute to their hypothetical latent trait only (i.e., we fit two parallel unidimensional GRMs rather than a more complex multidimensional GRM). This process yields approximately normally distributed latent trait estimates.

#### Combining self-report and task behavior data

Joint modeling allows maximum use of participant-level data while retaining information about uncertainty or precision of each kind of measurement (*44*, *93*–*95*). For the joint model, individual estimates for trait amotivation (θ*_A_*) and/or trait negative cognition (θ*_N_*; constructed as above) were allowed to influence the effect of intervention on time 2 parameter estimates (ϕ_INT_) found to show evidence of heterogeneous individual responses via the inclusion of additional β weight parameters [β_INT_; see (*94*, *95*)] for previous examples of this approach). These β weights can be interpreted similarly as in a standard regression model, with the group-level intervention effect (ϕ_INT_) now representing the intercept (see below).

To account for potential regression-to-the-mean effects caused by baseline associations between task performance and self-reported clinical symptoms (see, e.g., fig. S8), joint models also included β weights for the same parameter estimate at time 1 (β_BASE_)

$$\begin{array}{c} \begin{array}{c} \phi_{p,1}=\phi_{\mu ,1}+{\tilde{\phi }}_{p,1}+\beta_{\text{BASE}}\theta_{A/N}\\ \phi_{p,2}=\left\{\begin{array}{cc} \phi_{\mu ,2}+{\tilde{\phi }}_{p,2}+\phi_{\text{INT}}+\beta_{\text{INT}}\theta_{A/N}, & \text{if active intervention}\\ \phi_{\mu ,2}+{\tilde{\phi }}_{p,2}, & \text{otherwise} \end{array}\right. \end{array} \end{array}$$ (10)

Posterior estimates for β weights with a 90% CI that excluded zero were taken as evidence that the trait estimates were meaningfully related to the effect of interest.

## Supplementary Materials

### This PDF file includes:

Supplementary Methods Supplementary Results Figs. S1 to S8 Tables S1 to S4 Supplement 1 to 3 References
