# Supplementary material for: Different components of cognitive-behavioral therapy affect specific cognitive mechanisms
Source: Sci Adv. 2024 Mar 27;10(13):eadk3222. doi: 10.1126/sciadv.adk3222 (PMC10971416; doi:10.1126/sciadv.adk3222)
Supplement: Supplementary file 1 — Supplementary Methods Supplementary Results Figs. S1 to S8 Tables S1 to S4 Supplement 1 to 3 References [file sciadv.adk3222_sm.pdf]

Science Advances
AAAS

Supplementary Materials for

Different components of cognitive-behavioral therapy affect specific cognitive mechanisms

Agnes Norbury *et al.*

Corresponding author: Agnes Norbury, agnes.norbury@ucl.ac.uk

Sci. Adv. **10**, eadk3222 (2024)
DOI: 10.1126/sciadv.adk3222

This PDF file includes:

Supplementary Methods
Supplementary Results
Figs. S1 to S8
Tables S1 to S4
Supplement 1 to 3
References

Supplementary Methods

Handling of participant drop-out during data collection

For all studies, participants were recruited via the Prolific platform. On this platform, study participants are able to exit or drop-out of the study at any point by 'returning' the study. Recruitment automatically continues until the predetermined *N* participants have completed the full study. Data for all participants who completed the study were then submitted to further analysis (for information about study-specific exclusion criteria, see main text).

Across all studies, return or drop-out rates were low. For the reward-effort decision-making studies, 13% of participants who initially accepted the study returned the assignment - of which the majority (9%) did not complete consent, 6 participants (3%) dropped out during the initial instructions and practice trials, and 2 participants (¡1%) dropped out later during the study. For the causal attribution task studies, 10% of initial accepters returned the assignment: 3% of participants did not complete consent, 9 participants (4%) dropped out during initial instructions or first few trials into the first task, and 6 (3%) dropped out later during the study. For the crossover study, 8% returned the assignment: 5% didn't complete consent, 7 participants (2%) dropped out during initial instructions or first few trials of the first task, and 3 participants (¡1%) dropped out later during the study.

Hierarchical Bayesian modelling of reward-effort decision-making task data

Priors for group-level parameter means were specified using standard normal distributions, $\phi_{\mu,s} \sim N(0,1)$. Priors for group-level parameter standard deviations were specified as $\phi_{\sigma,s} \sim Cauchy(0,1)$. Priors for individual participant deviations from group-level parameter estimates (effort and reward sensitivity) were also specified using standard normal distributions ($\tilde{\phi}_{p,s} \sim N(0,1)$). The prior over the correlation matrix relating parameter estimates across sessions was set to be uniform over $[-1,1]$ using an $LKJ(1)$ prior.

The priors for group-level effects of intervention on parameter estimates at time 2 (ϕ_{INT}), and group-level beta weights governing influence of latent traits on effects of interest (β_{INT}, β_{BASE}), were also specified as standard normal distributions (i.e., centred on zero).

Individual parameter estimates for effort and reward sensitivity were constrained to be in the range $[-1,9]$ and $[-1,3]$, respectively, on the basis of empirical posterior distributions observed in pilot data and values considered to observable based on the range of effort and reward values in our test trial array (this explains the plateauing of inferred values outside this range in simulation-based calibration analysis; Fig. 2A).

Hierarchical Bayesian modelling of causal attribution task data

Priors for group-level parameter means were specified using standard normal distributions, $\phi_{\mu,s} \sim N(0,1)$. Priors for group-level parameter standard deviations were specified as $\phi_{\sigma,s} \sim cauchy(0,1)$. Priors for individual participant deviations from group-level parameter estimates ($\theta_{internal,p,s,neg}$, $\theta_{internal,p,s,pos}$, $\theta_{global,p,s,neg}$, $\theta_{global,p,s,pos}$) were also specified using standard normal distributions ($\tilde{\phi}_{p,s} \sim N(0,1)$). The prior over the correlation matrix relating parameter estimates across sessions was set to be uniform over $[-1,1]$ using an $LKJ(1)$ prior.

The priors for group-level effects of intervention on parameter estimates at time 2 (ϕ_{INT}), and group-level beta weights governing influence of latent traits on effects of interest (β_{INT}, β_{BASE}), were also specified as standard normal distributions (i.e., centred on zero).

Individual parameter estimates for latent traits governing tendency to attribute positive and negative events to internal and global causes were unconstrained but passed to the Bernoulli observation function (Eq. 8) using an inverse logit transform, scaling probability of endorsement to the range $[0, 1]$ (see e.g., Fig. 2B).

Supplementary Results

Linear mixed-effects analysis of task behaviour

Effects of planning/goal-setting on reward-effort choice behaviour. Choice of higher-effort/ higher-reward options on each trial were analysed via linear mixed-effects models, with the within-subjects factors of time (pre *vs* post intervention) and trial number, and between-subjects factor of intervention group (planning *vs* control). In both initial and replication samples, there was a significant group*time interaction ($F_{1,8697} = 14.5; F_{1,8871} = 34.8$; both $p < 0.001$; Figure S4a,b). Follow-up pairwise comparisons with Tukey correction for multiple comparisons revealed that in the initial sample, this was due to a decrease in high-effort/high-reward choice options from time 1 to time 2 in the control ($t_{8967} = -3.96, p < 0.001$), but not the planning intervention group ($t_{8967} = 1.20, p > 0.5$). In the replication sample, this was due to a decrease in higher effort choices from time 1 to time 2 in the control group ($t_{8871} = -3.77, p < 0.001$), but increase in higher effort choices in the planning group ($t_{8871} = 4.57, p < 0.001$).

Effects of planning/goal-setting on self-reported sense of achievement, pleasure, and boredom during the reward-effort decision-making task. Participants were asked to rate their sense of achievement on successful effort exertion, pleasure on gaining rewards, and boredom levels, following each four blocks of the task. Ratings data were analysed via linear mixed-effects models, with the within-subjects factors of time (pre *vs* post intervention) and block number, and between-subjects factor of intervention group (planning *vs* control). In both initial and replication samples, there were significant group*time interaction effects on sense of achievement ($F_{1,679} = 59.3, F_{1,700} = 34.0$), pleasure on gaining rewards ($F_{1,679} = 58.8, F_{1,700} = 62.3$), and boredom ($F_{1,679} = 102.0, F_{1,700} = 14.9$; all $p < 0.001$; Figure S4c,d). Follow-up pairwise comparisons with Tukey correction for multiple comparisons revealed that in the initial sample, this was due to higher sense of achievement, higher pleasure on reward receipt, and lower boredom, at time 2 in the planning vs control group ($t_{108} = 3.35, t_{108} = 3.82, t_{103} = -3.75$; all $p < 0.01$). In the replication sample, this was due to higher sense of achievement and pleasure on reward receipt, at time 2 in the planning group ($t_{111} = 2.98, t_{110} = 2.91; p < 0.03$), and smaller increase in self-reported boredom at time 2 in the planning vs control group ($t_{700} = 10.5, t_{700} = 4.84$; both $p < 0.001$).

Goal-setting behaviour in planning intervention participants. Post-intervention, prior to each block of the task, participants in the planning intervention were asked to set an achievable goal for the amount of reward they would like to earn within that block. Participants in the control condition were asked to enter a liking rating for different kinds of online games. Participants in the planning condition tended to exceed their goals for each block (achieved > goal reward; Figure S5a,b). Analysis of ratings data via repeated-measures ANOVA with the within-subjects factor of block and between-subjects factor of intervention group revealed that in both initial and replication samples there was a significant interaction between intervention group and block number on answers ($F_{2.4,236} = 19, F_{2.5,245} = 8.9; p < 0.001$). Specifically, participants in the planning group increased the ambitiousness of their goals across blocks (difference between first and last blocks: $t_{59} = 3.70, t_{50} = 3.14; p < 0.01$), whereas participants in the control group did not show a systematic effect of block on liking ratings (difference between first and last block: $t_{41} = -2.78, t_{52} = -1.84, p < 0.07$; Figure S5a,b).

Effects of psychoeducation/reappraisal practice on attribution choice. Choices of internal (*vs* external) and global (*vs* specific) explanations for each scenario with analysed via linear

mixed-effects models with the within-subjects factors of scenario valence (positive or negative events) and time (pre *vs* post intervention), and the within-subjects factor of intervention group (psychoeducation *vs* control). In both initial discovery and replication samples, there were found to be significant interactions between time, item valence, and intervention group on frequency of internal attributions ($F_{1,6294} = 10.9, F_{1,6294} = 5.0$; both $p < 0.03$, Figure S6). Follow-up pairwise comparisons with Tukey correction for multiple comparisons revealed that this was due to a decrease in internal attribution of negative events between time 1 and time 2 in the psychoeducation group ($t_{6294} = -7.3, t_{6294} = 6.0, p < 0.001$), but not control group ($t_{6294} = -2.7, t_{6294} = 3.0, p > 0.05$). Conversely, both groups showed increased frequency of internal attributions for positive events at time 2 (psychoeducation group: $t_{6294} = 7.5, t_{6294} = 6.6$; control group: $t_{6294} = 5.6, t_{6294} = 6.5$; all $p < 0.001$). There was evidence of an interaction between time, item valence, and intervention group on frequency of choice of global attributions in the initial discovery ($F_{1,6294} = 6.1, p = 0.014$), but not the replication sample ($F_{1,6294} = 0.6, p > 0.4$).

Hierarchical Bayesian model-based analysis of task behaviour

Descriptions of posterior group-level parameter estimates and relevant sampling diagnostics for analyses presented in Figures 3-6 are listed in Tables S1-4. For each parameter, tables describe the posterior mean, standard error of the posterior mean, and 90% posterior probability quantiles. In accordance with recommendations for reporting of Bayesian analyses, tables also include the effective sample size for each parameter (an estimate of the number of independent draws from the posterior distribution of that parameter) and $\hat{R}$ (Gelman-Rubin) statistics, which index convergence across different sampling chains.

IRT-modelling of self-report data

Posterior discriminability parameters (the IRT parameter describing how well each item differentiates between individuals high and low on latent trait scores) for each sample are depicted in Fig. 6A,B. For the 'amotivation' trait, the top-discriminating items across all samples were PH9 1,4 ("*little interest or pleasure in doing things*"; "*feeling tired or having little energy*"), and AMI 5,10 ("*I make decisions firmly and without hesitation*"; "*I don't like to laze around*"; both reverse-scored). For the 'negative cognition' trait, there was more variance in the top-discriminating items across samples, as many items had similar posterior discriminability estimates. Top items across samples were mainly from the DAS scale, and included DAS 1,4,5 ("*If I don't set the highest standards for myself, I am likely to end up a second-rate person*", "*I am nothing if a person I love doesn't love me*", "*If other people know what you are really like, they will think less of you*").

Where both trait estimates were available for the same participants (i.e., the crossover study), mean posterior estimates for amotivation and negative cognition traits were moderately correlated (for participants who completed the reward-effort decision-making task, R=0.29, and for participants who completed the causal-attribution task, R=0.58, Figure S7a,d). Both trait estimates were similarly related to overall severity of depressed mood symptoms, as indexed by PHQ9 total scores (mean R =0.62, Figure S7b,c) and PHQ9 functional disability question scores (mean R=0.55, Figure S7e,f).

DEVELOPMENT AND VALIDATION OF THE CAUSAL ATTRIBUTION TASK

Due to our use of a novel scenario battery and response option structure, extensive pilot work was carried out during the development of the causal attribution task. Specifically, data from an initial pilot sample (N=102) was initially collected on the full set of items (128 total). In order to assess if we were able to consistently measure tendency to attribute events to negative and positive events to internal and global causes, data were first analysed using a simple subscores approach from classical test theory. This involved summing counts of internal and global attributions across items (64 positive, 64 negative), and then adjusting sums using the approach described in (*96*), which accounts for the existence of measurement error in observations, using the R package `subscore`. The split-half reliability for each subscore was then calculated across N=1000 random splits of the data, to generate average split-half correlation scores, using the R package `multicon`. Internal reliability statistics (Cronbach's α) were also calculated for each score, for comparison with traditional questionnaire-based measures of attributional style (Figure S8a).

Within-individuals, we observed that internal attributions for positive and negative events tended to be moderately negatively correlated (R=-0.26, Figure S8b), supporting the interpretation that individuals may vary in their tendency to express self-protective bias (i.e., heightened internal attribution of positive events, coupled with lower internal attribution of negative events). In the global-specific domain, there was a weak positive correlation between tendency to globally attribute positive and negative events (R=0.16, suggesting a more general preference for global vs specific explanations.

External validation via association with clinical scores (the 2-item version of the PHQ9, the PHQ2 (*97*), DAS, and miniSPIN total scores). We observed moderate correlations between tendency to attribute negative events to internal causes and negative self-belief (DAS) and depressed mood (PHQ2) scores (Rs=0.26-0.35). Relationships with internal attributions of positive events and in the global domain tended to be small and weak - although there was some evidence that participants higher in social anxiety (miniSPIN) scores tended to attribute positive events to internal causes less often, and negative events to global causes more often (Figure S8c,d).

The two equivalent 32-item versions of the task were then developed from using 2PL IRT modelling of the full test set. Specifically,

$$P(X = 1 \mid \theta_p, \alpha_i, \beta_i) = \exp^{\alpha_i(\theta_p \beta_i)} / (1 + \exp^{\alpha_i(\theta_p \beta_i)}) \qquad \text{(S1)}$$

where $P(X = 1)$ represents the probability of choosing a global or internal attribution for each item *i* (modelled separately for positive and negative events), α is the discriminability parameter (governing how well each item differentiates between individuals high or low on the latent trait of interest), β is the difficulty parameter (governing how high an individual must be on the latent trait in order to positively endorse the item), and θ is the participant (*p*)-level latent trait estimate (here, 'globality' and 'internality' for positive and negative events, respectively). Intuitively, the above equation describes a logistic function relating trait estimates to probability of endorsement of each item, with α values governing the slope of the function, and β values its left-right translation.

After fitting the 2PL model to the full item set, items were ranked in terms of their posterior

discriminability estimates for trait internality and globality (separately for positive and negative events), and the top-ranked items alternately assigned to form two equivalent test sets, such that they both consisted of 16 negative and 16 positive items. All posterior mean discriminability estimates for included items exceeded 1 (i.e., we had evidence that they meaningfully contributed to the construction of trait estimates).

Finally, simulation-based calibration analysis of the 32-item versions and associated inference model was then carried out, and observed test-retest reliability of task parameters from the two versions was formally assessed (see main text).

Supplementary Figures

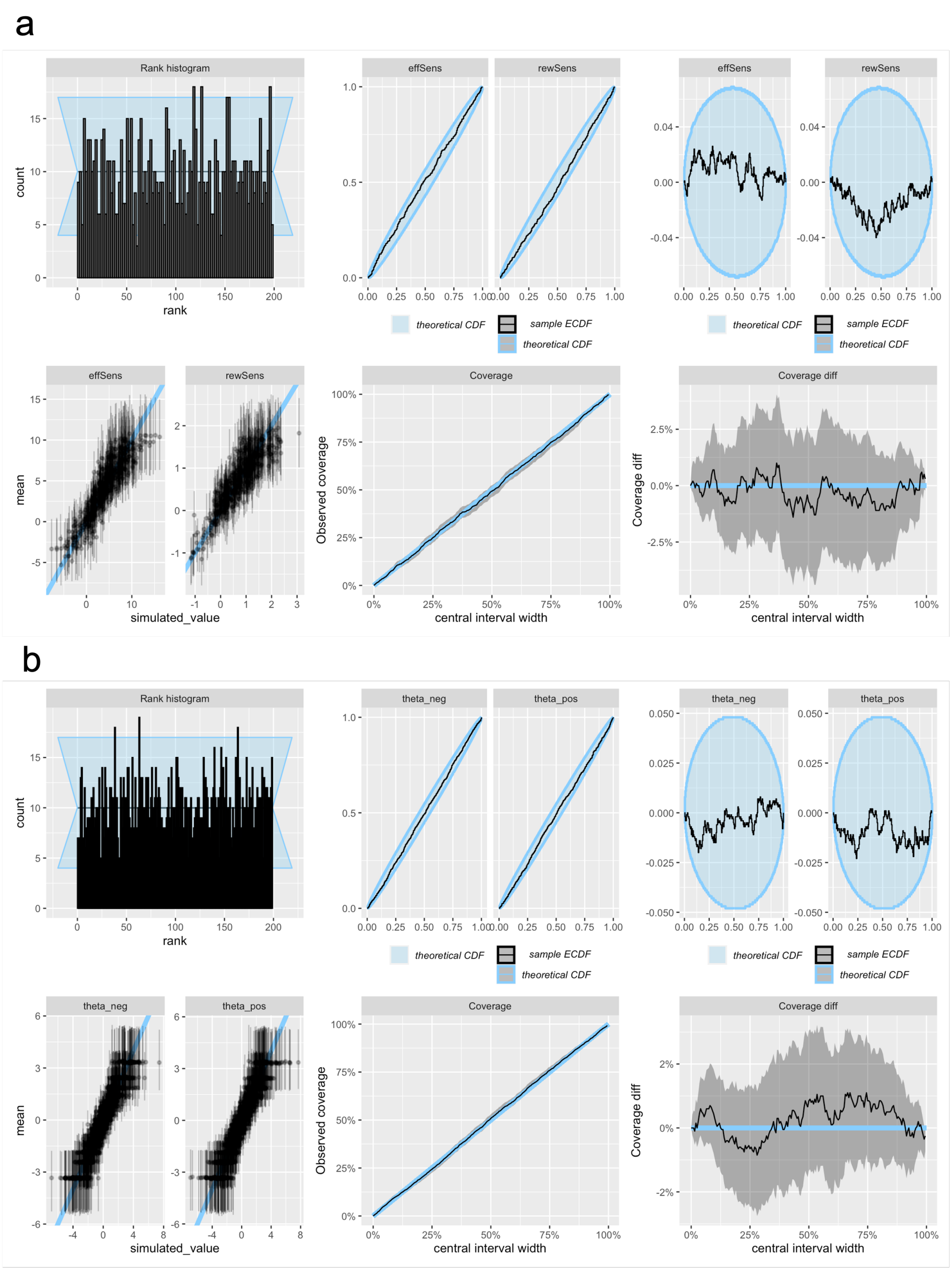

Figure S1: **Graphical summary of simulation-based calibration analysis of data generation and model fit procedures for both tasks.** **a** Reward-effort decision-making task. *effSens*, effort sensitivity parameter; *rewSens*, reward sensitivity parameter. **b** Causal-attribution task. *theta_neg*, parameter governing latent tendency to make internal/global attributions of negative events; *theta_pos*, parameter governing latent tendency to make internal/global attributions of positive events. Plots in each panel are rank histograms (check for uniformity of posterior draw ranks, see Methods; horizontal black line=expected average count, blue trapezoid=approximate 95% interval for expected deviations), (E)CDF, (empirical) cumulative distribution functions (blue ellipse=region outlining expected 95% deviations; top-right plots show are rotated by 45° for easier visualisation of deviations), and coverage plots (which show the proportion of true variable values that fall within the 95% posterior credible intervals for each parameter).

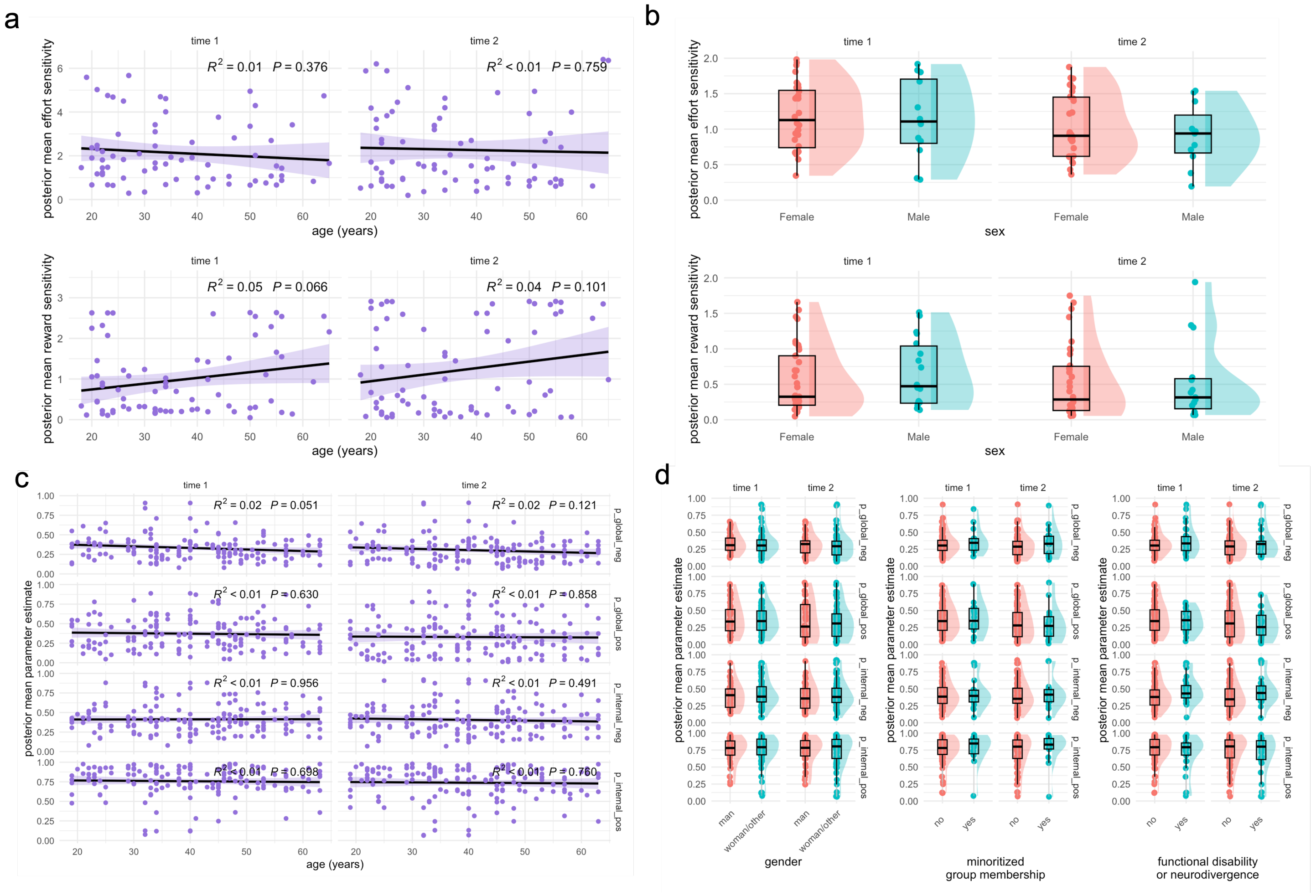

Figure S2: **Parameter estimates according to age and other sociodemographic information, in pilot data samples. a** Posterior mean effort and reward sensitivity estimates by age in years, for the reward-effort decision-making task test-retest reliability sample (N=72). **b** Posterior mean effort and reward sensitivity estimates by sex, for the reward-effort decision-making task. **c** Posterior parameter estimates by age, for the causal attribution task test-retest reliability sample (N=88). *p_global_neg/pos*, probability of attributing a negative/positive event to a global cause; *p_internal_neg/pos*, probability of attributing a negative/positive event to an internal cause. **d** Posterior parameter estimates for the causal attribution task by gender identity (man, or woman/non-binary/other), minoritized group status (identifying as belonging to a group that may lead to greater risk of being discriminated against or experiencing prejudice in social or professional situations), and functional disability or neurodivergence (disability or form of neurodivergence that affects ability to concentrate for extended periods of time, perform physically effortful activities, read/write/do maths, deal with people you don't know, or other form or impact on psychosocial functioning).

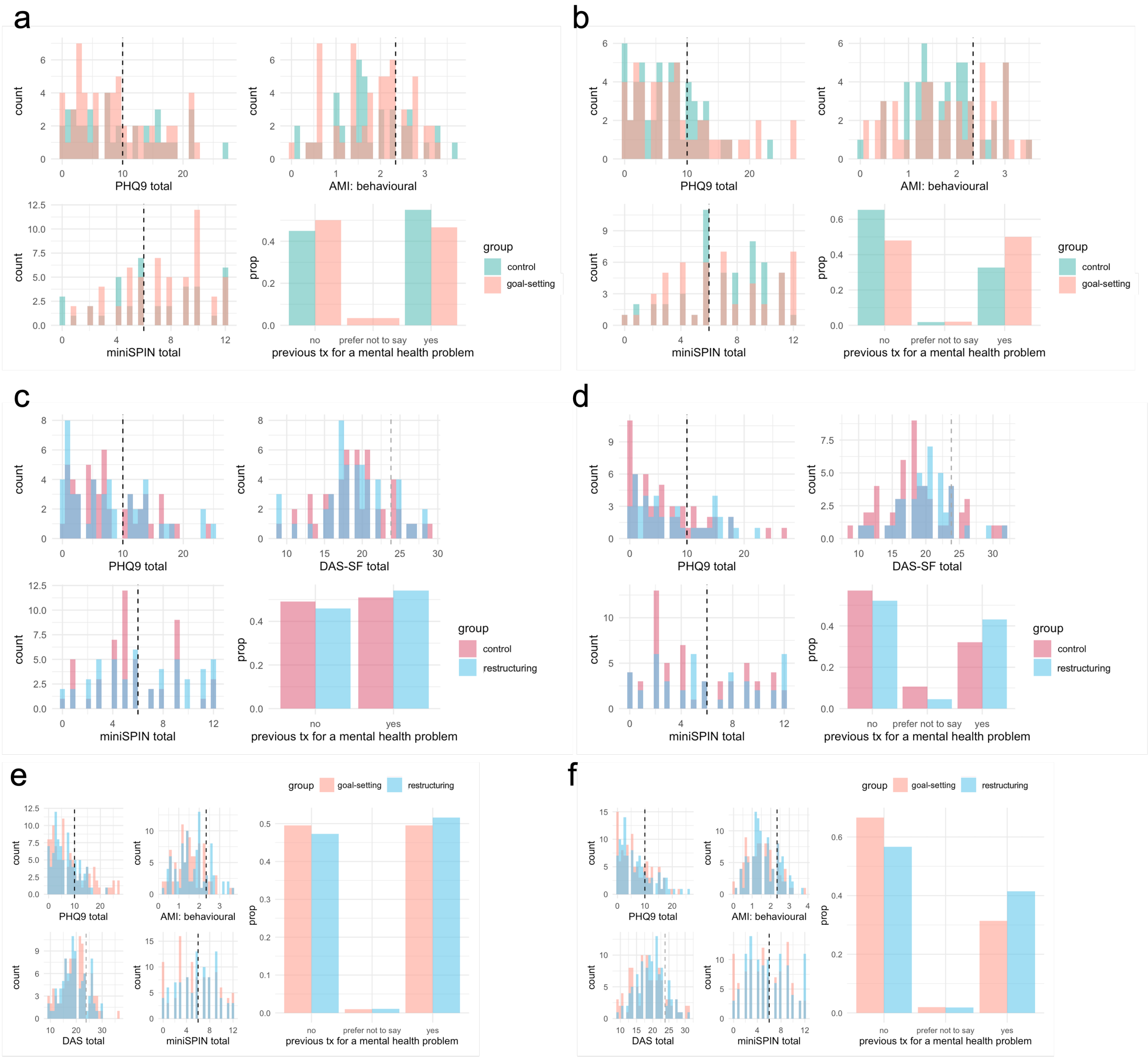

Figure S3: **Self-reported psychological symptom scores for study participants.** **a** Reward-effort decision-making initial discovery sample. **b** Reward-effort decision-making replication sample. **c** Causal attribution task initial discovery sample. **d** Causal attribution task replication sample. **e** Crossover study participants who completed the reward-effort decision-making task. **f** Crossover study participants who completed the causal attribution task. *PHQ9 total*, Physician's Health Questionnaire 9-item measure of depressed mood total score. *AMI: behavioural*, Apathy Motivational Index behavioural amotivation subscale score. *miniSPIN total*, mini Social Phobia Inventory total score. *DAS-SF total*, Dysfunctional Attitude Scale short-form total score. Black dotted lines represent previously-published cut-off scores for clinically-significant levels of symptoms. For the DAS-SF, where no such cut-off score is available, grey dotted lines represent mean scores in previously-published samples of depressed in-patients. Participants were also asked if they had ever previously received treatment (tx) for a mental health problem (see Table 1).

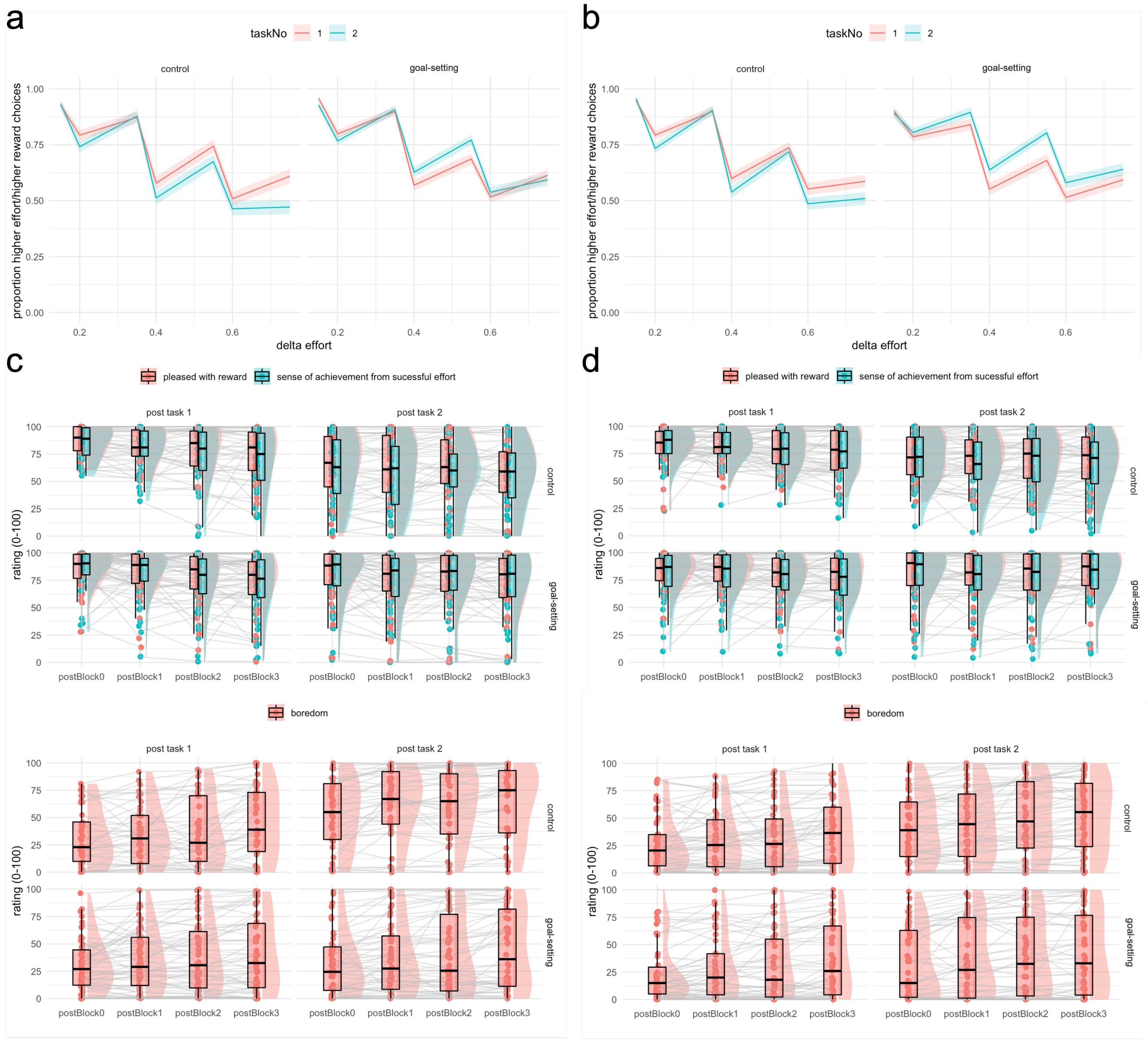

Figure S4: **Choice data and self-report ratings from the reward-effort decision-making task.** **a** Proportionate choice of higher effort/higher reward options by difference in required effort level between choices (delta effort), pre- and post-intervention (taskNo 1, taskNo 2), by intervention condition, in the initial discovery sample. **b** The same data as in (a), in the replication sample. **c** Self-reported ratings data collected after each block of the task, by time and intervention condition. Pleased with reward, "During the task, did you feel PLEASED when you collected the coins?"; sense of achievement from successful effort, "During the task, did you feel A SENSE OF ACHIEVEMENT when you collected the coins?"; boredom, "During the task, did you feel BORED?". **d** The same data as in (c), in the replication sample.

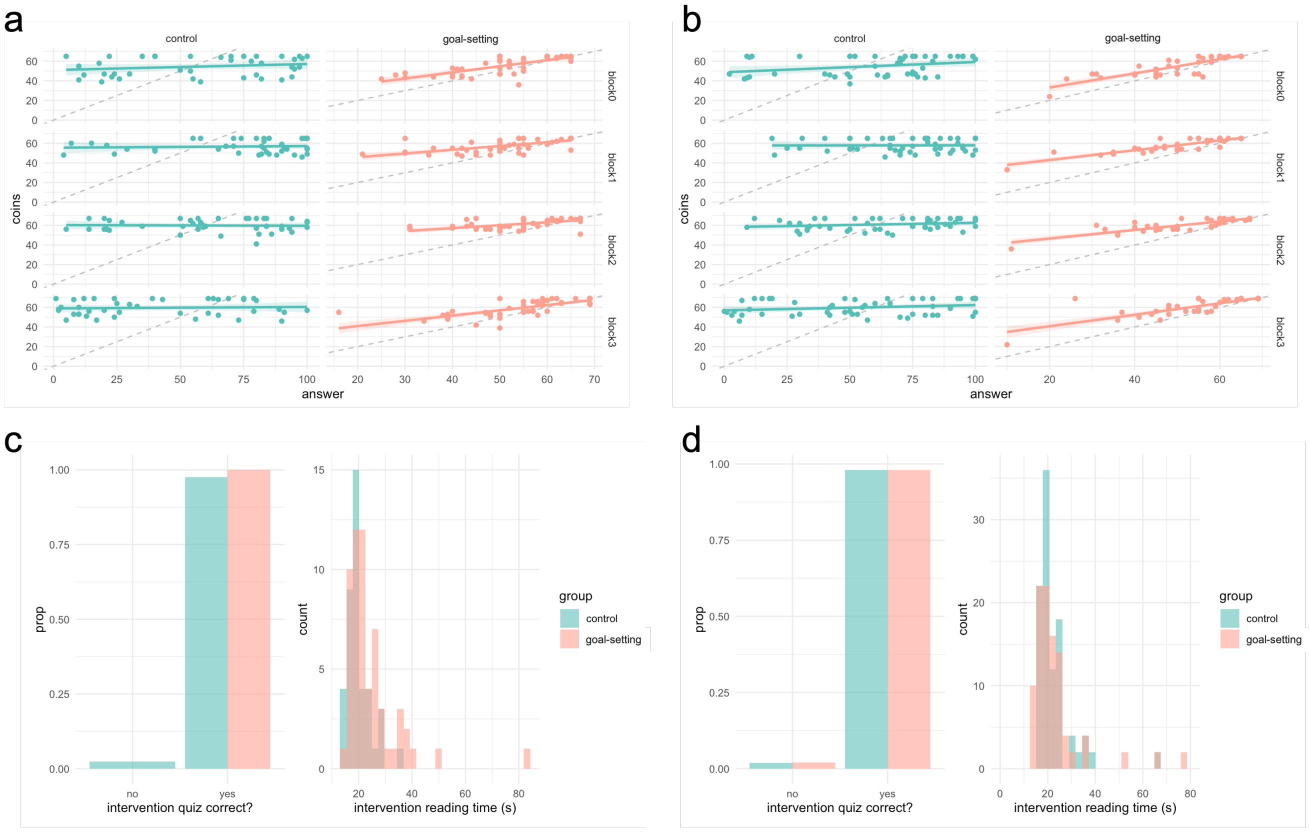

Figure S5: **Goal-setting and intervention reading time data for the reward-effort decision-making task. a** Goals for each block of the task (at time 2) for participants in the initial discovery sample. Participants were invited to set a goal prior to completing each block, given the information that the maximum available reward per block was 69 coins, if they chose the highest effort option every time. For the control condition, answer values represent liking ratings for different types of computer games. Coins represent the actual reward earned by participants in that block. **b** The same information as (a), for the replication sample. **c** *Left*, proportion of participants who provided the correct answer to the multiple-choice comprehension quiz, which followed the intervention text (participants were allowed to return and re-read the text prior to answering). *Right*, time spent reading the intervention text (a single screen of information), in discovery sample participants. **d** The same information as in (c), for the replication sample participants.

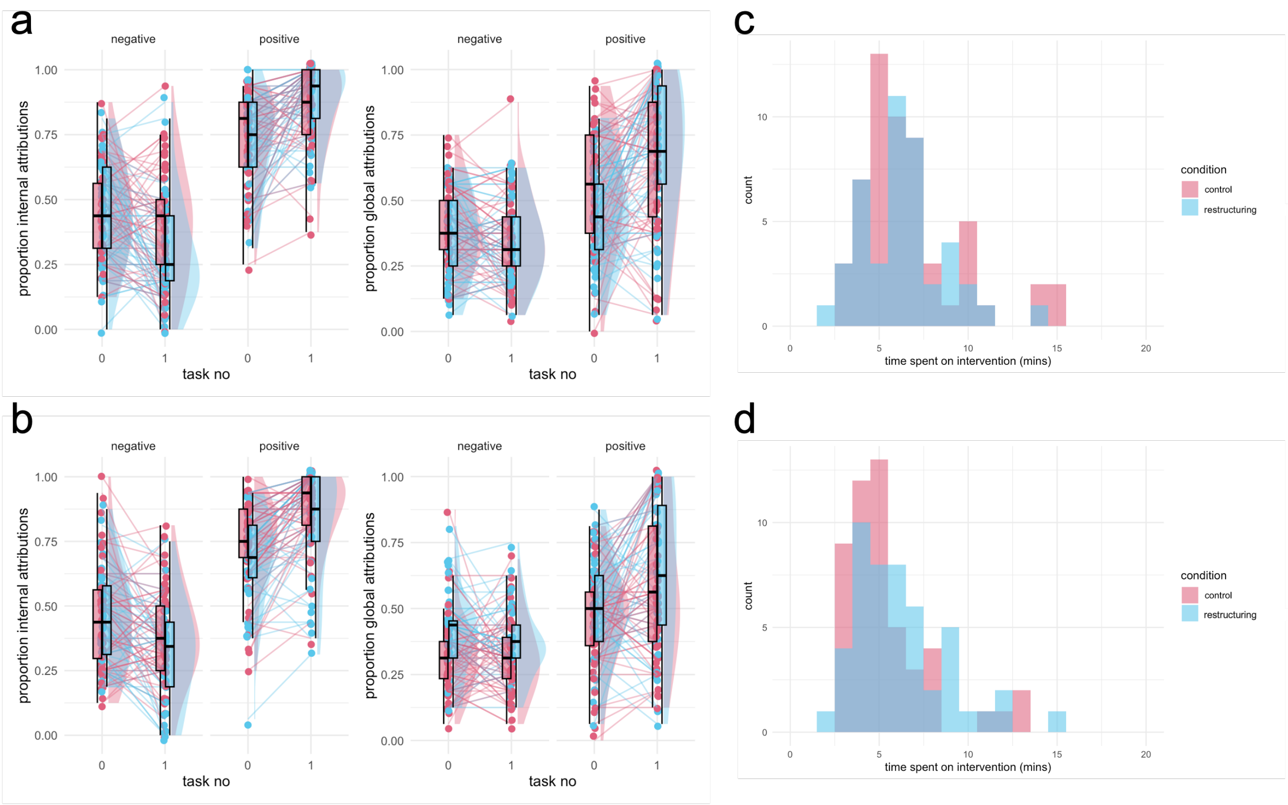

Figure S6: **Attribution choices and intervention time data for the causal attribution task.** **a** Proportionate choice of internal (vs external), and global (vs specific) attributions chosen for positive and negative events pre- and post-intervention (task no 0, task no 1), in the initial discovery sample. **b** The same data as in (a), for the replication sample. **c** Time spent on the intervention in each condition in the initial discovery sample. **d** The same data as in (c), for the replication sample participants.

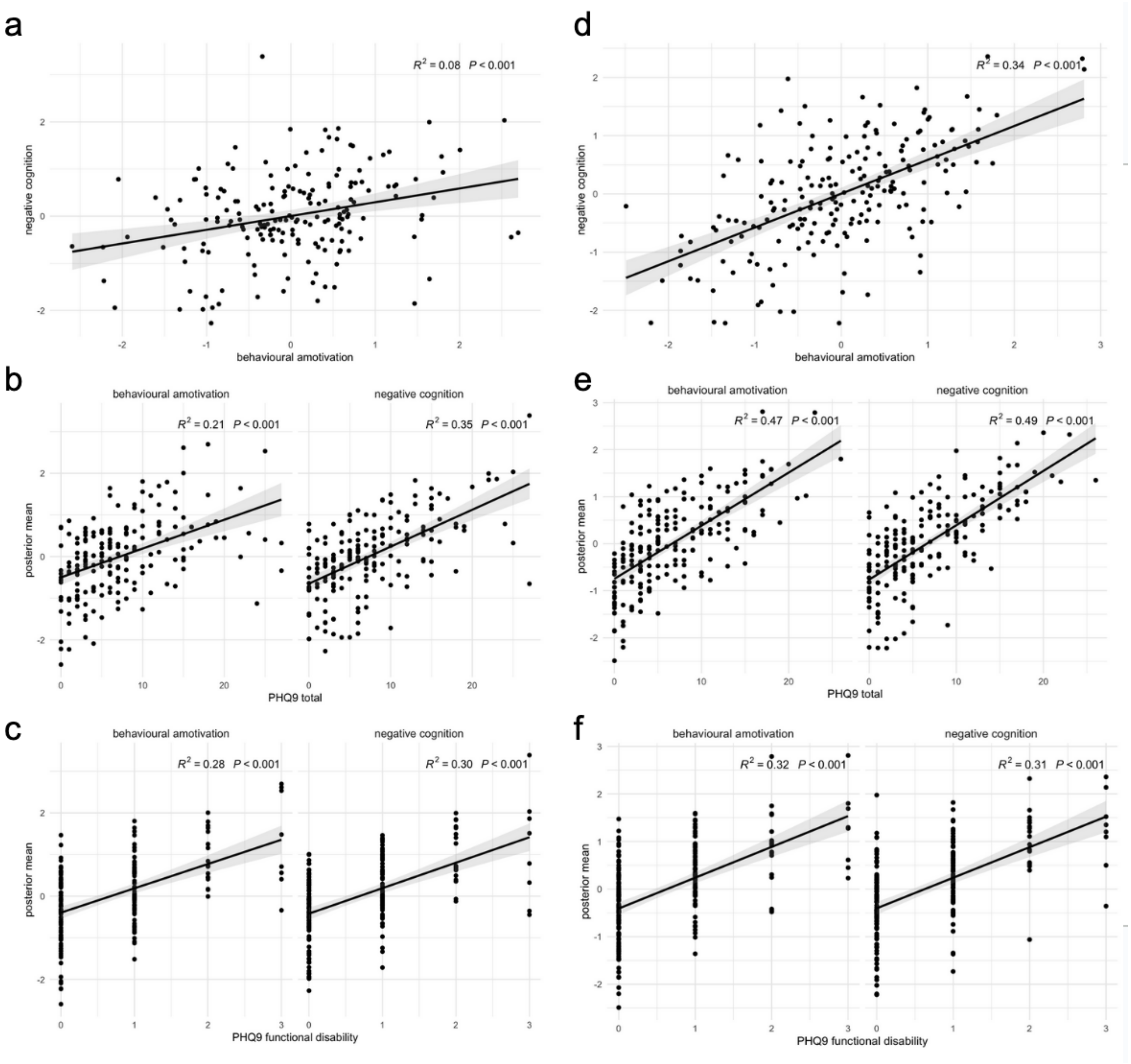

Figure S7: **Relationships between amotivation and negative cognition trait estimates in crossover study participants.** **a** Relationship between mean posterior estimates for trait behavioural amotivation and negative cognition in crossover study participants who completed the reward-effort decision-making task (N=185). **b** Relationships between mean posterior trait estimates and overall depression symptom severity, as measured by PHQ9 total scores. **c** Relationships between mean posterior trait estimates and overall depression symptom severity, as measured by PHQ9 functional disability item scores (”*How difficult have these problems made it for you to do your work, take care of things at home, or get along with other people?*”). **d,e,f** The same plots as (a,b,c), for the crossover study participants who completed the causal attribution task (N=205).

a

mean split-half item correlations and subscore reliabilities

	N Vars	Mean Split-Half r	Rel	Rel SD
internal negative	64	0.824	0.903	0.083
internal positive	64	0.868	0.929	0.091
global negative	64	0.770	0.870	0.077
global positive	64	0.816	0.899	0.088

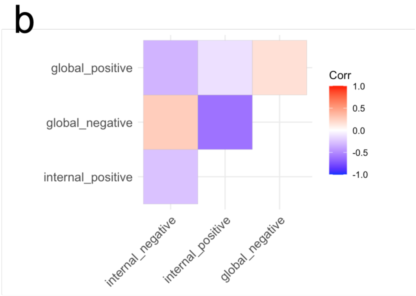

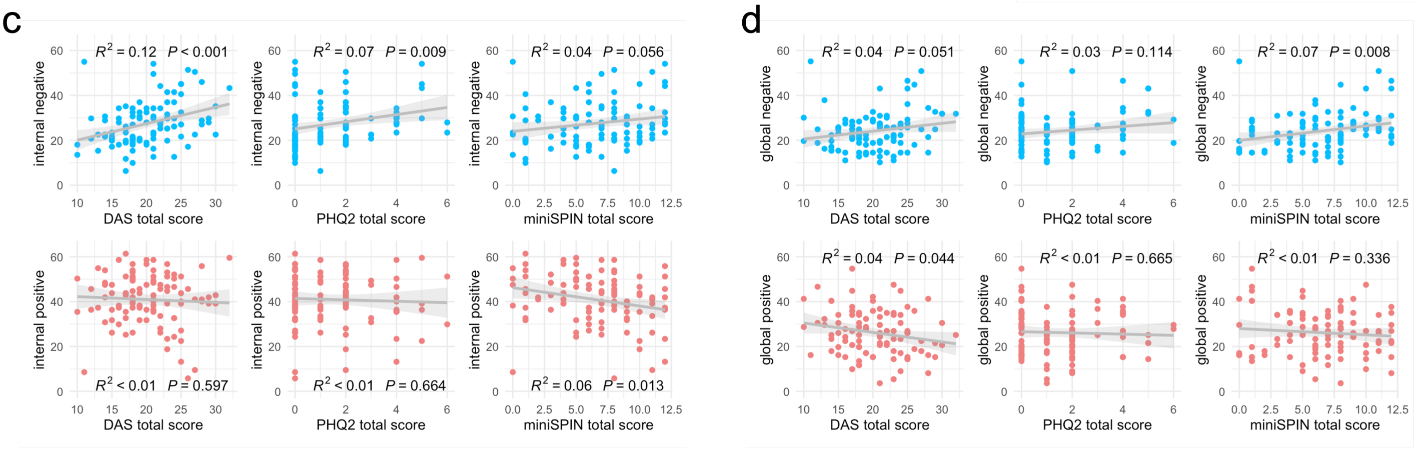

Figure S8: **Details of split-half reliability, within-participant correlation structure, and relationship to clinical scores of simple subscores derived from the full causal attribution task battery, during task development.** **a** Split-half reliability and internal consistency estimates for the full (128) item set in N=102 pilot study participants. *Mean Split-Half r*, the average of all estimated split-half correlations; *Rel*, the average of all split-half reliabilities (equivalent to Cronbach's alpha); *Rel SD*, the standard deviation of all split-half reliabilities. **b** Correlation matrix for within-participant variation in subscore estimates. **c** Bivariate relationships between negative and positive internality subscores, and self-reported clinical symptoms. d Bivariate relationships between negative and positive globality subscores, and self-reported clinical symptoms. *DAS*, Dysfunctional Attitudes Scale (short form). *PHQ2*, Patient Health Questionnaire 2-item measure of depressed mood, *miniSPIN*, 3-item mini Social Phobia Inventory.

Supplementary Tables

	mean	se (mean)	sd	10%	90%	N_{eff}	$\hat{R}$
**Initial discovery sample**							
Mean effort sensitivity at time 1	-0.673	0.004	0.140	-0.976	-0.450	1061	1.004
Mean effort sensitivity at time 2	-0.554	0.004	0.165	-0.768	-0.235	1385	1.003
Mean reward sensitivity at time 1	0.308	0.003	0.088	0.199	0.423	677	1.004
Mean reward sensitivity at time 2	0.301	0.005	0.133	0.131	0.473	838	1.003
Effect of goal-setting on reward sensitivity at time 2	0.114	0.003	0.123	-0.041	0.270	1818	1.001
Effect of goal-setting on effort sensitivity at time 2	-0.318	0.004	0.182	-0.547	-0.092	2179	1.000
**Replication sample**							
Mean effort sensitivity at time 1	-0.772	0.005	0.160	-0.976	-0.577	854	1.005
Mean effort sensitivity at time 2	-0.722	0.005	0.168	-0.936	-0.515	1155	1.001
Mean reward sensitivity at time 1	0.293	0.003	0.088	0.185	0.405	695	1.003
Mean reward sensitivity at time 2	0.382	0.005	0.138	0.211	0.564	862	1.003
Effect of goal-setting on reward sensitivity at time 2	0.183	0.003	0.123	0.029	0.341	1680	1.000
Effect of goal-setting on effort sensitivity at time 2	-0.370	0.004	0.187	-0.613	-0.135	1886	1.000

Table S1: **Hierarchical Bayesian model results for effect of goal-setting on reward-effort decision-making.** Mean, posterior mean; se (mean), standard error of the posterior mean. 10%, 90%, posterior probability quantiles for parameter estimates; N_{eff}, effective sample size (an estimate of the number of independent draws from the posterior distribution of the estimand of interest); $\hat{R}$, the ratio of the average variance of draws within each chain to the variance of the pooled draws across chains (if all chains are at equilibrium, $\hat{R}$ will be 1). All values are raw (untransformed) parameter estimates (for transformation constraints applied to main text figures see Supplementary Methods).

	mean	se (mean)	sd	10%	90%	N_{eff}	$\hat{R}$
**Initial discovery sample**							
Mean θ for internal attributions of negative events at time 1	-0.224	0.002	0.081	-0.329	-0.121	2351	1.001
Mean θ for internal attributions of negative events at time 2	-0.483	0.003	0.136	-0.660	-0.309	2288	1.001
Mean θ for internal attributions of positive events at time 1	1.100	0.002	0.098	0.980	1.225	2893	1.001
Mean θ for internal attributions of positive events at time 2	2.241	0.005	0.225	1.963	2.539	1762	1.003
Mean θ for global attributions of negative events at time 1	-0.510	0.001	0.069	-0.600	-0.421	3498	0.999
Mean θ for global attributions of negative events at time 2	-0.668	0.002	0.100	-0.799	-0.544	3170	0.999
Mean θ for global attributions of positive events at time 1	-0.014	0.002	0.095	-0.141	0.107	2288	1.000
Mean θ for global attributions of positive events at time 2	0.704	0.005	0.218	0.427	0.987	1650	1.003
Effect of restructuring on θ internal-negative at time 2	-0.563	0.004	0.190	-0.806	-0.315	2956	1.001
Effect of restructuring on θ internal-positive at time 2	0.385	0.007	0.304	-0.010	0.770	1836	1.001
Effect of restructuring on θ global-negative at time 2	0.009	0.002	0.136	-0.164	0.184	3555	1.000
Effect of restructuring on θ global-positive at time 2	0.472	0.008	0.303	0.078	0.858	1572	1.002
**Replication sample**							
Mean θ for internal attributions of negative events at time 1	-0.199	0.002	0.085	-0.309	-0.091	2015	1.002
Mean θ for internal attributions of negative events at time 2	-0.509	0.002	0.114	-0.654	-0.364	2405	1.001
Mean θ for internal attributions of positive events at time 1	0.975	0.002	0.097	0.852	1.102	2041	1.002
Mean θ for internal attributions of positive events at time 2	2.285	0.005	0.218	2.011	2.561	2230	1.000
Mean θ for global attributions of negative events at time 1	-0.610	0.001	0.071	-0.698	-0.520	2675	1.001
Mean θ for global attributions of negative events at time 2	-0.780	0.001	0.083	-0.888	-0.672	3585	1.000
Mean θ for global attributions of positive events at time 1	-0.099	0.002	0.080	-0.203	0.005	2106	1.001
Mean θ for global attributions of positive events at time 2	0.435	0.005	0.200	0.183	0.685	1888	1.002
Effect of restructuring on θ internal-negative at time 2	-0.337	0.003	0.171	-0.556	-0.123	2501	1.001
Effect of restructuring on θ internal-positive at time 2	0.149	0.006	0.301	-0.240	0.524	2180	1.000
Effect of restructuring on θ global-negative at time 2	0.208	0.002	0.123	0.051	0.363	3271	1.000
Effect of restructuring on θ global-positive at time 2	0.435	0.006	0.280	0.077	0.789	2100	1.001

Table S2: **Hierarchical Bayesian model results for effect of cognitive restructuring on causal attribution.** *Continued on next page.*

Table S2: θ, parameters describing latent tendency to attribute events to different kinds of causes. Mean, posterior mean; se (mean), standard error of the posterior mean. 10%, 90%, posterior probability quantiles for parameter estimates; N_{eff}, effective sample size; $\hat{R}$, the ratio of the average variance of draws within each chain to the variance of the pooled draws across chains). All values are raw parameter estimates.

	mean	se (mean)	sd	10%	90%	N_{eff}	$\hat{R}$
**Reward-effort decision-making task sample**							
Mean effort sensitivity at time 1	-0.804	0.003	0.090	-0.920	-0.691	1078	1.002
Mean effort sensitivity at time 2	-0.727	0.004	0.126	-0.890	-0.569	1213	1.001
Mean reward sensitivity at time 1	0.309	0.003	0.066	0.225	0.394	510	1.007
Mean reward sensitivity at time 2	0.549	0.004	0.114	0.408	0.700	849	1.002
Effect of goal-setting *vs* restructuring on reward sensitivity at time 2	-0.138	0.003	0.104	-0.271	-0.004	1291	1.002
Effect of goal-setting *vs* restructuring on effort sensitivity at time 2	-0.693	0.004	0.153	-0.892	-0.501	1855	1.001
**Causal attribution task sample**							
Mean θ for internal attributions of negative events at time 1	-0.236	0.001	0.054	-0.305	-0.167	1633	1.002
Mean θ for internal attributions of negative events at time 2	-0.011	0.002	0.075	-0.107	0.085	1970	1.001
Mean θ for internal attributions of positive events at time 1	0.940	0.001	0.059	0.865	1.016	1631	1.001
Mean θ for internal attributions of positive events at time 2	0.967	0.003	0.109	0.827	1.107	1476	1.002
Mean θ for global attributions of negative events at time 1	-0.483	0.001	0.052	-0.550	-0.416	2373	1.000
Mean θ for global attributions of negative events at time 2	-0.421	0.002	0.077	-0.520	-0.321	2135	1.000
Mean θ for global attributions of positive events at time 1	-0.116	0.001	0.054	-0.184	-0.048	2136	1.000
Mean θ for global attributions of positive events at time 2	-0.341	0.002	0.088	-0.454	-0.231	1888	0.999
Effect of restructuring *vs* goal-setting on θ internal-negative at time 2	-0.276	0.002	0.097	-0.399	-0.151	2723	1.001
Effect of restructuring *vs* goal-setting on θ internal-positive at time 2	0.460	0.003	0.143	0.272	0.645	2531	1.001
Effect of restructuring *vs* goal-setting on θ global-negative at time 2	-0.146	0.002	0.100	-0.269	-0.014	2762	1.002
Effect of restructuring *vs* goal-setting on θ global-positive at time 2	0.065	0.002	0.115	-0.080	0.208	2519	0.999

Table S3: **Hierarchical Bayesian model results for effects of goal-setting vs restructuring on reward-effort decision-making and causal attribution in the crossover study.** *Continued on next page.*

Table S3: θ, parameters describing latent tendency to attribute events to different kinds of causes. Mean, posterior mean; se (mean), standard error of the posterior mean. 10%, 90%, posterior probability quantiles for parameter estimates; N_{eff}, effective sample size; $\hat{R}$, the ratio of the average variance of draws within each chain to the variance of the pooled draws across chains. All values are raw (untransformed) parameter estimates.

	**mean**	**se (mean)**	**sd**	**10%**	**90%**	N_{eff}	$\hat{R}$
**Reward-effort decision-making initial + replication samples**							
Effect of goal-setting on reward sensitivity	0.120	0.004	0.110	-0.023	0.261	805	1.007
Effect of goal-setting on effort sensitivity	-0.321	0.003	0.120	-0.477	-0.174	1626	1.002
βa, baseline effort sensitivity	0.231	0.004	0.128	0.072	0.396	961	1.001
βa, effect of goal-setting on effort sensitivity	-0.371	0.004	0.141	-0.548	-0.194	1283	1.000
**Crossover study reward-effort decision-making sample**							
Effect of goal-setting on reward sensitivity	-0.151	0.003	0.102	-0.278	-0.025	1561	1.000
Effect of goal-setting on effort sensitivity	-0.342	0.003	0.143	-0.522	-0.162	1945	1.001
βa, baseline effort sensitivity	0.052	0.003	0.083	-0.050	0.158	931	1.005
βn, baseline effort sensitivity	-0.078	0.003	0.085	-0.188	0.029	1007	1.007
βa, effect of goal-setting on effort sensitivity	-0.015	0.004	0.128	-0.178	0.148	1243	1.006
βn, effect of goal-setting on effort sensitivity	-0.016	0.003	0.118	-0.165	0.133	1360	1.005
**Causal attribution task initial + replication samples**							
Effect of restructuring on θ internal-negative attributions	-0.477	0.003	0.139	-0.655	-0.298	2098	1.001
Effect of restructuring on θ internal-positive attributions	0.362	0.007	0.267	0.021	0.702	1431	1.000
Effect of restructuring on θ global-negative attributions	0.070	0.002	0.092	-0.046	0.187	3290	0.999
Effect of restructuring on θ global-positive attributions	0.497	0.006	0.242	0.191	0.813	1819	0.999
βn, baseline θ internal-positive	-0.161	0.002	0.078	-0.263	-0.062	1160	1.002
βn, effect of restructuring on θ internal-positive	-0.238	0.005	0.191	-0.483	0.004	1414	1.002
**Crossover study causal attribution sample**							
Effect of restructuring on θ internal-negative attributions	-0.309	0.002	0.100	-0.435	-0.182	1974	1.001
Effect of restructuring on θ internal-positive attributions	0.520	0.003	0.147	0.334	0.712	1833	1.002
Effect of restructuring on θ global-negative attributions	-0.154	0.002	0.105	-0.289	-0.020	2157	1.002
Effect of restructuring on θ global-positive attributions	0.067	0.003	0.121	-0.087	0.224	1929	1.006
βa, baseline θ internal-positive	-0.114	0.002	0.075	-0.209	-0.017	1021	1.003
βn, baseline θ internal-positive	-0.114	0.002	0.073	-0.196	-0.009	1090	1.002
βa, effect of restructuring on θ internal-positive	-0.301	0.005	0.155	-0.502	-0.102	1016	1.001
βn, effect of restructuring on θ internal-positive	0.067	0.005	0.162	-0.140	0.278	911	1.004

Table S4: **Hierarchical Bayesian model results for models taking into account individual differences in self-reported behavioural amotivation and negative cognition.** *Continued on next page.*

Table S4: βa, posterior weight for influence of behavioural amotivation on baseline or intervention-induced change in parameter estimates; βn, posterior weight for influence of negative cognition on baseline or intervention-induced change in parameter estimates; θ, parameters describing latent tendency to attribute events to different kinds of causes. Mean, posterior mean; se (mean), standard error of the posterior mean. 10%, 90%, posterior probability quantiles for parameter estimates; N_{eff}, effective sample size; $\hat{R}$, the ratio of the average variance of draws within each chain to the variance of the pooled draws across chains. All values are raw (untransformed) parameter estimates.

Other Supplementary Files

Supplement 1. Study protocol. Available at the study Zenodo repository.

Supplement 2. Full screenshots of the reward-effort decision-making task, and the goal-setting and control interventions for reward-effort decision-making studies.

Reward-effort decision-making studies

Task 1

Instructions:

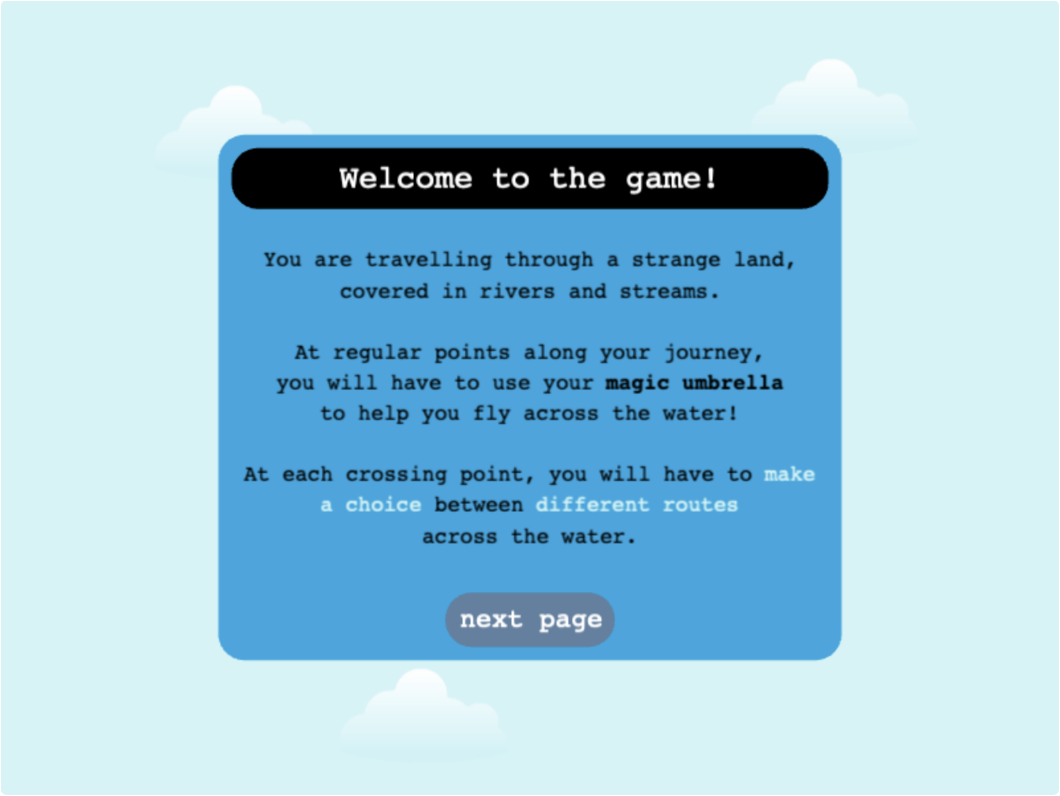

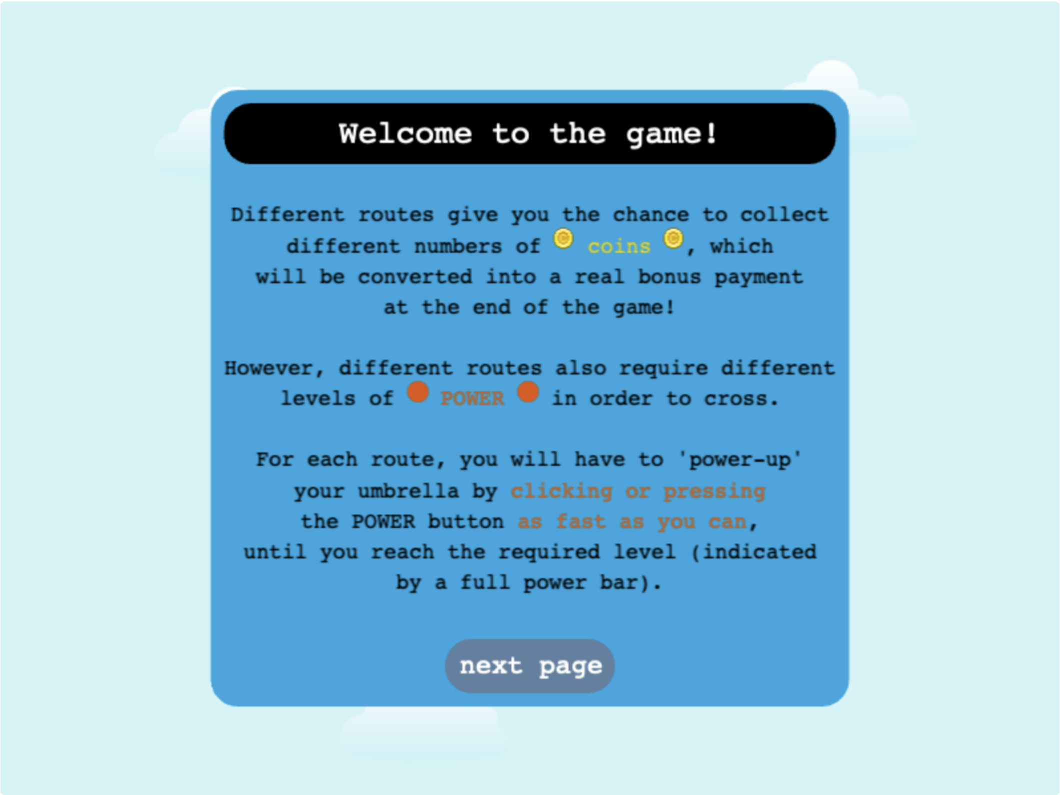

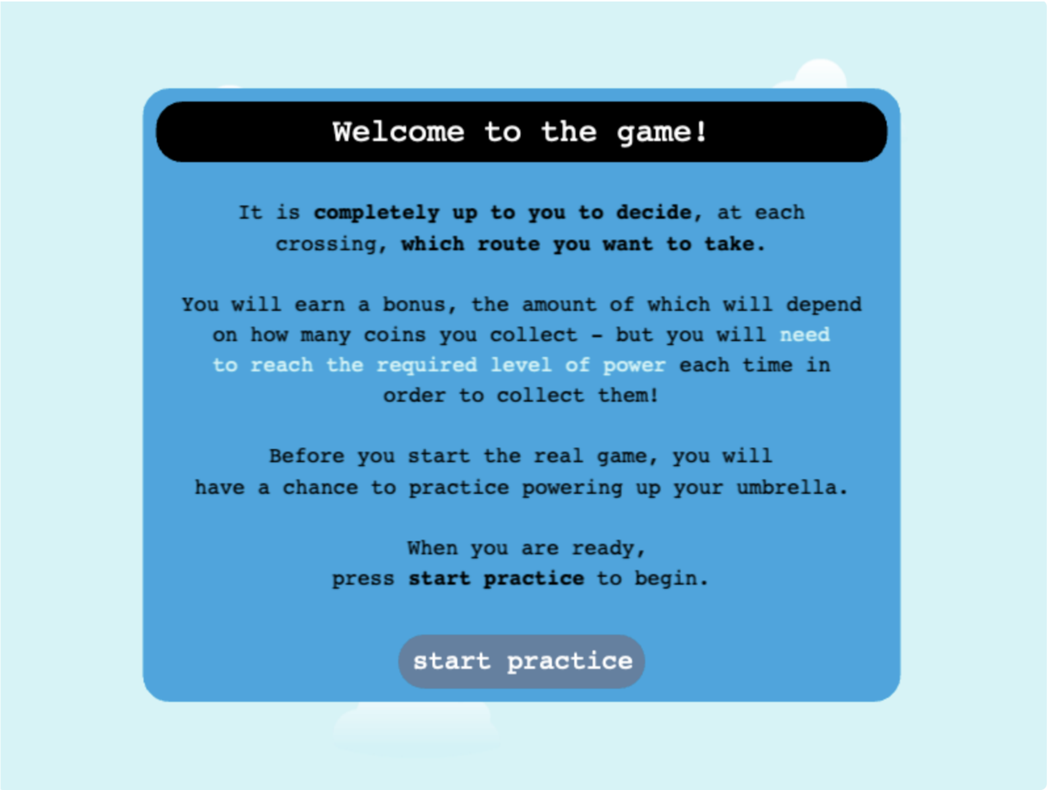

Practice (effort calibration):

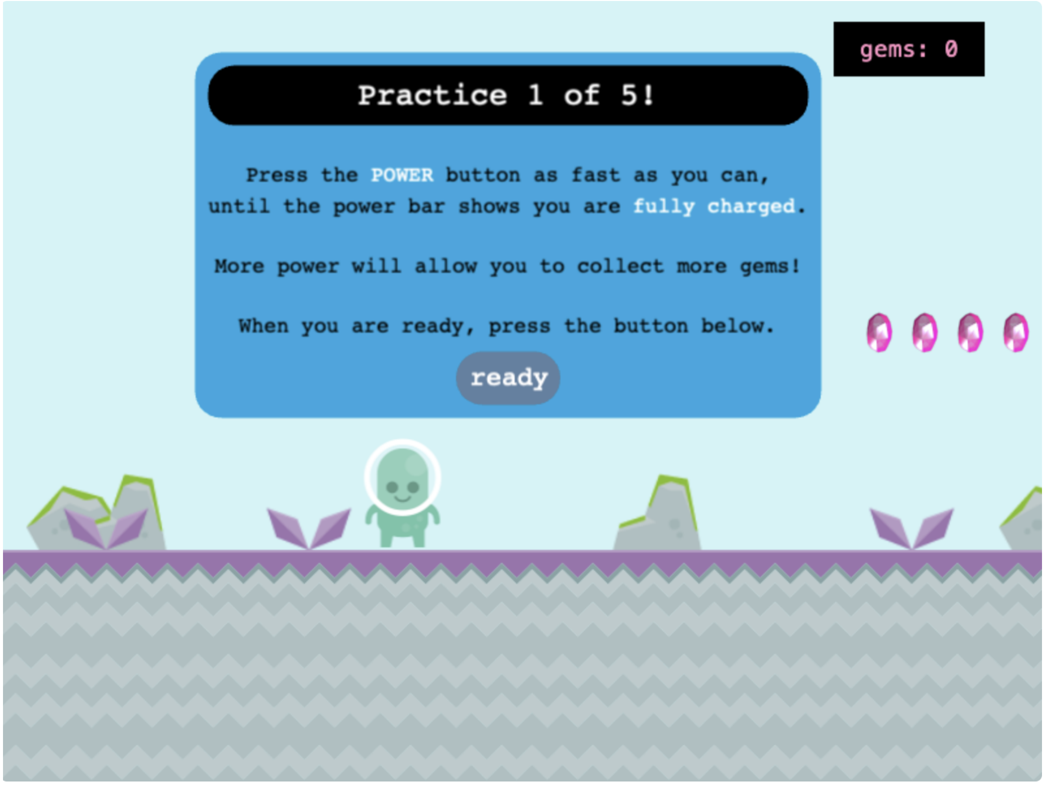

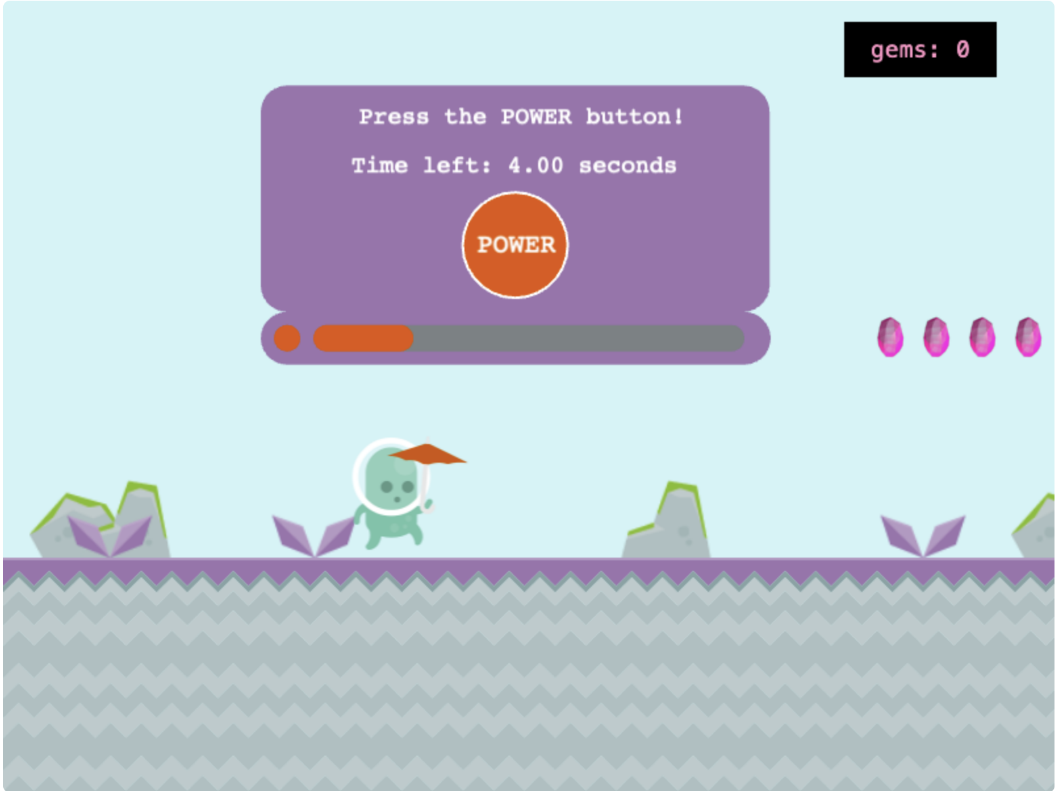
gems: 0
Press the POWER button!
Time left: 4.00 seconds
POWER

Main task:

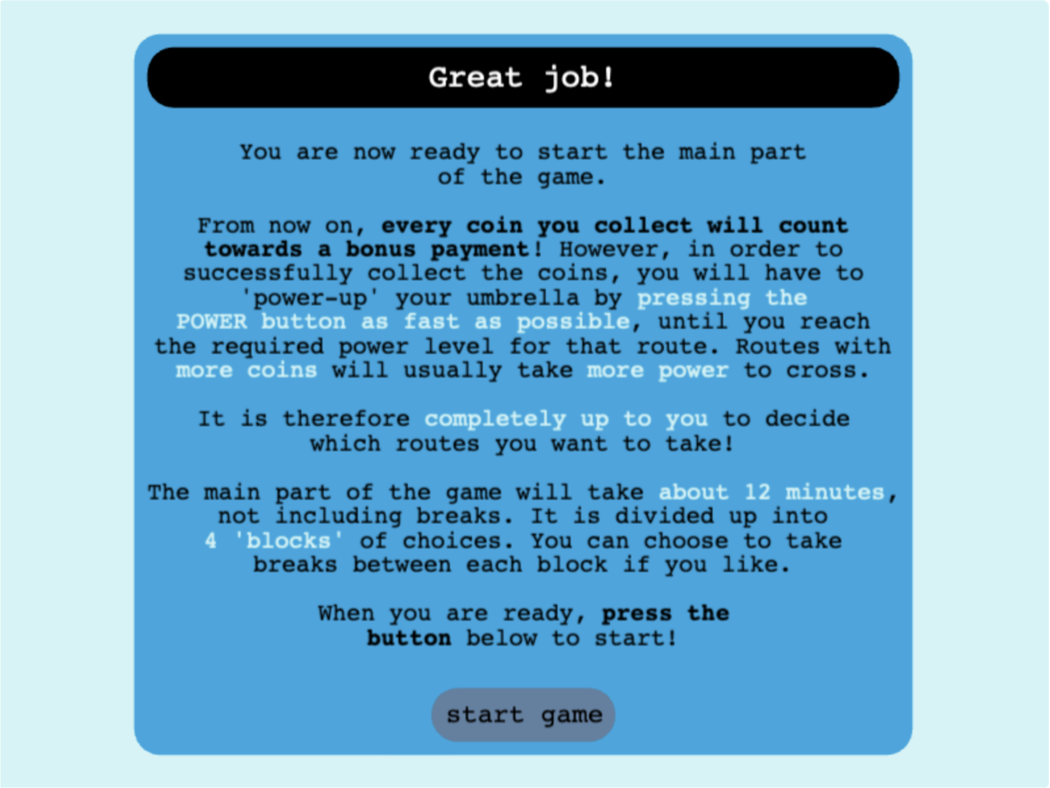
Great job!
You are now ready to start the main part of the game.
From now on, every coin you collect will count towards a bonus payment! However, in order to successfully collect the coins, you will have to 'power-up' your umbrella by pressing the POWER button as fast as possible, until you reach the required power level for that route. Routes with more coins will usually take more power to cross.
It is therefore completely up to you to decide which routes you want to take!
The main part of the game will take about 12 minutes, not including breaks. It is divided up into 4 'blocks' of choices. You can choose to take breaks between each block if you like.
When you are ready, press the button below to start!
start game

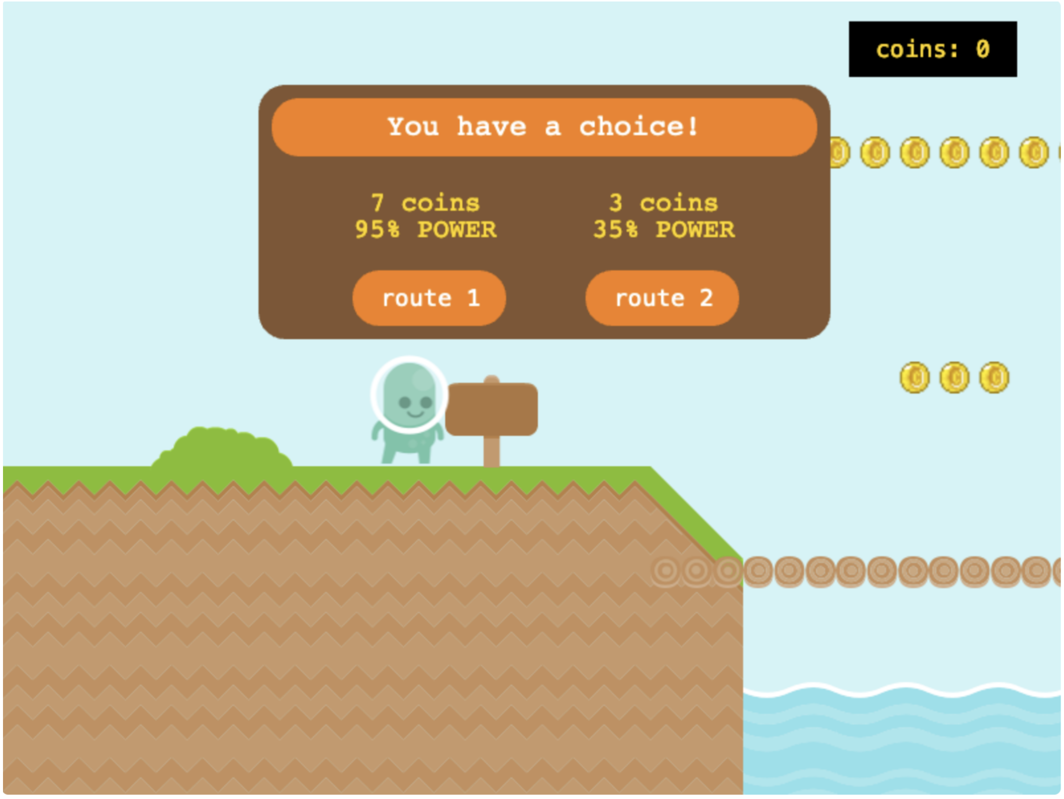
coins: 0
You have a choice!
7 coins
95% POWER
3 coins
35% POWER
route 1
route 2

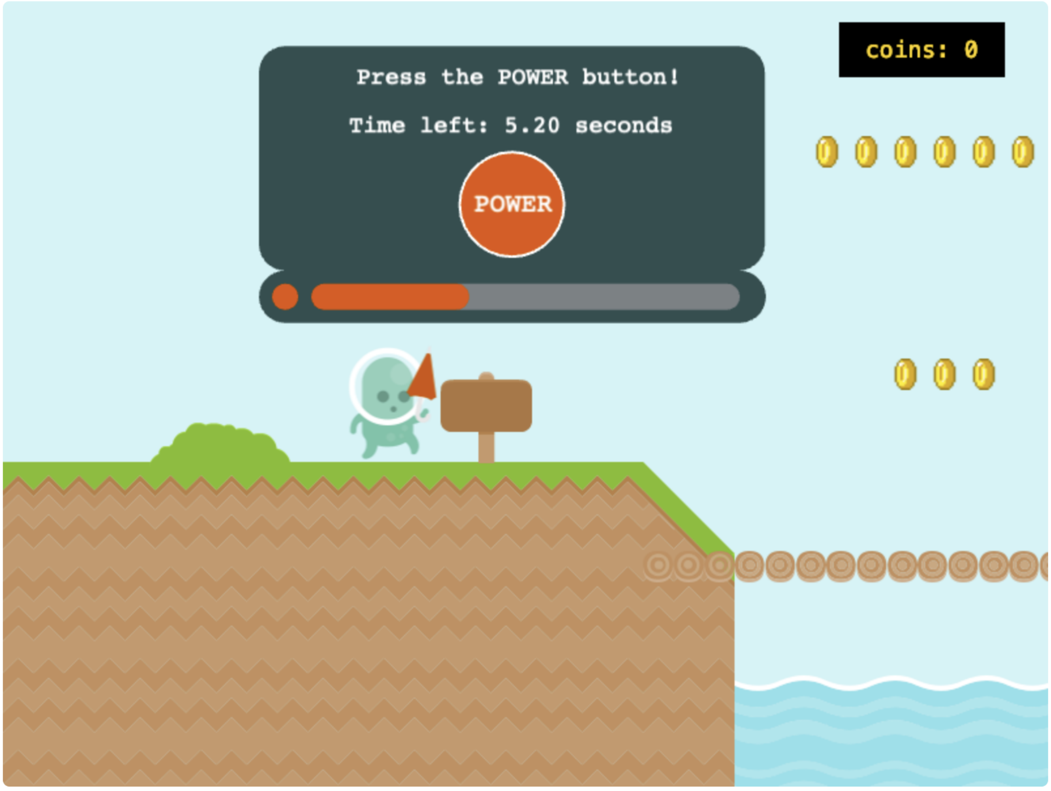
coins: 0
Press the POWER button!
Time left: 5.20 seconds
POWER

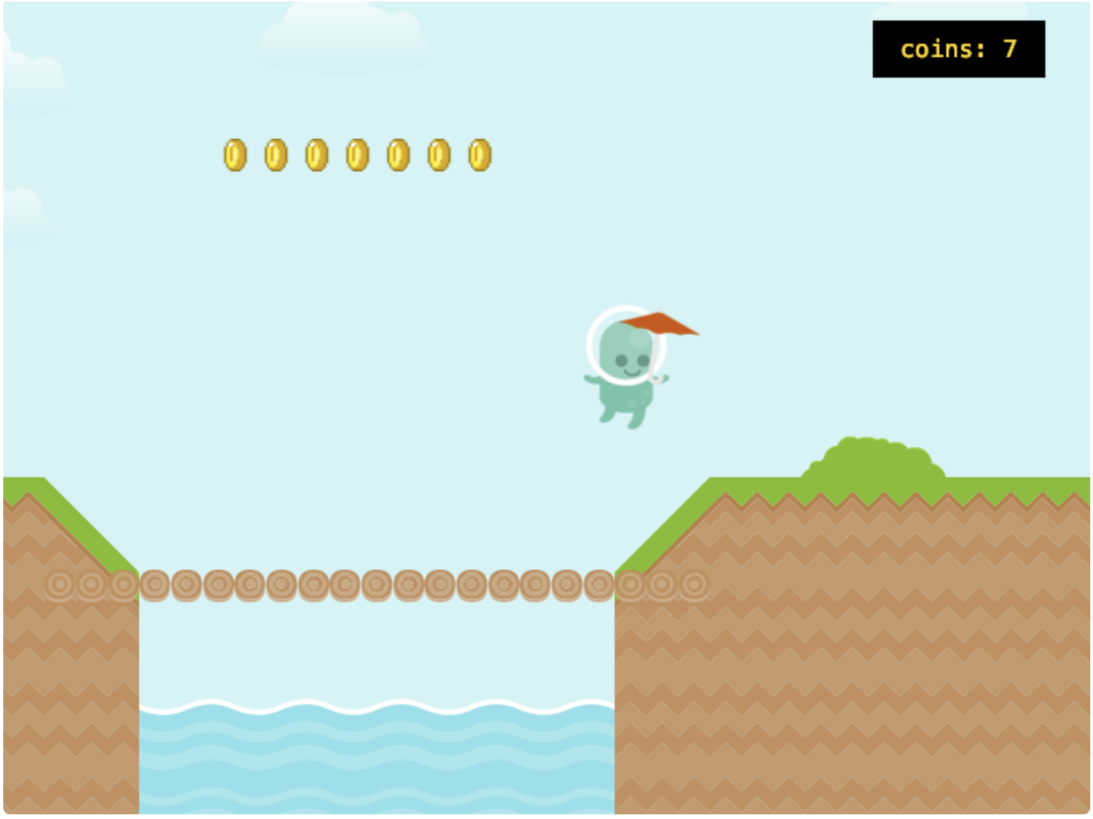
coins: 7

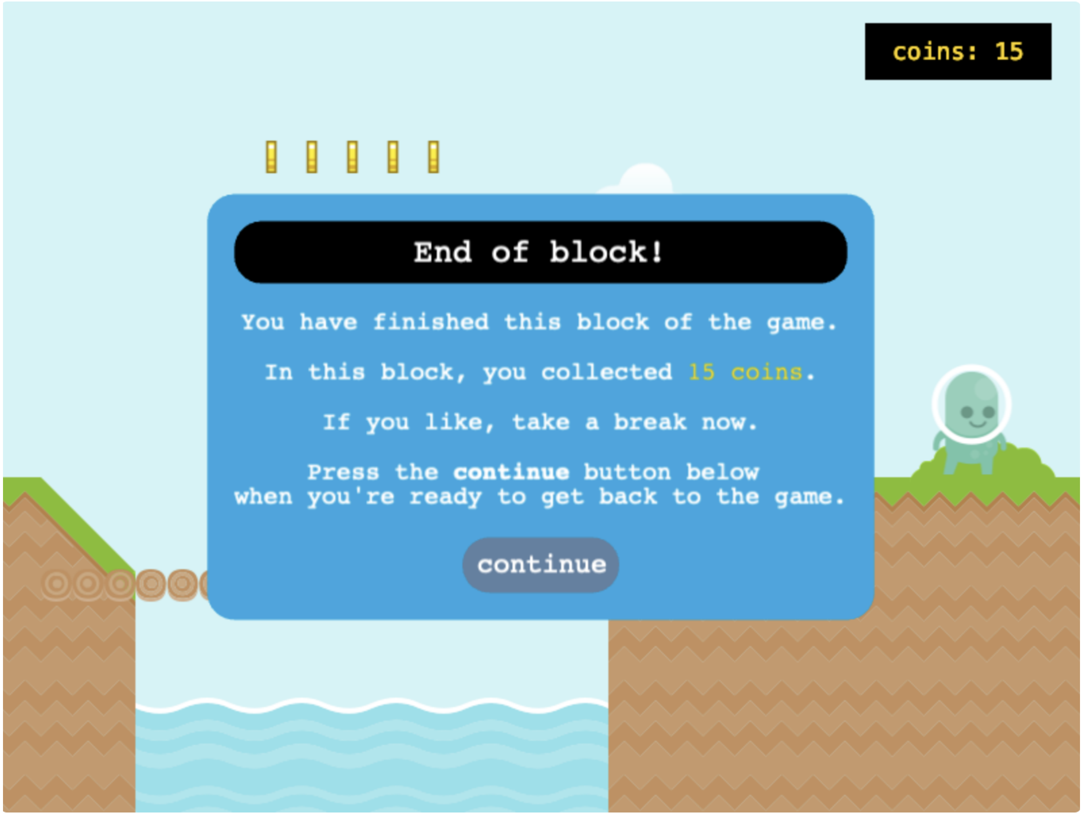
coins: 15
End of block!
You have finished this block of the game.
In this block, you collected 15 coins.
If you like, take a break now.
Press the continue button below
when you're ready to get back to the game.
continue

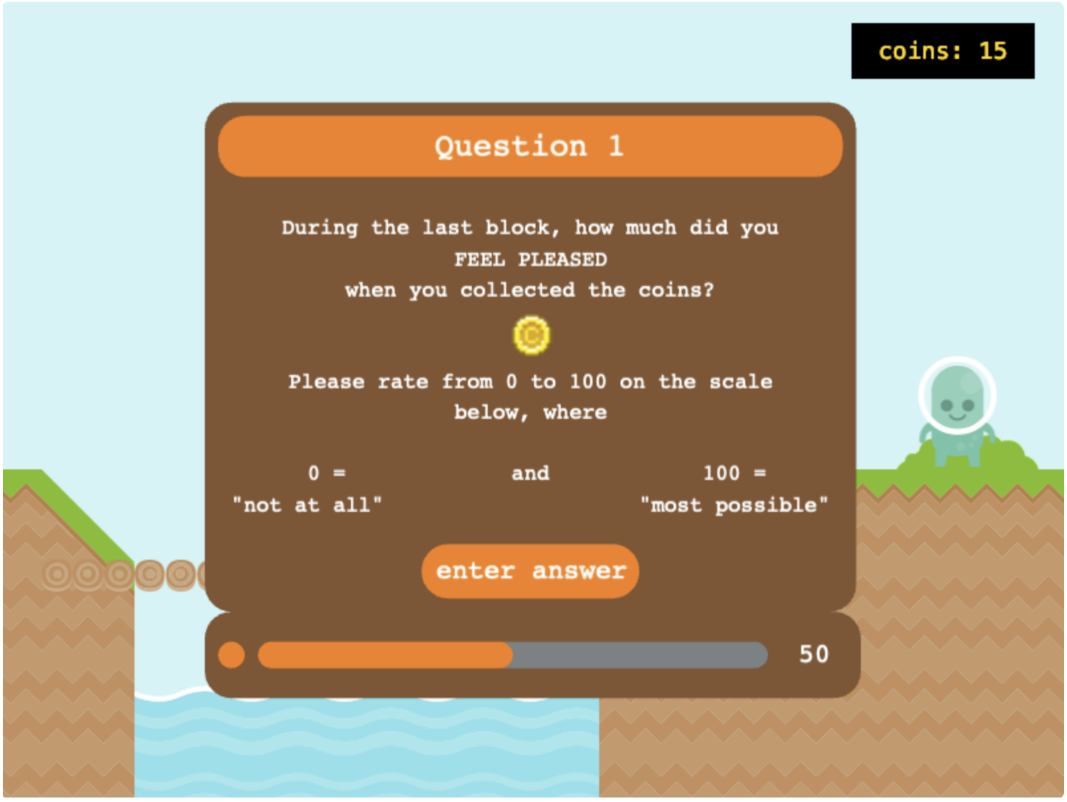
coins: 15
Question 1
During the last block, how much did you
FEEL PLEASED
when you collected the coins?
Please rate from 0 to 100 on the scale
below, where
0 =
"not at all"
and
100 =
"most possible"
enter answer
50

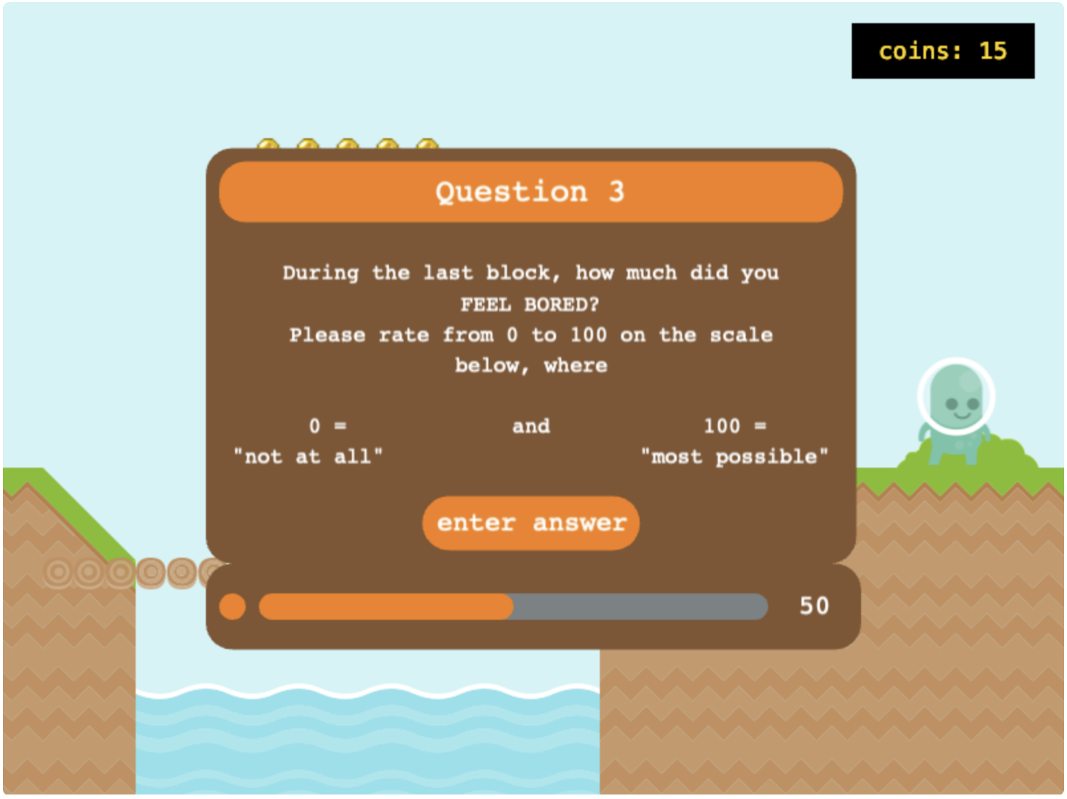
coins: 15
Question 3
During the last block, how much did you
FEEL BORED?
Please rate from 0 to 100 on the scale
below, where
0 =
"not at all"
and
100 =
"most possible"
enter answer
50

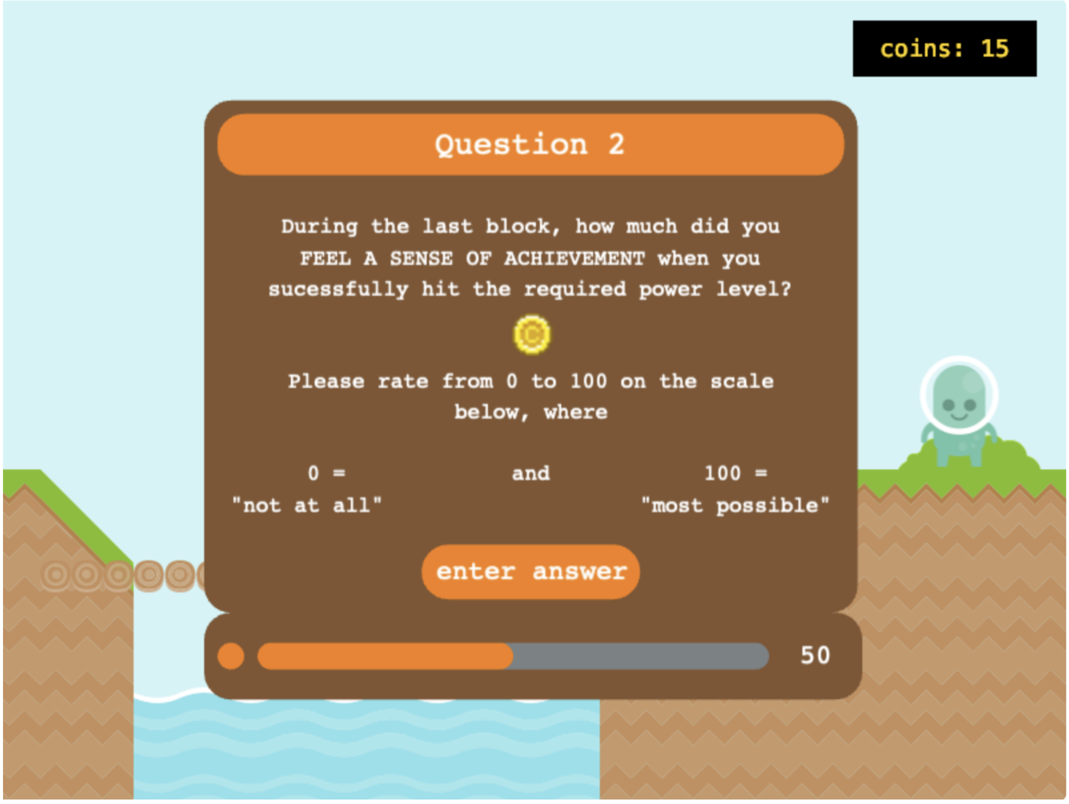
coins: 15
Question 2
During the last block, how much did you FEEL A SENSE OF ACHIEVEMENT when you sucessfully hit the required power level?
Please rate from 0 to 100 on the scale below, where
0 = "not at all"
and
100 = "most possible"
enter answer
50

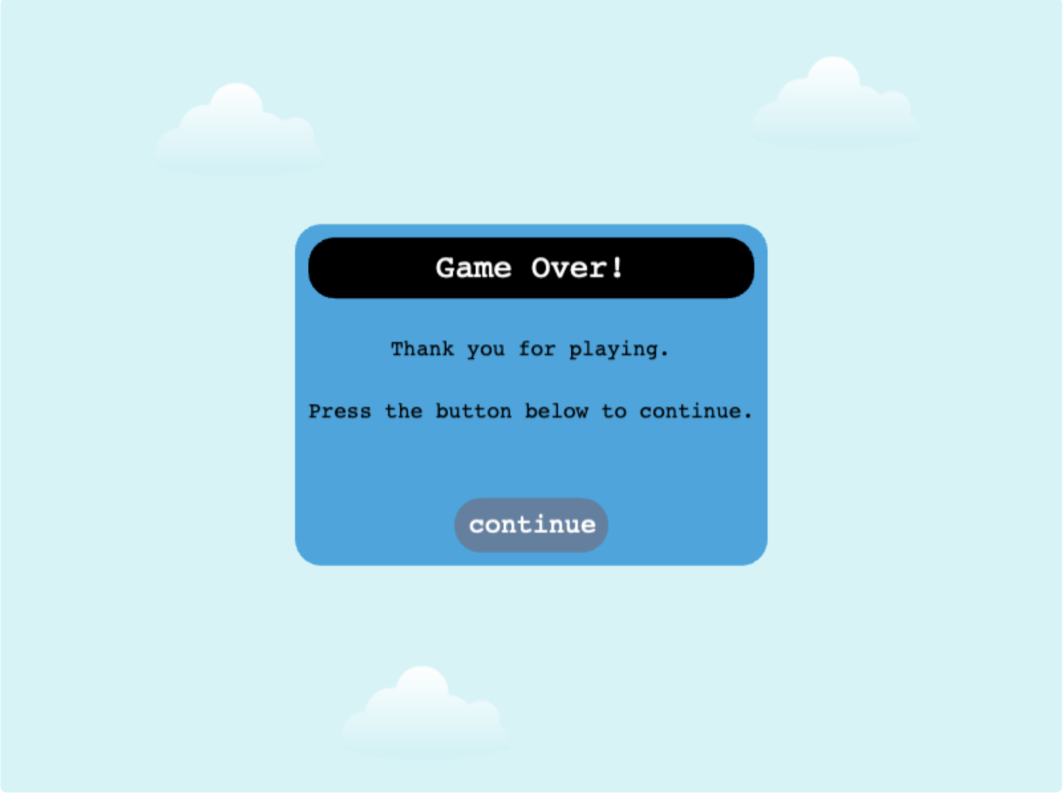
Game Over!
Thank you for playing.
Press the button below to continue.
continue

Intervention + Task 2 (goal-setting condition)

Introductory text:

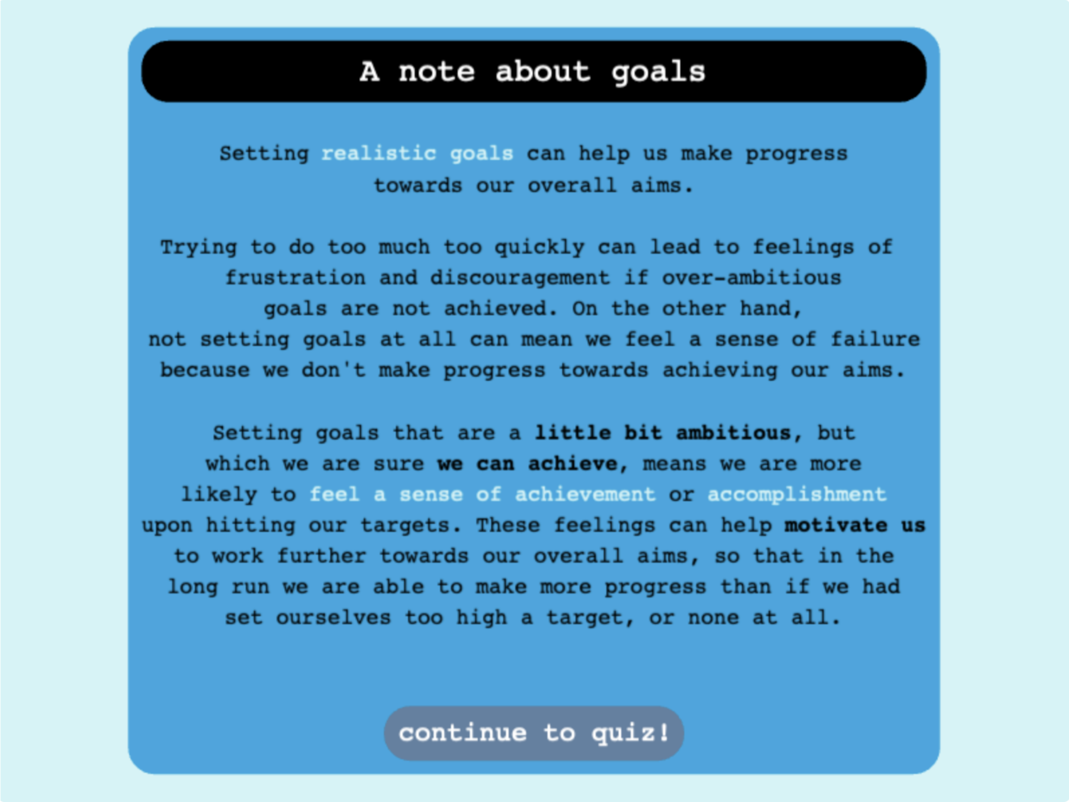

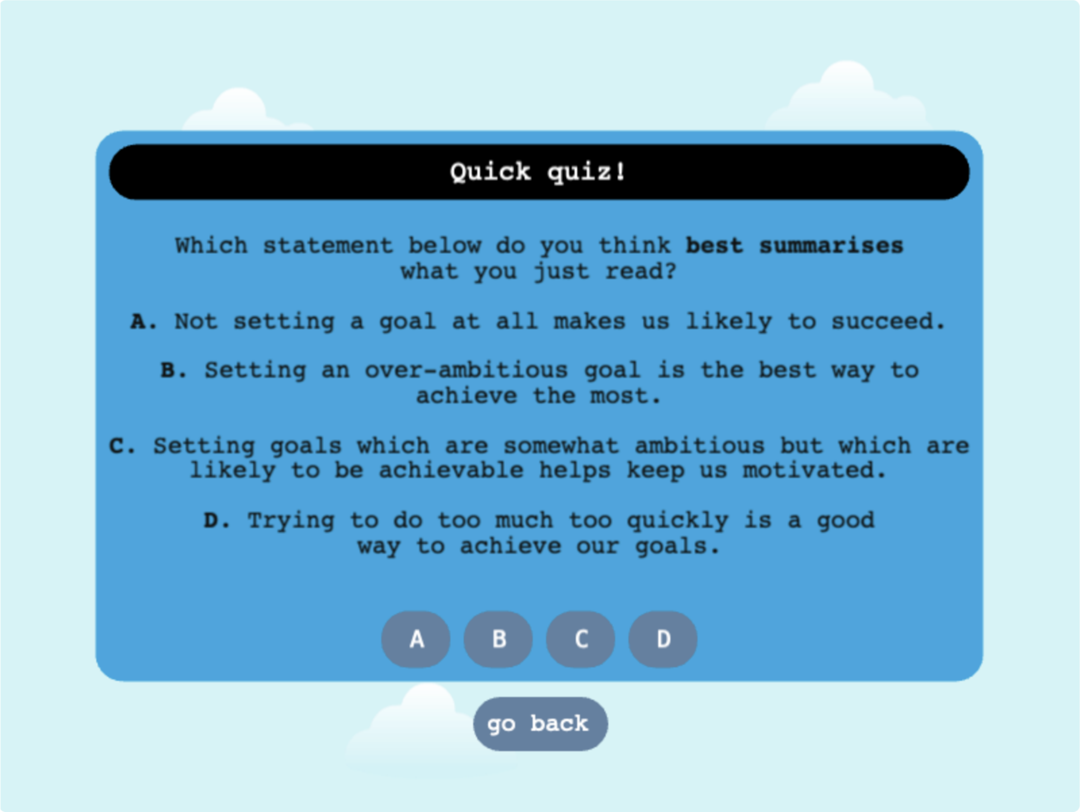

Task 2 goal-setting version:

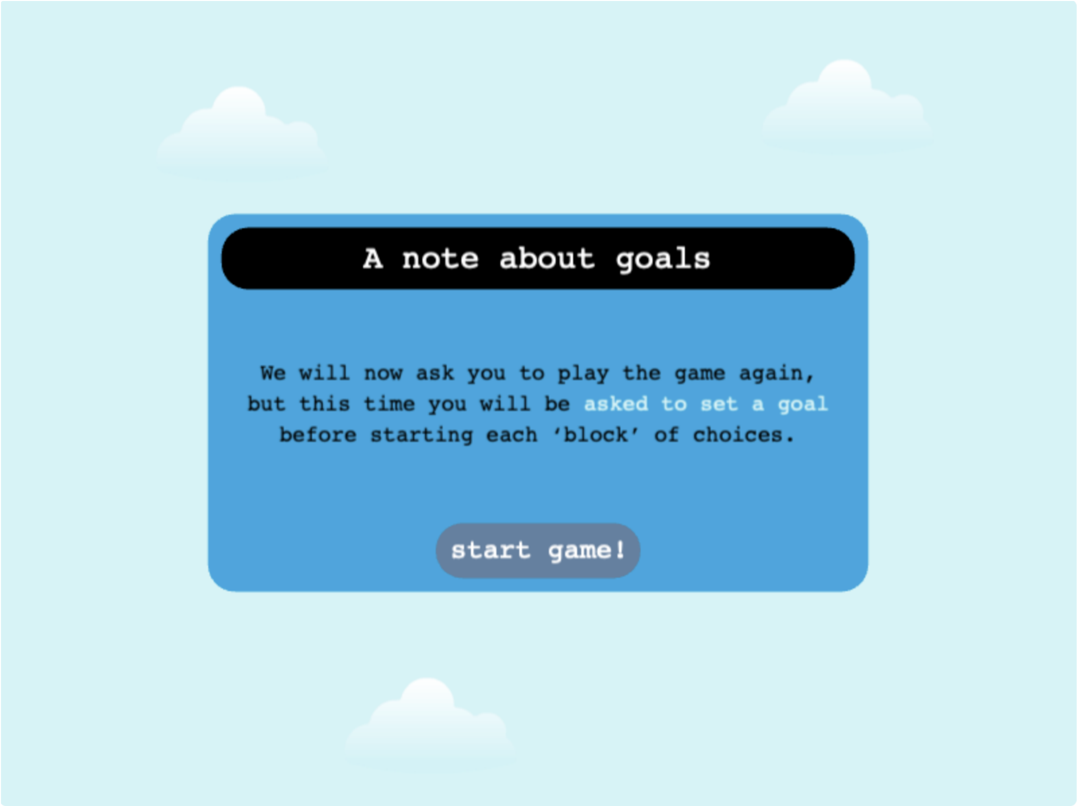

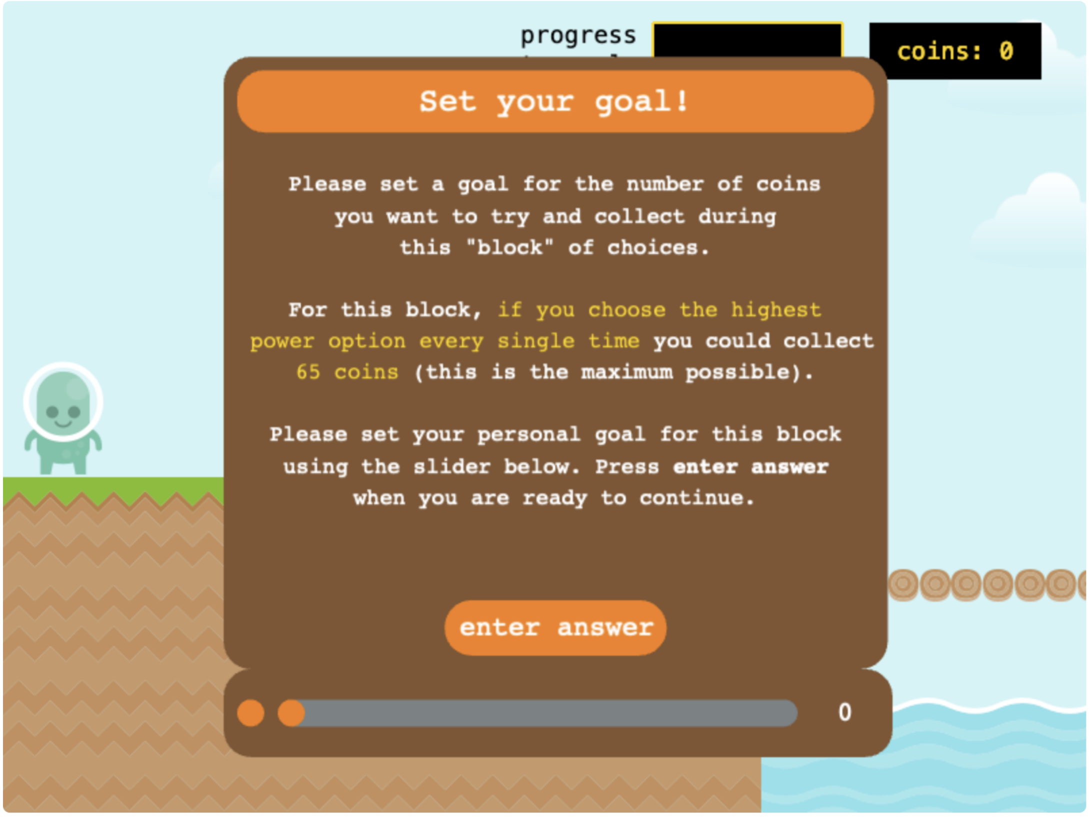

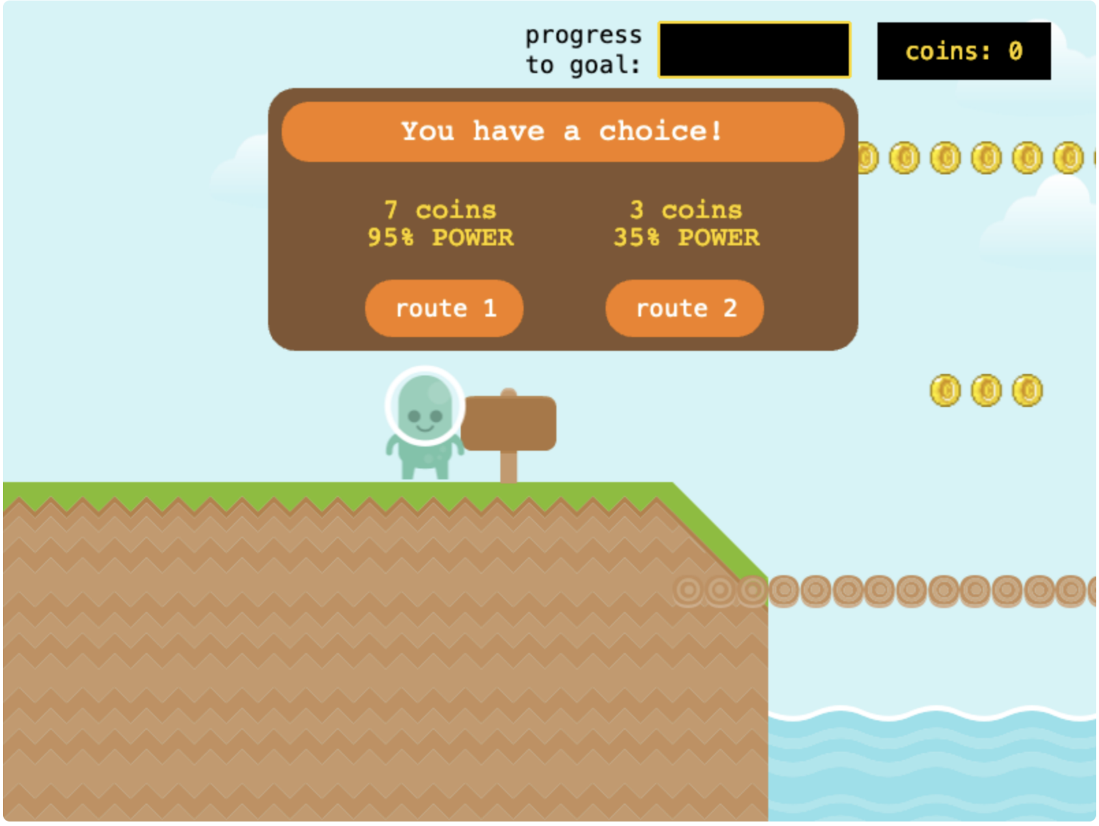
progress
to goal:
coins: 0
You have a choice!
7 coins
95% POWER
3 coins
35% POWER
route 1
route 2

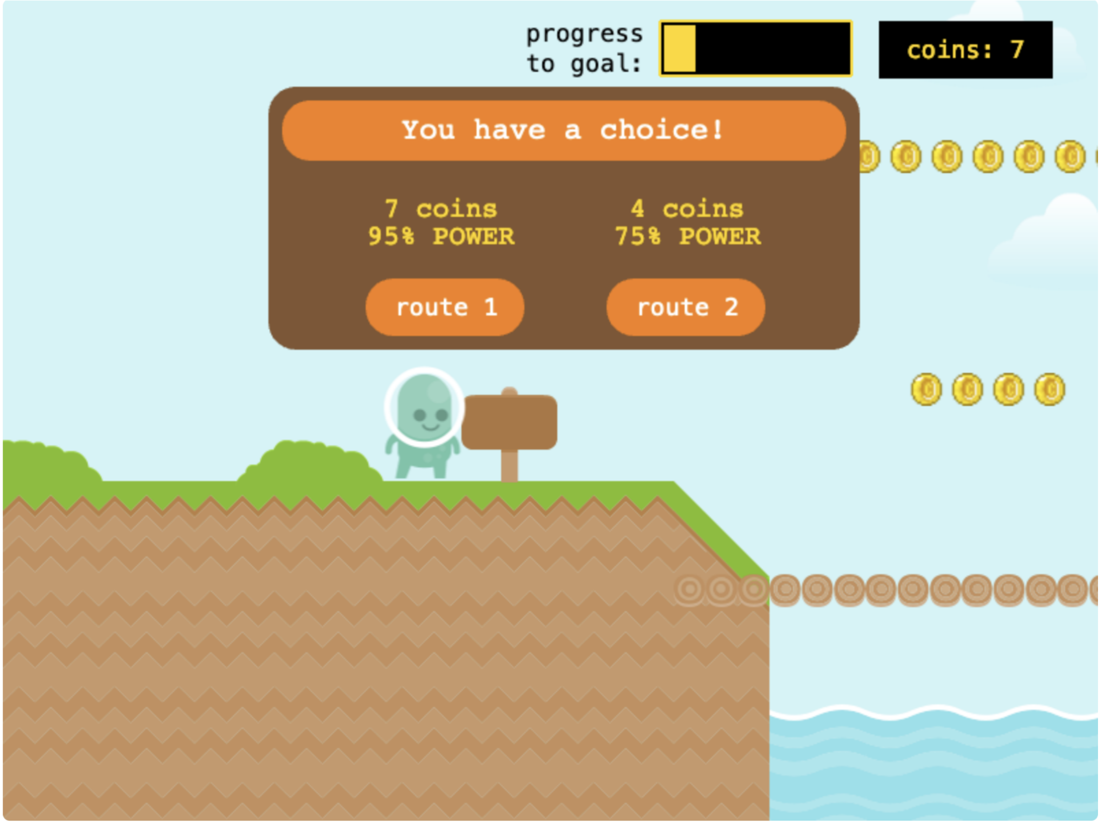
progress
to goal:
coins: 7
You have a choice!
7 coins
95% POWER
4 coins
75% POWER
route 1
route 2

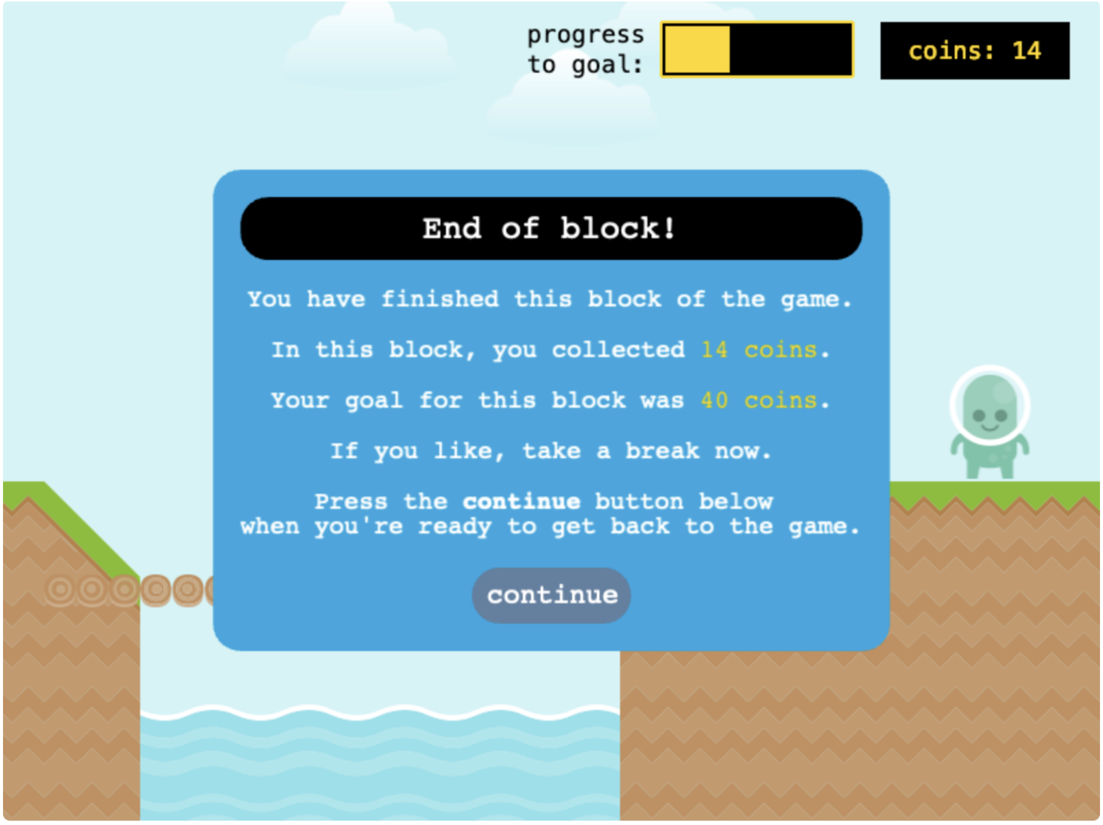

Intervention + Task 2 (control condition)

Introductory text:

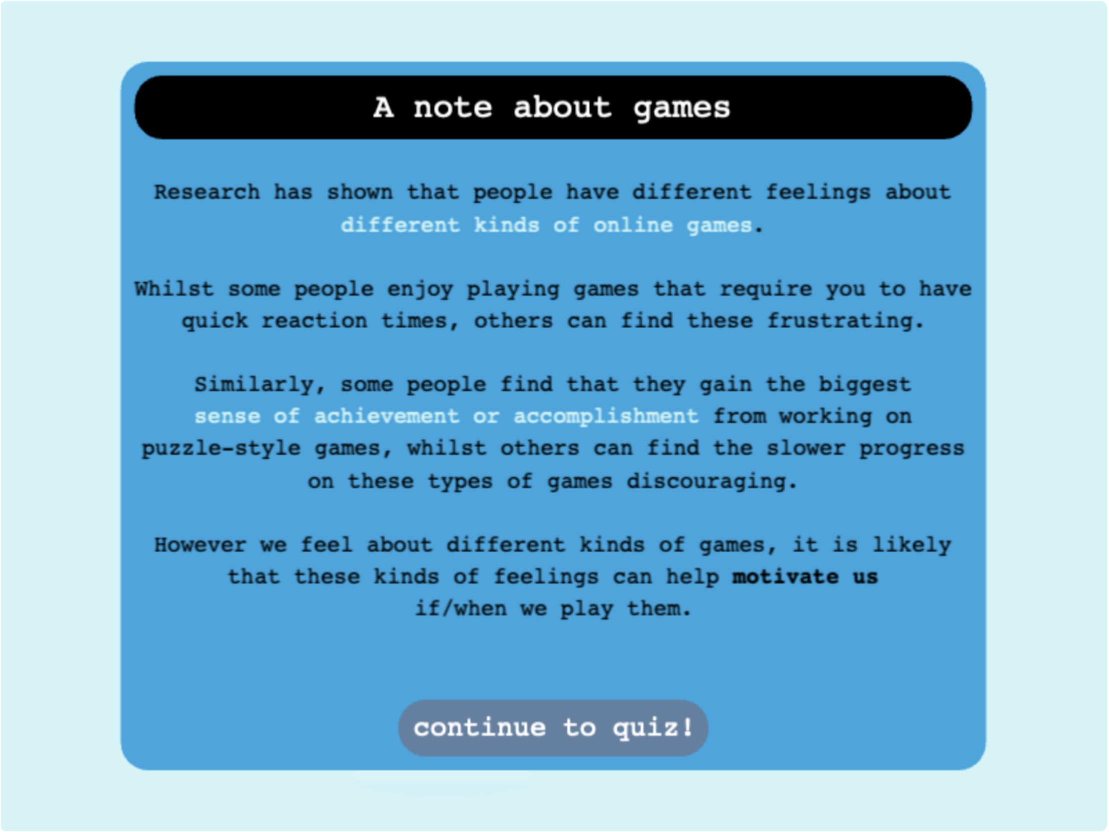

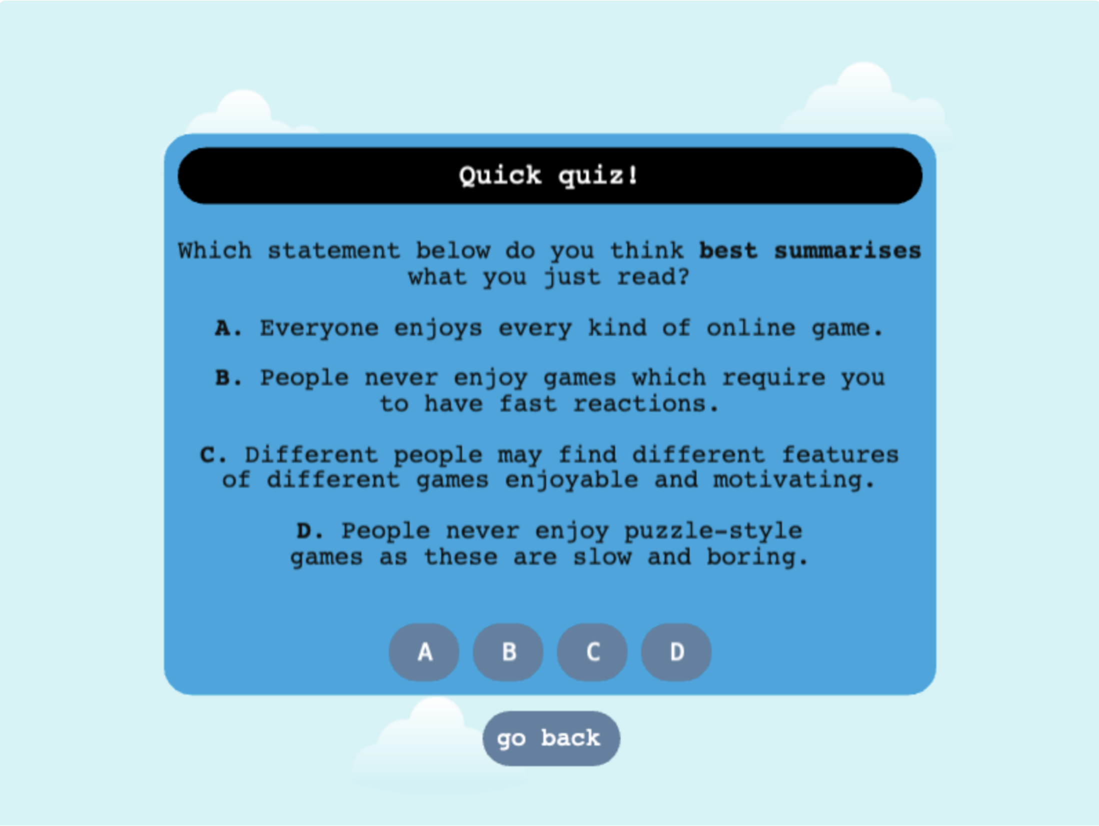

Task 2 control version:

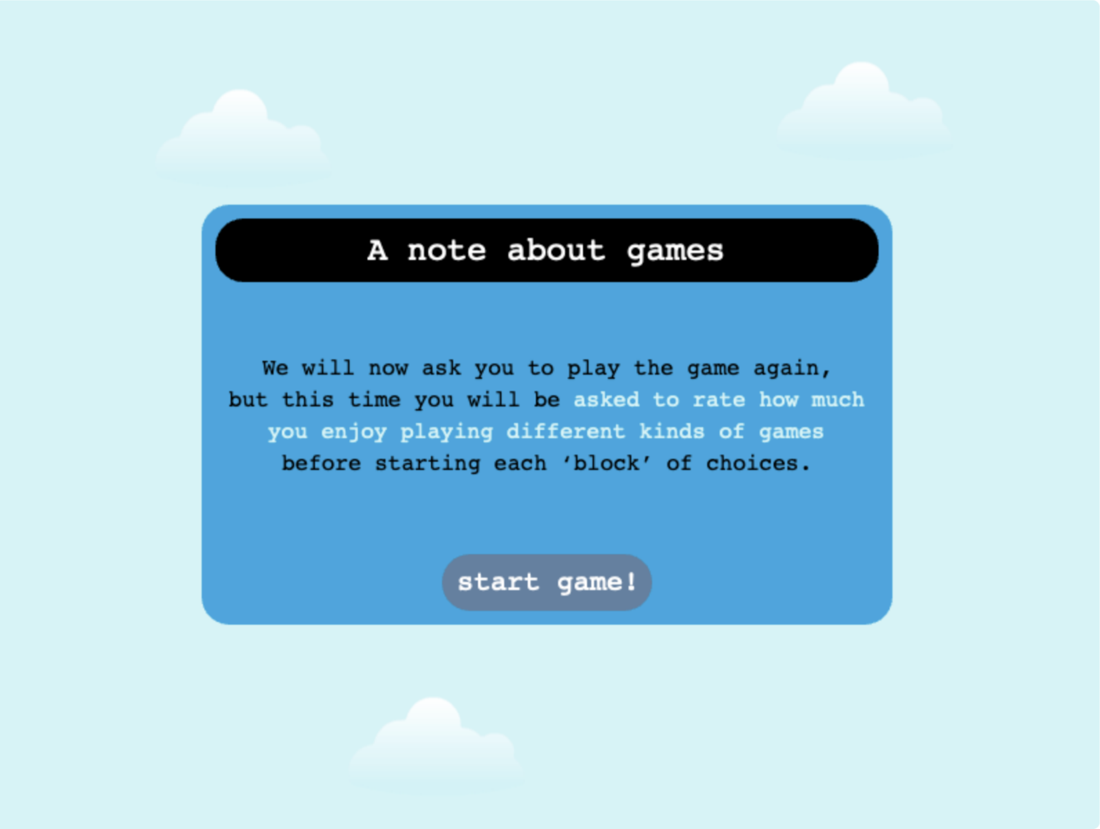

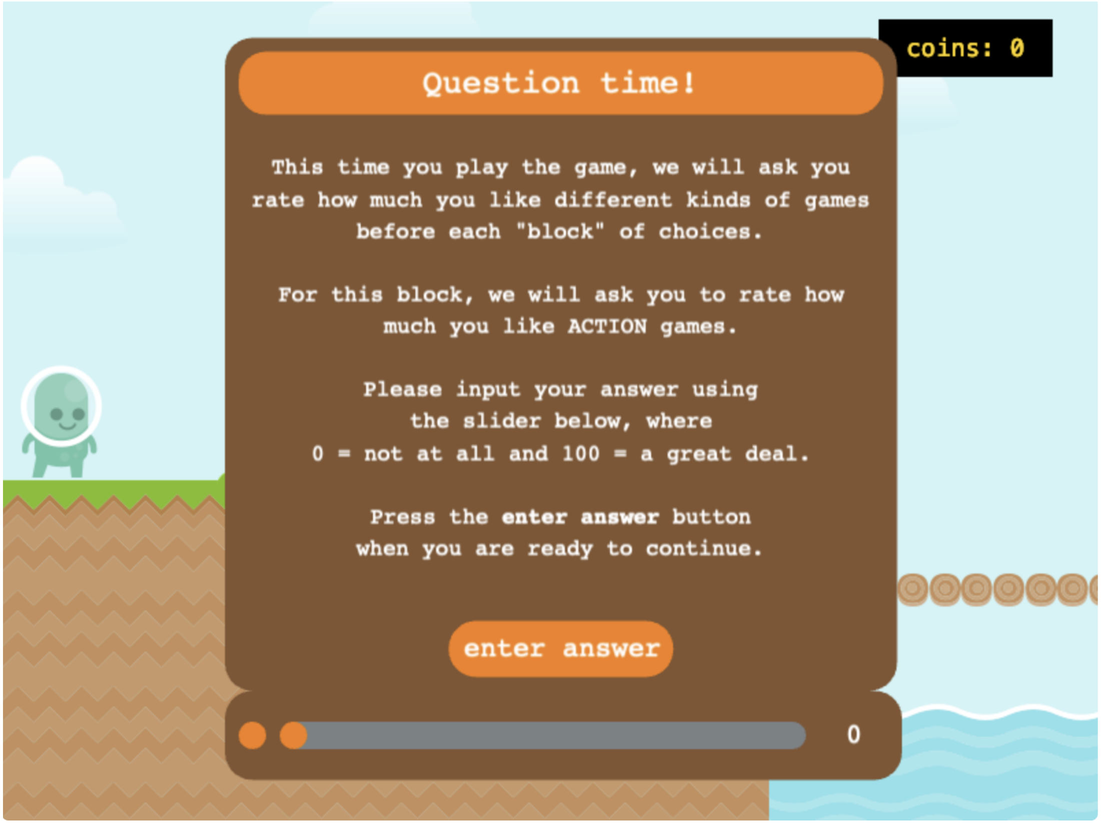
coins: 0
Question time!
This time you play the game, we will ask you rate how much you like different kinds of games before each "block" of choices.
For this block, we will ask you to rate how much you like ACTION games.
Please input your answer using the slider below, where 0 = not at all and 100 = a great deal.
Press the **enter answer** button when you are ready to continue.
enter answer
0

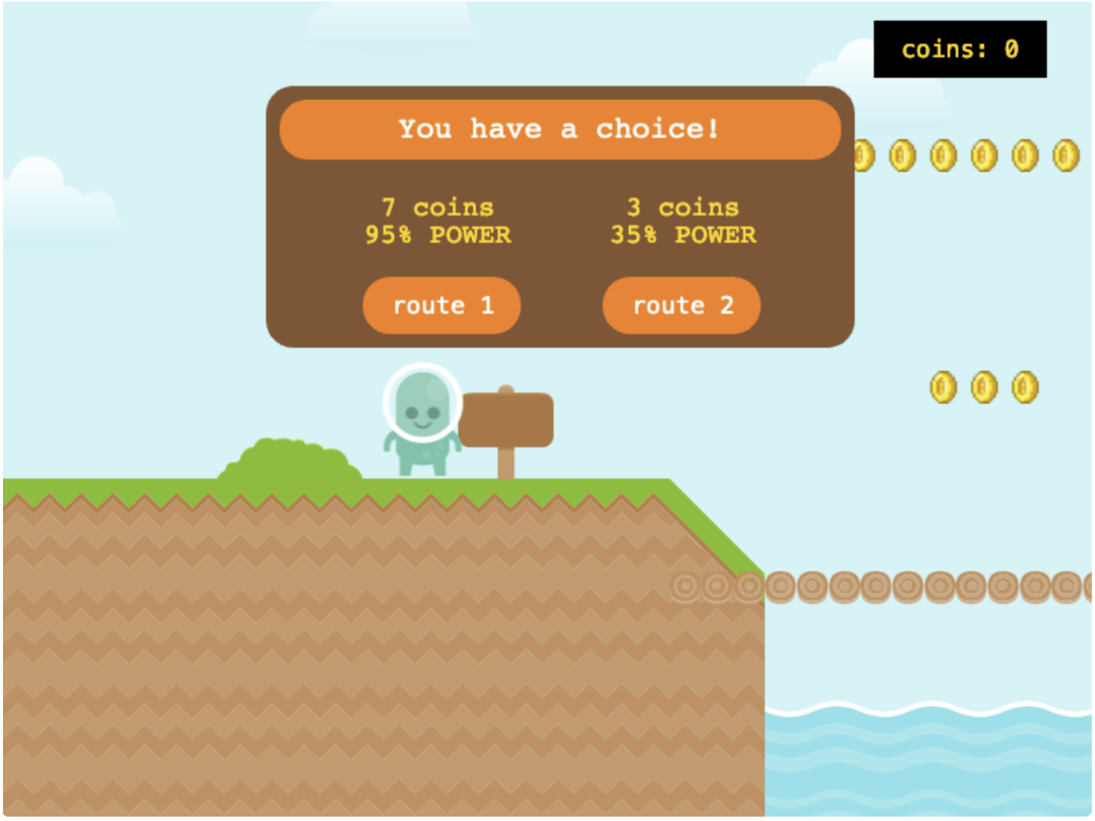
coins: 0
You have a choice!
7 coins
95% POWER
3 coins
35% POWER
route 1
route 2

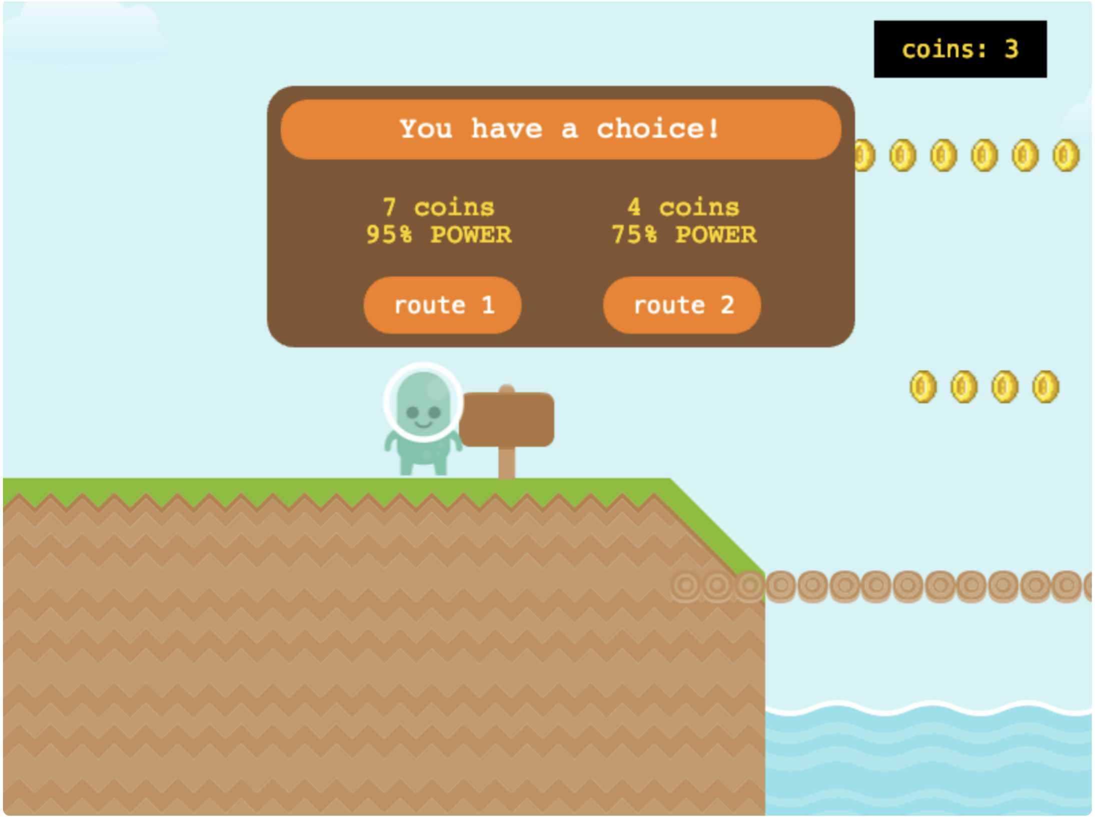
coins: 3
You have a choice!
7 coins
95% POWER
4 coins
75% POWER
route 1
route 2

Supplement 3. Full screenshots of the causal attribution task, and the cognitive restructuring and control interventions for causal attribution studies.

Causal-attribution studies

Task 1

Instructions:

Welcome to the study!

For this study, we will ask you to **imagine yourself in various different everyday situations**.

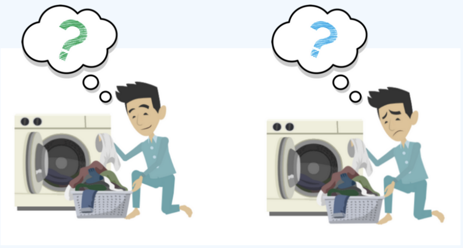

For each situation, we will ask you to choose *which of several possible explanations for what happened you think is the most likely*.

Although we know that events can have many different causes, we would like you to choose which explanation you think would be the **main reason** the event happened, **if it actually happened to *you***.

< back | next >

What do I need to do?

In order to do this, we will ask you to **first read the sentence at the top of each page.**

Picture the situation described as clearly as you can, as if the events were happening to you right now.

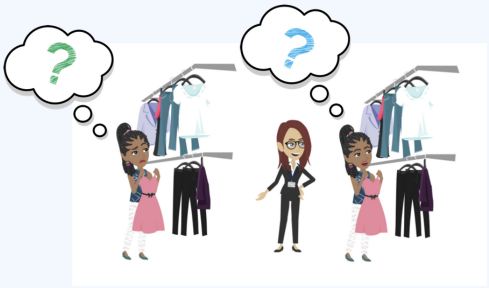

Your job is then to **click on one of the 'thought clouds'** below each statement, selecting which you think **best describes why the situation or event happened**.

< back | next >

Before you continue to the main part of the study, we will ask you to **answer some quick questions**. This is in order to make sure we have explained all the previous information clearly enough.

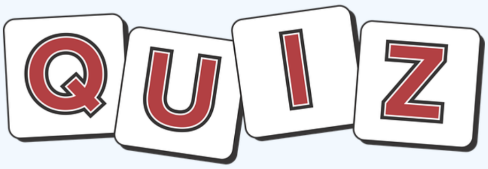

If you don't get all the questions right the first time, you will be routed back to the start of the instructions to try again. This helps us make sure everything is completely clear before we get started!

< back

next >

1. The point of the study is to...

A Answer each question according to how you think most people would explain the causes of different events

B Answer each question according to what you think would be the main cause behind an event, if it actually happened to you

C Answer each question completely at random, in order to generate unusable data

*

○ A ○ B ○ C

2. Events usually have multiple reasons behind them. For this study, I should...

A Select the main reason I think explains the events, from the options available

B Select all of the reasons I think are relevant to the event

C Type in my own answers

*

○ A ○ B ○ C

Main task:

You find out that someone you consider a friend has talked about you negatively behind your back

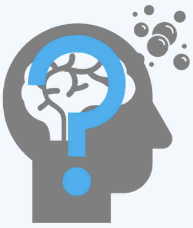

- Everyone has bad things said about them sometimes
- Deep down, my friends don't really like me
- My friend was probably just in a bad mood and letting off steam
- I probably did something recently to annoy them

Someone from work invites you out for a cup of coffee

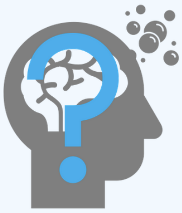

- I've made a particular effort to talk to this person recently
- People usually like to include me in things
- People at work generally reach out from time to time
- This person is probably trying to make a good impression for some reason

Intervention (restructuring condition)

Learning time!

Before you move on to the final part of the study, we would like you to give you some background information.

We hope that you will find this interesting.

continue >

Thoughts affect feelings: A core idea behind cognitive therapy

Some psychologists think that it is often the way people *interpret* events, rather than the events themselves, that leads to upsetting thoughts and feelings.

> For example, if a person has the belief "I'm a failure", they may interpret all negative things that happen to them as somehow resulting from their own actions, regardless of the actual reasons behind events.

This idea is helpful, because it explains why people experiencing the same event can react in different ways.

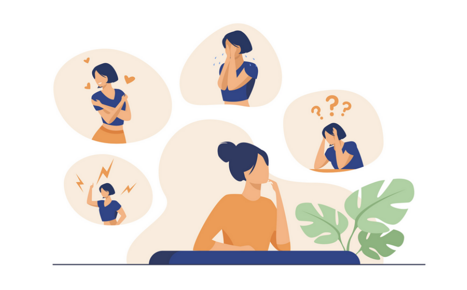

< back next >

Thoughts affect feelings: A core idea behind cognitive therapy

Over time, people who tend to interpret events in an unhelpful way may become more vulnerable to feeling low. This is because the upsetting thoughts and feelings may trigger a negative cycle of thoughts, feelings, and behaviour.

> For example, a person who believes they are a failure may give up trying to take part in activities that might lead to positive feelings (talking with friends or doing something they enjoy), as they already assume it will end up badly.

In cognitive therapy, this relationship between thoughts, feelings, and behaviours is known as the **cognitive triangle**.

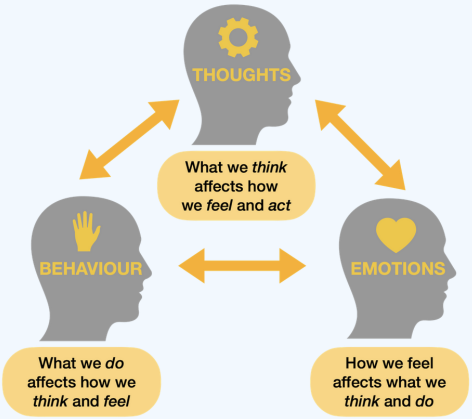

Thoughts affect feelings: A core idea behind cognitive therapy

One of the core ideas behind cognitive therapy is that it is possible to *identify* and then *challenge* unhelpful thoughts and beliefs.

Cognitive therapists believe that challenging unhelpful thoughts can change the way we interpret events, and therefore **change our emotional reactions to them**.

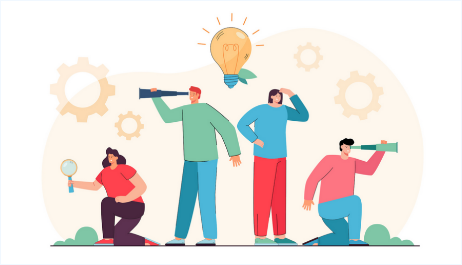

< back next >

Thoughts affect feelings: A core idea behind cognitive therapy

Let's walk through some examples to see how this might work in practice.

< back   next >

Thoughts affect feelings: A core idea behind cognitive therapy

Example one

> Imagine a friend walks past you without acknowledging you

Below are two different ways you could think about this event.

Interpretation 1	Interpretation 2
"I must have done something to upset them"	"They probably just didn't see me"

What kind of feelings do you think you might experience if you thought interpretation 1 was true?

☐ worried ☐ tense ☐ relaxed ☐ unconcerned

What kind of feelings do you think you might experience if you thought interpretation 2 was true?

☐ worried ☐ tense ☐ relaxed ☐ unconcerned

Which interpretation do you think is the most helpful to think about?

Choose...

continue

Thoughts affect feelings: A core idea behind cognitive therapy

Example two

> Imagine your boss tells you they have decided to offer you a promotion

Below are two different ways you could think about this event.

Interpretation 1

"I've worked hard and must have done well this year"

Interpretation 2

"They probably feel they have to offer this to everyone after a certain amount of time"

What kind of feelings do you think you might experience if you thought interpretation 1 was true?

☐ proud ☐ excited ☐ neutral ☐ nervous

What kind of feelings do you think you might experience if you thought interpretation 2 was true?

☐ proud ☐ excited ☐ neutral ☐ nervous

Which interpretation do you think is the most helpful to think about?

Choose...

continue

Thoughts affect feelings: A core idea behind cognitive therapy

Your turn!

> Think of something *negative* that happened to you recently. For example, something that didn't go the way you would have liked it to at work, or in a social situation. What was going through your mind in that moment?

Briefly, summarise your first thoughts about why this event happened

Now, try and come up with an alternative interpretation. Is there another way of seeing things?

Consider how you would feel after thinking about each of the two different interpretations. Which explanation do you think is the most helpful to think about?

Choose...

Which explanation do you think would be more likely to be true if this event happened to a colleague or friend of yours?

Choose...

If you were looking back on this event from 20 years in the future, which interpretation do you think you would be likely to choose then?

Choose...

continue

Thoughts affect feelings: A core idea behind cognitive therapy

Your turn!

> Now, think of something *positive* that happened to you recently. For example, something that went well at work, socially, or when taking part in another activity. What was going through your mind in that moment?

Briefly, summarise your first thoughts about why this event happened

Now, try and come up with an alternative interpretation. Is there another way of seeing things?

Consider how you would feel after thinking about each of the two different interpretations. Which explanation do you think is the most helpful to think about?

Choose...

Which explanation do you think would be more likely to be true if this event happened to a colleague or friend of yours?

Choose...

If you were looking back on this event from 20 years in the future, which interpretation do you think you would be likely to choose then?

Choose...

continue

Thoughts affect feelings: A core idea behind cognitive therapy

Putting it all together

To finish up this part of the study, we'd like to ask you some questions about the information you've just read.

Please note, these questions are just to try and understand how clearly we have explained the material - your answers will not affect the chance of your submission being approved, and you will continue on to the final main part of the study at the end, whichever options you choose.

For each group of sentences below, which best summarises the information you just read?

1

- ○ Thoughts are facts. The way we think about and interpret events is always correct.
- ○ People can vary in their interpretations of the same events.
- ○ People always interpret negative events in negative ways

2

- ○ What we think doesn't make any difference to how we feel and act.
- ○ Different people experiencing the same events always experience the same feelings.
- ○ How we interpret events can affect our emotional responses to them.

3

- ○ Whatever we think about events, we will always feel generally the same in the future as we do now.
- ○ If we can learn to challenge unhelpful thoughts about events, over time we may come to experience less upsetting reactions to them.

continue

Intervention (control condition)

Learning time!

Before you move on to the final part of the study, we would like you to give you some background information.

We hope that you will find this interesting.

continue >

Emotions as signals: a core idea behind emotion-focused therapy

Emotions are one of the most complex biological processes in the human body.

> The term dates back to the 1500s and comes from the French word *emouvoir*, which means "to stir up".

Emotions are powerful experiences. They can be both positive and negative, and both positive and negative emotions are experienced by all of us in our daily lives. Despite - or perhaps because of - this, many psychologists believe that emotions are an essential aspect of being human.

Emotion-focused therapy centers the role of emotions in human behaviour. Emotion-focused therapists believe that emotions function both to tell people *what the problem is*, and keep them motivated to *do something about it*.

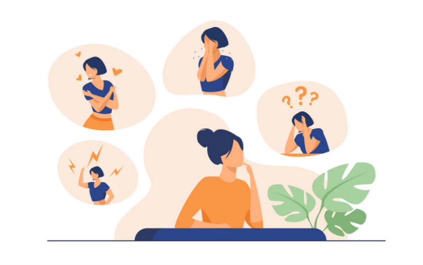

< back next >

Emotions as signals: a core idea behind emotion-focused therapy

In particular, emotion-focused therapists argue that emotions are *signals*. They offer messages that you are in danger, that your boundaries are being crossed, that a safe and familiar person is absent, or that you are close to someone safe and familiar.

> Emotion-focused therapy argues that rather than attempting to control, interrupt, change, or avoid the experience of emotions, people need to learn to live in harmony with them.

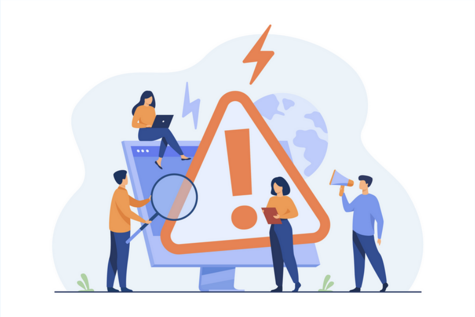

< back next >

Emotions as signals: a core idea behind emotion-focused therapy

People can start to incorporate an emotion-focused approach into their daily lives by attending to their bodies and learning to recognize and **label** what they feel. They can first acknowledge these labels to themselves, and then, when appropriate, to others.

Having acknowledged and labelled their emotions, people can then begin to **understand** these feelings. To do this, people have to 'use their heads' to make sense of their experiences, by working out what their feelings and emotions might be trying to signal to them.

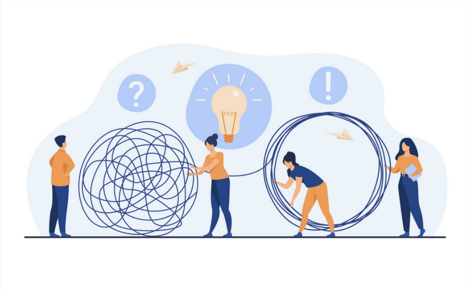

< back next >

Emotions as signals: a core idea behind emotion-focused therapy

Let's walk through some examples to see how this might work in practice.

< back next >

Emotions as signals: a core idea behind emotion-focused therapy

Example one

> Imagine you are away on a trip and your partner tells you they are enjoying the time alone

A reasonable response to hearing this may be to initially withdraw into yourself and think "I know they mean well, but hearing this makes me feel rejected."

From an emotion-focused perspective, the first step with dealing with this situation would be to **recognise and label the emotions you are experiencing.**

Which of the following do you think you might feel in this situation?

☐ sad ☐ lonely ☐ embarrassed ☐ relieved

What signal or message do you think these feelings might be trying to convey?

- ◯ you are in physical danger
- ◯ an important relationship may be at risk
- ◯ you are close to someone safe and familiar

How do you think you might be likely to respond to this signal?

- ◯ hang up the phone and leave the room
- ◯ say to your partner "When you sound so enthusiastic about me being away, I feel pushed away"
- ◯ say to your partner "I'm also really happy right now"

continue

Emotions as signals: a core idea behind emotion-focused therapy

Example one

> Imagine you receive a message from a close friend saying that they are thinking of you

Which of the following do you think you might feel at this point in time?

☐ angry ☐ upset ☐ content ☐ secure

What signal or message do you think these feelings might be trying to convey?

○ your boundaries are being crossed
○ an important relationship may be at risk
○ you are close to someone safe and familiar

How do you think you might respond to this signal?

○ put down your phone and don't respond
○ reply to your friend "I'm feeling rejected right now"
○ reply to your friend "When you message, I feel close to you"

continue

Emotions as signals: a core idea behind emotion-focused therapy

Your turn!

> Bring to mind a challenge that you are facing at the moment, perhaps at work or at home.

Observe any emotions or bodily sensations that come to mind when thinking about this particular challenge.

Briefly describe these feelings below.

What do you think these emotions might be trying to signal to you?

Is it easy to understand the message behind these feelings?

Choose...

continue

Emotions as signals: a core idea behind emotion-focused therapy

Your turn!

> Now, bring to mind something you think is going well in your life at the moment - perhaps at work, in a relationship, or a hobby you enjoy.

Observe any emotions or bodily sensations that come to mind when thinking about this particular situation.

Briefly describe these feelings below.

What do you think these emotions might be trying to signal to you?

Is it easy to understand the message behind these feelings?

Choose...

continue

Emotions as signals: a core idea behind emotion-focused therapy

Putting it all together

To finish up this part of the study, we'd like to ask you some questions about the information you've just read.

Please note, these questions are just to try and understand how clearly we have explained the material - your answers will not affect the chance of your submission being approved, and you will continue on to the final main part of the study at the end, whichever options you choose.

For each group of sentences below, which best summarises the information you just read?

1

- ◯ The first step of an emotionally-informed response to a situation is to recognise and then discount whatever emotions you are feeling
- ◯ The first step of an emotionally-informed response to a situation is to recognise and label what emotions you are feeling.
- ◯ The first step of an emotionally-informed response to a situation is to ignore whatever emotions you are feeling.

2

- ◯ The second step of an emotionally-informed response to a situation is to walk away from the situation to take a breath.
- ◯ The second step of an emotionally-informed response to a situation is to dive straight into whatever your gut tells you to do.
- ◯ The second step of an emotionally-informed response to a situation is to try and work out what signal these feelings are trying to convey.

3

- ◯ In general, emotions and feelings can get in the way when trying to make sense of our experiences, so we should try to change or control them.
- ◯ In general, the messages or signals conveyed by emotions are important, so we should try to learn to live in harmony with them.

continue

Task 2

Last main part of the study!

For the final main part of the study, we would like you to ask you to think about **a new set of events**. (This is the last time we will ask you do this!)

Like the first part of the study, we would like you to read the description of each event, then ***select the main reason you think that event would have happened, if it actually happened to you***.

For this part of the study, there are **no right or wrong answers**. All we ask if that you try and choose the answer that *most accurately reflects the main reason you think would be behind the event, if it happened to you right now.*

We will again ask you to think about 32 different events. Like the first part of the study, we will let you know when you are half-way through, and you can take a short break then if you like.

Please press the **continue** button when you are ready to start!

continue

A colleague tells you they like your company

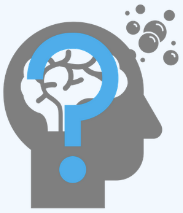

Colleages tend to be polite about their co-workers

This particular colleague probably wants me to do something for them

I seem to click well with this particular person

I'm a fun person to be around

You discover that you haven't been invited to an after-work social event

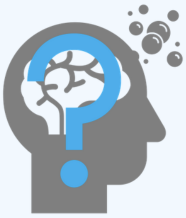

I haven't shown any interest in work events recently

Everyone forgets to include someone sometimes

My colleagues probably thought I was too busy at the time

I'm not enjoyable to have around

REFERENCES AND NOTES

1. P. Cuijpers, I. A. Cristea, E. Karyotaki, M. Reijnders, M. J. Huibers, How effective are cognitive behavior therapies for major depression and anxiety disorders? A meta-analytic update of the evidence. *World Psychiatry* **15**, 245–258 (2016).

2. P. Cuijpers, E. Karyotaki, M. Reijnders, M. J. H. Huibers, Who benefits from psychotherapies for adult depression? A meta-analytic update of the evidence. *Cogn. Behav. Ther.* **47**, 91–106 (2018).

3. E. A. M. van Dis, S. C. van Veen, M. A. Hagenaars, N. M. Batelaan, C. L. H. Bockting, R. M. van den Heuvel, P. Cuijpers, I. M. Engelhard, Long-term outcomes of cognitive behavioral therapy for anxiety-related disorders: A systematic review and meta-analysis. *JAMA Psychiatry* **77**, 265–273 (2020).

4. A. E. Kazdin, Understanding how and why psychotherapy leads to change. *Psychother. Res.* **19**, 418–428 (2009).

5. E. A. Holmes, M. G. Craske, A. M. Graybiel, Psychological treatments: A call for mental-health science. *Nature* **511**, 287–289 (2014).

6. A. E. Kazdin, S. L. Blase, Rebooting psychotherapy research and practice to reduce the burden of Mental Illness. *Perspect. Psychol. Sci.* **6**, 21–37 (2011).

7. M. J. H. Huibers, L. Lorenzo-Luaces, P. Cuijpers, N. Kazantzis, On the road to personalized psychotherapy: A research agenda based on cognitive behavior therapy for depression. *Front. Psych.* **11**, (2021).

8. M. Moutoussis, N. Shahar, T. U. Hauser, R. J. Dolan, Computation in psychotherapy, or how computational psychiatry can aid learning-based psychological therapies. *Computational Psychiatry* **2**, 50–73 (2020).

9. A. M. Reiter, N. A. Atiya, I. M. Berwian, Q. J. Huys, Neuro-cognitive processes as mediators of psychological treatment effects. *Curr. Opin. Behav. Sci.* **38**, 103–109 (2021).

10. Q. J. M. Huys, E. M. Russek, G. Abitante, T. Kahnt, J. K. Gollan, Components of behavioral activation therapy for depression engage specific reinforcement learning mechanisms in a pilot study. *Computational Psychiatry* **6**, 238–255 (2022).

11. Q. Dercon, S. Z. Mehrhof, T. R. Sandhu, C. Hitchcock, R. P. Lawson, D. A. Pizzagalli, T. Dalgleish, C. L. Nord, A core component of psychological therapy causes adaptive changes in computational learning mechanisms. *Psychol. Med.*, **1–11** (2023).

12. E. A. Holmes, A. Ghaderi, C. J. Harmer, P. G. Ramchandani, P. Cuijpers, A. P. Morrison, J. P. Roiser, C. L. H. Bockting, R. C. O'Connor, R. Shafran, M. L. Moulds, M. G. Craske, The lancet psychiatry commission on psychological treatments research in tomorrow's science. *Lancet Psychiatry* **5**, 237–286 (2018).

13. Wellcome, What science has shown can help young people with anxiety and depression. Identifying and reviewing the 'active ingredients' of effective interventions: Part 1, *Tech. rep.* (2021).

14. D. M. Clark, Realizing the mass public benefit of evidence-based psychological therapies: The IAPT program. *Annu. Rev. Clin. Psychol.* **14**, 159–183 (2018).

15. A. T. Beck, A. J. Rush, B. F. Shaw, G. Emery, *Cognitive Therapy of Depression* (Guildford Press, 1987).

16. C. R. Martell, S. Dimidjian, R. Herman-Dunn, *Behavioral Activation for Depression: A Clinician's Guide* (Guilford Press, 2013).

17. A. Gelman, J. B. Carlin, H. S. Stern, D. B. Rubin, *Bayesian Data Analysis* (Chapman and Hall/CRC, 1995).

18. T. L. Griffiths, C. Kemp, J. B. Tenenbaum, *Bayesian Models of Cognition* (Cambridge Univ Press, Cambridge, 2008).

19. K. Katahira, How hierarchical models improve point estimates of model parameters at the individual level. *J. Math. Psychol.* **73**, 37–58 (2016).

20. J. N. Rouder, J. M. Haaf, A psychometrics of individual differences in experimental tasks. *Psychon. Bull. Rev.* **26**, 452–467 (2019).

21. N. Haines, P. D. Kvam, L. H. Irving, C. Smith, T. P. Beauchaine, M. A. Pitt, W.-Y. Ahn, B. M. Turner, Theoretically Informed Generative Models Can Advance the Psychological and Brain Sciences: Lessons from the Reliability Paradox, https://psyarxiv.com/xr7y3/ (2020).

22. V. M. Brown, J. Chen, C. M. Gillan, R. B. Price, Improving the reliability of computational analyses: Model-based planning and its relationship with compulsivity. *Biol. Psychiatry: Cogn. Neurosci. Neuroimaging.* **5**, 601–609 (2020).

23. S. Talts, M. Betancourt, D. Simpson, A. Vehtari, A. Gelman, Validating Bayesian Inference Algorithms with Simulation-Based Calibration, http://arxiv.org/abs/1804.06788 (2020). ArXiv:1804.06788 [stat].

24. D. J. Schad, M. Betancourt, S. Vasishth, Toward a principled Bayesian workflow in cognitive science. *Psychol. Methods* **26**, 103–126 (2021).

25. C. Hedge, A. Bompas, P. Sumner, Task reliability considerations in computational psychiatry. *Biol. Psychiatry: Cogn. Neurosci. Neuroimaging.* **5**, 837–839 (2020).

26. S. Zorowitz, Y. Niv, Improving the reliability of cognitive task measures: A narrative review. *Biol. Psychiatry: Cogn. Neurosci. Neuroimaging.* **8**, 789–797 (2023).

27. A. K. Graham, E. G. Lattie, D. C. Mohr, Experimental therapeutics for digital mental health. *JAMA Psychiatry* **76**, 1223–1224 (2019).

28. C. T. Lee, J. Palacios, D. Richards, A. K. Hanlon, K. Lynch, S. Harty, N. Claus, L. Swords, V. O'Keane, K. E. Stephan, C. M. Gillan, The Precision in Psychiatry (PIP) study: Testing an internet-based methodology for accelerating research in treatment prediction and personalisation. *BMC Psychiatry* **23**, 25 (2023).

29. C. Cheng, O. V. Ebrahimi, A meta-analytic review of gamified interventions in mental health enhancement. *Computers in Human Behavior* **141**, 107621 (2023).

30. B. Long, J. Simson, A. Buxo-Lugo, D. G. Watson, S. A. Mehr, How games can make ´ behavioural science better. *Nature* **613**, 433–436 (2023).

31. S. Singh, R. W. Strong, L. Jung, F. H. Li, L. Grinspoon, L. S. Scheuer, E. J. Passell, P. Martini, N. Chaytor, J. R. Soble, L. Germine, The TestMyBrain digital neuropsychology toolkit: Development and psychometric characteristics. *J. Clin. Exp. Neuropsychol.* **43**, 786–795 (2021).

32. M. P. Paulus, Q. J. M. Huys, T. V. Maia, A roadmap for the development of applied computational psychiatry. *Biol. Psychiatry: Cogn. Neurosci. Neuroimaging.* **1**, 386–392 (2016).

33. L. Quigley, K. S. Dobson, Chapter 12 - Behavioral Activation Treatments for Depression. The Science of Cognitive Behavioral Therapy, S. G. Hofmann, G. J. G. Asmundson, eds. (Academic Press, San Diego, 2017), pp. 291–318.

34. M. T. Treadway, J. W. Buckholtz, A. N. Schwartzman, W. E. Lambert, D. H. Zald, Worth the 'EEfRT'? the effort expenditure for rewards task as an objective measure of motivation and anhedonia. *PLOS One* **4**, e6598 (2009).

35. V. Bonnelle, K.-R. Veromann, S. Burnett Heyes, E. Lo Sterzo, S. Manohar, M. Husain, Characterization of reward and effort mechanisms in apathy. *J. Physiol. Paris.* **109**, 16–26 (2015).

36. I. M. Berwian, J. G. Wenzel, A. G. E. Collins, E. Seifritz, K. E. Stephan, H. Walter, Q. J. M. Huys, Computational mechanisms of effort and reward decisions in patients with depression and their association with relapse after antidepressant discontinuation. *JAMA Psychiatry* **77**, 513–522 (2020).

37. T. Wise, R. J. Dolan, Associations between aversive learning processes and transdiagnostic psychiatric symptoms in a general population sample. *Nat. Commun.* **11**, 4179 (2020).

38. L. Y. Abramson, M. E. Seligman, J. D. Teasdale, Learned helplessness in humans: Critique and reformulation. *J. Abnorm. Psychol.* **87**, 49–74 (1978).

39. D. A. Clark, Cognitive Reappraisal. *Cogn. Behav. Pract.* **29**, 564–566 (2022).

40. A. H. Mezulis, L. Y. Abramson, J. S. Hyde, B. L. Hankin, Is there a universal positivity bias in attributions? A meta-analytic review of individual, developmental, and cultural differences in the self-serving attributional bias. *Psychol. Bull.* **130**, 711–747 (2004).

41. R. M. Pearson, J. Heron, K. Button, R. P. Bentall, C. Fernyhough, L. Mahedy, L. Bowes, G. Lewis, Cognitive styles and future depressed mood in early adulthood: The importance of global attributions. *J. Affect. Disord.* **171**, 60–67 (2015).

42. J. P. Barber, R. J. DeRubeis, On second thought: Where the action is in cognitive therapy for depression. *Cogn. Ther.* **13**, 441–457 (1989).

43. L. S. Greenberg, *Emotion-focused therapy: Coaching clients to work through their feelings, 2nd ed*, Emotion-focused therapy: Coaching clients to work through their feelings, 2nd ed (American Psychological Association, 2015).

44. N. Haines, Integrating Trait and Neurocognitive Mechanisms of Externalizing Psychopathology: A Joint Modeling Framework for Measuring Impulsive Behavior, Ph.D. thesis, The Ohio State University (2021).

45. C. N. Forbes, New directions in behavioral activation: Using findings from basic science and translational neuroscience to inform the exploration of potential mechanisms of change. *Clin. Psychol. Rev.* **79**, 101860 (2020).

46. P. Cuijpers, M. J. Huibers, The role of common factors in psychotherapy outcomes. *Annu. Rev. Clin. Psychol.* **15**, 207–231 (2019).

47. P. Cuijpers, M. Ciharova, S. Quero, C. Miguel, E. Driessen, M. Harrer, M. Purgato, D. Ebert, E. Karyotaki, The contribution of “Individual Participant Data” meta-analyses of psychotherapies for depression to the development of personalized treatments: A systematic review. *J. Pers. Med.* **12**, 93 (2022).

48. T. A. Furukawa, A. Suganuma, E. G. Ostinelli, G. Andersson, C. G. Beevers, J. Shumake, T. Berger, F. W. Boele, C. Buntrock, P. Carlbring, I. Choi, H. Christensen, A. Mackinnon, J. Dahne, M. J. H. Huibers, D. D. Ebert, L. Farrer, N. R. Forand, D. R. Strunk, I. D. Ezawa, E. Forsell, V. Kaldo, A. Geraedts, S. Gilbody, E. Littlewood, S. Brabyn, H. D. Hadjistavropoulos, L. H. Schneider, R. Johansson, R. Kenter, M. Kivi, C. Björkelund, A. Kleiboer, H. Riper, J. P. Klein, J. Schröder, B. Meyer, S. Moritz, L. Bücker, O. Lintvedt, P. Johansson, J. Lundgren, J. Milgrom, A. W. Gemmill, D. C. Mohr, J. Montero-Marin, J. Garcia-Campayo, S. Nobis, A. C. Zarski, K. O'Moore, A. D. Williams, J. M. Newby, S. Perini, R. Phillips, J. Schneider, W. Pots, N. E. Pugh, D. Richards, I. M. Rosso, S. L. Rauch, L. B. Sheeber, J. Smith, V. Spek, V. J. Pop, B. Ünlü, K. M. P. van Bastelaar, S. van Luenen, N. Garnefski, V. Kraaij, K. Vernmark, L. Warmerdam, A. van Straten, P. Zagorscak, C. Knaevelsrud, M. Heinrich, C. Miguel, A. Cipriani, O. Efthimiou, E. Karyotaki, P. Cuijpers, Dismantling, optimising, and personalising internet cognitive behavioural therapy for depression: A systematic review and component network metaanalysis using individual participant data. *Lancet Psychiatry* **8**, 500–511 (2021)

49. O. V. Ebrahimi, J. Burger, A. Hoffart, S. U. Johnson, Within- and across-day patterns of interplay between depressive symptoms and related psychopathological processes: A dynamic network approach during the COVID-19 pandemic. *BMC Med.* **19**, 317 (2021).

50. M. Craske, Screening and Treatment for Anxiety & Depression (S.T.A.N.D): Alacrity Center Signature Project on Triaging and Adapting to Level of Care, *Clinical trial registration NCT05591937*, clinicaltrials.gov (2022).

51. E. Driessen, Z. D. Cohen, L. Lorenzo-Luaces, S. D. Hollon, D. A. Richards, K. S. Dobson, S. Dimidjian, J. Delgadillo, F. L. Vazquez, K. McNamara, J. J. Horan, P. Gardner, T. P. ' Oei, A. H. P. Mehta, J. W. R. Twisk, I. A. Cristea, P. Cuijpers, Efficacy and moderators of cognitive therapy versus behavioural activation for adults with depression: Study protocol of a systematic review and meta-analysis of individual participant data. *BJPsych Open* **8**, e154 (2022).

52. L. Lorenzo-Luaces, R. E. German, R. J. DeRubeis, It's complicated: The relation between cognitive change procedures, cognitive change, and symptom change in cognitive therapy for depression. *Clin. Psychol. Rev.* **41**, 3–15 (2015).

53. M. I. Eronen, Causal discovery and the problem of psychological interventions. *New Ideas in Psychol.* **59**, 100785 (2020).

54. J. Mason, M. Pownall, A. Palmer, F. Azevedo, Investigating Lay Perceptions of Psychological Measures: A Registered Report, https://psyarxiv.com/jf58q/ (2023).

55. I. A. Cristea, M. J. H. Huibers, D. David, S. D. Hollon, G. Andersson, P. Cuijpers, The effects of cognitive behavior therapy for adult depression on dysfunctional thinking: A meta-analysis. *Clin. Psychol. Rev.* **42**, 62–71 (2015).

56. I. D. Ezawa, S. D. Hollon, Cognitive restructuring and psychotherapy outcome: A meta-analytic review. *Psychotherapy* **60**, 396–406 (2023).

57. H. A. Bear, L. A. Nunes, J. DeJesus, S. Liverpool, B. Moltrecht, L. Neelakantan, E. Harriss, E. Watkins, M. Fazel, Determination of markers of successful implementation of mental health apps for young people: Systematic review. *J. Med. Internet Res.* **24**, e40347 (2022).

58. G. Thornicroft, S. Chatterji, S. Evans-Lacko, M. Gruber, N. Sampson, S. Aguilar-Gaxiola, A. Al-Hamzawi, J. Alonso, L. Andrade, G. Borges, R. Bruffaerts, B. Bunting, J. M. C. d. Almeida, S. Florescu, G. d. Girolamo, O. Gureje, J. M. Haro, Y. He, H. Hinkov, E. Karam, N. Kawakami, S. Lee, F. Navarro-Mateu, M. Piazza, J. Posada-Villa, Y. T. d. Galvis, R. C. Kessler, Undertreatment of people with major depressive disorder in 21 countries. *Br. J. Psychiatry* **210**, 119–124 (2017).

59. J. Torous, S. Bucci, I. H. Bell, L. V. Kessing, M. Faurholt-Jepsen, P. Whelan, A. F. Carvalho, M. Keshavan, J. Linardon, J. Firth, The growing field of digital psychiatry: Current evidence and the future of apps, social media, chatbots, and virtual reality. *World Psychiatry* **20**, 318–335 (2021).

60. S. M. Schueller, J. F. Hunter, C. Figueroa, A. Aguilera, Use of digital mental health for marginalized and underserved populations. *Curr.* **6**, 243–255 (2019).

61. J. Borghouts, E. Eikey, G. Mark, C. D. Leon, S. M. Schueller, M. Schneider, N. Stadnick, K. Zheng, D. Mukamel, D. H. Sorkin, Barriers to and facilitators of user engagement with digital mental health interventions: Systematic review. *J. Med. Internet Res.* **23**, e24387 (2021).

62. D. Szinay, A. Jones, T. Chadborn, J. Brown, F. Naughton, Influences on the uptake of and engagement with health and well-being smartphone apps: Systematic review. *J. Med. Internet Res.* **22**, e17572 (2020).

63. M. G. Craske, M. M. Herzallah, R. Nusslock, V. Patel, From neural circuits to communities: An integrative multidisciplinary roadmap for global mental health. *Nat. Mental Health* **1**, 12–24 (2023).

64. A. Gelman, A. Vehtari, D. Simpson, C. C. Margossian, B. Carpenter, Y. Yao, L. Kennedy, J. Gabry, P.-C. Burkner, M. Modrák, Bayesian Workflow, http://arxiv.org/abs/2011.01808 ´ (2020).

65. J. K. Kruschke, Bayesian Analysis Reporting Guidelines. *Nat. Hum. Behav.* **5**, 1282–1291 (2021).

66. B. Carpenter, A. Gelman, M. D. Hoffman, D. Lee, B. Goodrich, M. Betancourt, M. Brubaker, J. Guo, P. Li, A. Riddell, Stan: A probabilistic programming language. *J. Stat. Softw.* **76**, 1–32 (2017).

67. A. Gelman, D. B. Rubin, Inference from iterative simulation using multiple sequences. *Statistical Science* **7**, 457–472 (1992).

68. N. D. Daw, Trial-by-trial data analysis using computational models. *Decision Making, Affect, and Learning: Attention and Performance XXIII* (Oxford University Press, 2011).

69. R. McElreath, Statistical Rethinking: *A Bayesian Course with Examples in R and Stan* (Chapman and Hall/CRC, New York, 2016).

70. J. Gabry, D. Simpson, A. Vehtari, M. Betancourt, A. Gelman, Visualization in Bayesian workflow. *J. R. Stat. Soc. A. Stat. Soc.* **182**, 389–402 (2019).

71. M. Kay, tidybayes: Tidy Data and Geoms for Bayesian Models, https://zenodo.org/record/5823492 (2022).

72. M. Modrák, A. H. Moon, S. Kim, P. B ´ urkner, N. Huurre, K. Faltejskov ¨ a, A. Gelman, ´ A. Vehtari, Simulation-Based Calibration Checking for Bayesian Computation: The Choice of Test Quantities Shapes Sensitivity, http://arxiv.org/abs/2211.02383 (2022).

73. S. Kim, H. Moon, M. Modrák, T. Säilynoja, SBC: Simulation Based Calibration for ¨ rstan/cmdstanr models, https://hyunjimoon.github.io/SBC/ (2023).

74. C. Hedge, G. Powell, P. Sumner, The reliability paradox: Why robust cognitive tasks do not produce reliable individual differences. *Behav. Res. Methods* **50**, 1166–1186 (2018).

75. K. Kroenke, R. L. Spitzer, J. B. W. Williams, The PHQ-9. *J. Gen. Intern. Med.* **16**, 606–613 (2001).

76. K. M. Connor, K. A. Kobak, L. E. Churchill, D. Katzelnick, J. R. Davidson, Mini-SPIN: A brief screening assessment for generalized social anxiety disorder. *Depress. Anxiety* **14**, 137–140 (2001).

77. Y.-S. Ang, P. Lockwood, M. A. J. Apps, K. Muhammed, M. Husain, Distinct subtypes of apathy revealed by the apathy motivation index. *PLOS One* **12**, e0169938 (2017).

78. C. G. Beevers, D. R. Strong, B. Meyer, P. A. Pilkonis, I. W. Miller, Efficiently assessing negative cognition in depression: An item response theory analysis of the Dysfunctional Attitude Scale. *Psychol. Assess.* **19**, 199–209 (2007).

79. World Health Organization, WHO Disability Assessment Schedule (WHODAS 2.0), https://who.int/standards/classifications/international-classification-of-functioningdisability-and-health/who-disability-assessment-schedule (2012).

80. J. E. J. Buckman, R. Saunders, J. Stott, Z. D. Cohen, L.-L. Arundell, T. C. Eley, S. D. Hollon, T. Kendrick, G. Ambler, E. Watkins, S. Gilbody, D. Kessler, N. Wiles, D. Richards, S. Brabyn, E. Littlewood, R. J. DeRubeis, G. Lewis, S. Pilling, Socioeconomic Indicators of treatment prognosis for adults With Depression: A systematic review and individual patient data meta-analysis. *JAMA Psychiatry* **79**, 406–416 (2022).

81. S. Zorowitz, J. Solis, Y. Niv, D. Bennett, Inattentive responding can induce spurious associations between task behaviour and symptom measures. *Nat. Hum. Behav.* **7**, 1667–1681 (2023).

82. F. Faul, E. Erdfelder, A.-G. Lang, A. Buchner, G*Power 3: A flexible statistical power analysis program for the social, behavioral, and biomedical sciences. *Behav. Res. Methods* **39**, 175–191 (2007).

83. D. Bates, M. Machler, B. Bolker, S. Walker, Fitting linear mixed-effects Mo ¨ dels using lme4. *J. Stat. Softw.* **67**, 1–48 (2015).

84. O. Papaspiliopoulos, G. O. Roberts, M. Skold, A general framework for the parametrization of hierarchical models. *Statistical Science* **22**, 59–73 (2007).

85. J. R. de Leeuw, jsPsych: A JavaScript library for creating behavioral experiments in a Web browser. *Behav. Res. Methods* **47**, 1–12 (2015).

86. L. B. Alloy, L. Y. Abramson, M. E. Hogan, W. G. Whitehouse, D. T. Rose, M. S. Robinson, R. S. Kim, J. B. Lapkin, The temple-wisconsin cognitive vulnerability to depression project: Lifetime history of Axis I psychopathology in individuals at high and low cognitive risk for depression. *J. Abnorm. Psychol.* **109**, 403–418 (2000).

87. P. Kinderman, R. P. Bentall, A new measure of causal locus: The internal, personal and situational attributions questionnaire. *Personal. Individ. Differ.* **20**, 261–264 (1996).

88. B. E. Wisco, S. Nolen-Hoeksema, Interpretation bias and depressive symptoms: The role of self-relevance. *Behav. Res. Ther.* **48**, 1113–1122 (2010).

89. D. S. Yeager, C. J. Bryan, J. J. Gross, J. S. Murray, D. Krettek Cobb, P. H. F. Santos, H. Gravelding, M. Johnson, J. P. Jamieson, A synergistic mindsets intervention protects adolescents from stress. *Nature* **607**, 512–520 (2022).

90. W. G. Hopkins, Individual responses made easy. *J. Appl. Physiol.* **118**, 1444–1446 (2015).

91. A. Norbury, B. Seymour, Response heterogeneity: Challenges for personalised medicine and big data approaches in psychiatry and chronic pain. *F1000Research* **7**, 55 (2018).

92. F. Samejima, Estimation of latent ability using a response pattern of graded scores. *Psychometrika Monograph Supplement* **34**, 1–100 (1969), 97.

93. B. M. Turner, B. U. Forstmann, B. C. Love, T. J. Palmeri, L. Van Maanen, Approaches to analysis in model-based cognitive neuroscience. *J. Math. Psychol.* **76**, 65–79 (2017).

94. N. Haines, T. P. Beauchaine, M. Galdo, A. H. Rogers, H. Hahn, M. A. Pitt, J. I. Myung, B. M. Turner, W.-Y. Ahn, Anxiety modulates preference for immediate rewards among trait-impulsive individuals: A hierarchical bayesian analysis. *Clin. Psychol. Sci.* **8**, 1017–1036 (2020).

95. A. K. Hopkins, R. Dolan, K. S. Button, M. Moutoussis, A reduced self-positive belief underpins greater sensitivity to negative evaluation in socially anxious individuals. *Computational Psychiatry* **5**, 21–37 (2021).

96. S. J. Haberman, When can subscores have value? *JEBS* **33**, 204–229 (2008).

97. K. Kroenke, R. L. Spitzer, J. B. W. Williams, The patient health questionnaire-2. *Med. Care* **41**, 1284–1292 (2003).
